# On the use of sequence-quality information in OTU clustering

**DOI:** 10.7717/peerj.11717

**Published:** 2021-08-16

**Authors:** Robert Müller, Markus Nebel

**Affiliations:** Faculty of Technology, Bielefeld University, Bielefeld, Germany

**Keywords:** Sequence clustering, Operational taxonomic units, Sequence quality information

## Abstract

**Background:**

High-throughput sequencing has become an essential technology in life science research. Despite continuous improvements in technology, the produced sequences are still not entirely accurate. Consequently, the sequences are usually equipped with error probabilities. The quality information is already employed to find better solutions to a number of bioinformatics problems (*e.g*. read mapping). Data processing pipelines benefit in particular (especially when incorporating the quality information early), since enhanced outcomes of one step can improve all subsequent ones. Preprocessing steps, thus, quite regularly consider the sequence quality to fix errors or discard low-quality data. Other steps, however, like clustering sequences into operational taxonomic units (OTUs), a common task in the analysis of microbial communities, are typically performed without making use of the available quality information.

**Results:**

In this paper, we present quality-aware clustering methods inspired by quality-weighted alignments and model-based denoising, and explore their applicability to OTU clustering. We implemented the quality-aware methods in a revised version of our *de novo* clustering tool GeFaST and evaluated their clustering quality and performance on mock-community data sets. Quality-weighted alignments were able to improve the clustering quality of GeFaST by up to 10%. The examination of the model-supported methods provided a more diverse picture, hinting at a narrower applicability, but they were able to attain similar improvements. Considering the quality information enlarged both runtime and memory consumption, even though the increase of the former depended heavily on the applied method and clustering threshold.

**Conclusions:**

The quality-aware methods expand the iterative, *de novo* clustering approach by new clustering and cluster refinement methods. Our results indicate that OTU clustering constitutes yet another analysis step benefiting from the integration of quality information. Beyond the shown potential, the quality-aware methods offer a range of opportunities for fine-tuning and further extensions.

## Introduction

The development of high-throughput sequencing has radically changed the way research is conducted in various biological and medical disciplines, ranging from microbial ecology to personalised medicine. Ongoing advances in sequencing technology have vastly improved the sequencing capacity and read length. However, short-read (*e.g*. SOLiD, Illumina) and especially long-read (*e.g*. PacBio, Oxford Nanopore) technologies are still not error-free. For example, short-read sequencing typically involves error rates between 0.1% and 1%, with the amount and types of errors strongly depending on the used technology (Salk, Schmitt & Loeb, 2018). Illumina sequencing, as an example, suffers mostly from substitutions, *i.e*. base miscalls, caused by similarities between the intensity of the different fluorophores and (pre-)phasing effects (Schirmer et al., 2015). Sequencing machines typically associate the nucleotide sequences with Phred-style quality scores, expressing the estimated error probability at base level, and error-profile analyses have shown that low quality scores can serve as reliable indicators for sequencing errors (Schirmer et al., 2016).

Quality information has been recognised as a valuable resource in order to improve solutions to a range of bioinformatics problems, including sequence assembly, read mapping and denoising. Assembly tools such as Phrap (De la Bastide & McCombie, 2007) use the quality scores to obtain more accurate consensus sequences, while read mappers like BBMap (Bushnell, 2014) and Bowtie 2 (Langmead & Salzberg, 2012) utilise the quality information in the alignment computation or in the assessment of the mapping quality. Denoising algorithms, which aim for distinguishing between biological variation and sequencing errors in order to identify the real biological sequences among the sequenced reads, represent another application area of quality information. For example, DADA2 (Callahan et al., 2016) infers these sequences by employing an error model that integrates both the abundances and quality scores of the reads. Moreover, several alignment methods that directly incorporate quality scores have been proposed over the years; often in (but not necessarily restricted to) the context of read mapping (Clement et al., 2009; Frith, Wan & Horton, 2010; Kim, Kim & Woo, 2008; Malde, 2008). On top of that, whole bioinformatics data processing pipelines can be improved by considering quality information, since downstream analysis steps benefit from an increased data quality. The preprocessing step can include basic quality filters, *e.g*. trimming or even discarding sequences based on the quality scores. More involved pipelines, such as mothur (Schloss et al., 2009), might even use the quality scores to fix errors in the sequences when merging paired-end data.

Clustering sequences into operational taxonomic units (OTUs) constitutes another common step in data analysis pipelines and, thus, already benefits from such quality consideration. The clustering step itself, however, typically ignores the available quality information, leaving room for further improvements. Since the advent of sequencing technologies, OTUs are often used to describe groups of similar nucleotide sequences. In the absence of an agreed-upon bacterial species concept, they also serve as stand-ins for actual taxonomic groups when based on ubiquitous genetic material like the 16S rRNA gene and have become a crucial element in the study of microbial communities (Schmidt, Matias Rodrigues & Von Mering, 2014). OTUs depend heavily on the chosen clustering method and a wide range of approaches with their respective strengths and weaknesses have been proposed over the years (Westcott & Schloss, 2015). Another influential factor is the clustering threshold determining when two sequences are considered similar. Popular thresholds (such as 97% and 99% sequence identity) often used to delineate OTUs at specific taxonomic levels have been derived empirically, but they are controversial and their meaning has evolved over time (Edgar, 2018). Due to these limitations, OTU clustering methods are at risk of combining sequences with different origins, possibly impairing downstream analyses. Addressing this issue, exact sequence variants (ESVs, also known as amplicon sequence variants or zero-radius OTUs) derived from denoising the sequence data have been proposed as an alternative to or even replacement of OTUs (Callahan, McMurdie & Holmes, 2017). Even though ESV methods also cluster sequences in some way, they pursue a quite different approach as inferred exact sequences are only grouped with those reads expected to have occurred solely due to sequencing errors.

In this paper, we present two groups of quality-aware clustering techniques for classic (non-zero-radius) OTUs. The first group comprises adapted and new quality-weighted alignment methods influencing the similarity computation and, thus, results in a straightforward extension of the OTU clustering approach. The second group also aims at the computation of OTUs but it is inspired by the model-based denoising approach of DADA2 and explores whether—despite their differences—the computation of OTUs can benefit from incorporating ideas underlying ESV methods. In order to assess the impact of incorporating quality information on the computation of OTUs, we implemented the quality-aware methods in a revised version of our iterative, *de novo* clustering tool GeFaST. We then evaluated their clustering quality and performance on a range of mock-community data sets in comparison to the quality-unaware methods of GeFaST and other *de novo* clustering tools.

## Basic concepts and previous work

We provide a brief description of the basic concepts underlying our work. Subsequently, we introduce the essential techniques and algorithms constituting the origins of our quality-aware clustering methods presented in the next section.

The focus of this work is the problem of **de novo* sequence clustering*, *i.e*. the grouping of sequences based on their distances among each other. In contrast to other major clustering approaches (closed- and open-reference clustering), the *de novo* approach does not depend on (the existence of) a reference database. We apply the *de novo* clustering to amplicons and, in the following, an *amplicon* refers to a nucleotide sequence (or read) with an identifier and an abundance value (*i.e*. the number of copies of that amplicon). For the sake of the presentation of the quality-aware methods, we further assume that each nucleotide in the sequence of an amplicon is associated with a Phred-style quality score as provided in the FASTQ format. Depending on, *e.g*., the sequencing machine, the quality scores can be stored using different *encodings*. For example, the Illumina 1.8+ encoding allows quality scores between 0 and 41 and adds an offset of 33 to create the encoded scores found in FASTQ files. In the following, we typically refer to the actual quality scores without the offset. As mentioned before, the *Phred score* (Ewing & Green, 1998) can be converted into an error probability of the corresponding nucleotide and these error probabilities are incorporated into the distance between two amplicons. Here, we use the notion of *distance functions* based on pairwise global alignments to formally describe the idea that we derive certain characteristics of such alignments to measure the distance between the involved sequences. Such a distance function could, *e.g*., count the number of edit operations in an optimal alignment or just report its alignment score. We score alignments using *affine gap costs*, assigning a gap of length *k* (*i.e*. a consecutive run of *k* gap symbols in one of the aligned sequences) a score of *g*_*o*_ + *k* · *g*_*e*_, for *g*_*o*_ and *g*_*e*_ the costs of opening and extending a gap, respectively.

### Iterative *de novo* clustering

**Figure 1:**
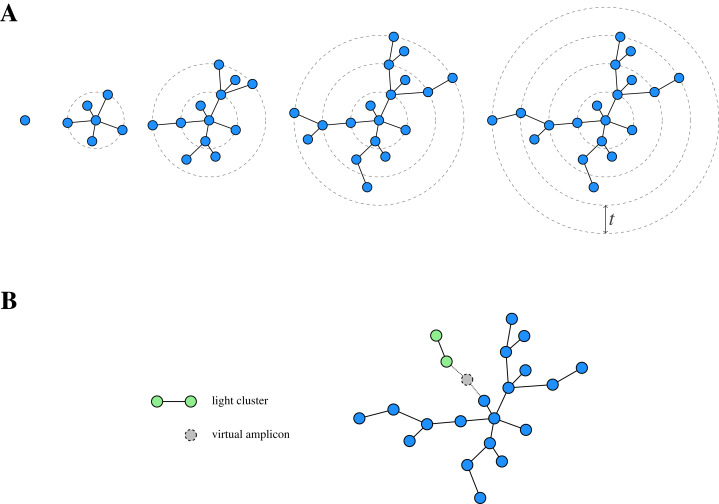
Schematic view of Swarm’s clustering strategy. (A) Starting from a seed, Swarm extends a cluster by iteratively adding amplicons based on a small local threshold *t* until there are no further amplicons which can be connected to it. (B) Light clusters are grafted onto heavy ones during the fastidious step by bridging the gap between them through the assumed existence of virtual linking amplicons. Adapted from Mahé et al. (2015, Fig. 1).

While *de novo* clustering is a popular clustering approach, traditional methods depend on an arbitrary fixed global clustering threshold (Edgar, 2010; Fu et al., 2012), which can be insufficient to accommodate the varying evolutionary speed of different lineages (Mahé et al., 2014). In order to overcome this conceptual issue, the two-phased, agglomerative *de novo* clustering algorithm Swarm (Mahé et al., 2015) was proposed. In contrast to the traditional methods, Swarm uses a (small) local clustering threshold *t* for the number of differences in an optimal alignment and extends a cluster iteratively. In order to cluster a pool (*i.e*. a collection) of amplicons, Swarm proceeds as follows (Fig. 1A): First, the most abundant amplicon is removed from the pool and forms the seed *s* of a new cluster (also referred to as a swarm). Next, all amplicons with at most *t* differences to *s* in an optimal pairwise alignment are also moved from the pool to the current cluster, where they constitute the first generation of subseeds. Subsequently, the same is done for each such subseed to determine the second generation of subseeds and the extension step is iterated until there are no further amplicons which can be added to the swarm. At this point, the current cluster is closed and the overall procedure is repeated on the remaining amplicon pool, again choosing the most abundant amplicon as the seed of the next cluster.

This procedure can lead to long chains of consecutive links and, eventually, over-grouping when connecting different centres of abundance. In order to address this issue, Swarm offers an optional breaking mechanism, prohibiting the connection of two amplicons through less abundant amplicons. To this end, the clustering threshold is complemented by the restriction that the partners (*i.e*. similar amplicons) of the current subseed are also not allowed to have a higher abundance than the subseed itself.

In addition to the clustering phase, Swarm offers a so-called *fastidious* option for *t* = 1, which refines the initially obtained clustering. To this end, Swarm distinguishes between light and heavy swarms based on their mass (*i.e*. the sum of the abundances of the comprised amplicons) and grafts light swarms onto heavy ones by postulating the existence of a (virtual) linking amplicon (Fig. 1B). If such a virtual amplicon bridges the gap of size at most *t*_*f*_ = 2 (with *t*_*f*_ being the fastidious threshold) between the clusters, then all amplicons of the light swarm (but not the virtual amplicon itself) are added to the heavy one.

GeFaST (**Ge**neralised **Fa**stidious **S**warming **T**ool), introduced by Müller & Nebel (2018), reimplements the iterative clustering approach of Swarm (including its breaking mechanism) and generalises its fastidious option. On the one hand, the fastidious refinement is no longer restricted to input threshold *t* = 1. In order to preserve the idea of a virtual linking amplicon, the fastidious threshold *t*_*f*_ = 2 · *t* is used as the default setting. On the other hand, GeFaST offers a more flexible refinement by making the fastidious threshold freely adjustable and independent of *t*. This allows more or less conservative fastidious refinement as needed.

### Incorporating sequence quality per nucleotide

Traditional alignment methods like the Needleman–Wunsch and the Smith–Waterman algorithm (Needleman & Wunsch, 1970; Smith & Waterman, 1981) were developed in a biological context but consider only the nucleotide sequences themselves as they predate the introduction of quality scores (Dear & Staden, 1992). In order to remedy this shortcoming, a range of methods directly incorporating the quality information into the alignment process have been proposed.

Their approaches differ widely, ranging from the assignment of probabilities to different edit operations and adding up the weighted costs (Kim, Kim & Woo, 2008) to using an alternative scoring matrix derived solely from error probabilities (Malde, 2008). Besides the exact way in which the quality information is applied, the methods can also be distinguished in broader terms. Some focus on substitutions because they are the dominant error type in sequences from popular sequencing technologies such as Illumina and, thus, are considered more significant (Schirmer et al., 2016), while others also consider the rarer insertions and deletions. Moreover, not all methods make use of the quality information of both aligned sequences. Especially those developed in a read-mapping context tend to consider only the quality of the read to be mapped. While some methods have been designed for full scoring matrices, others just use more simple scoring functions with fixed costs for an operation (regardless of the participating nucleotides). Lastly, the methods can be distinguished based on the existence of parameters to adjust them to particular use cases. Table 1 provides an overview of major characteristics of the original quality-weighted alignment methods considered for the use in our quality-aware clustering framework.

**Table 1:** Overview of major characteristics of the considered quality-weighted alignment methods. One-sided methods use the quality information of only one sequence. The methods either adapt a provided scoring scheme or completely replace it by an alternative one.

	Clement et al. (2009)	Frith, Wan & Horton (2010)	Kim, Kim & Woo (2008)	Malde (2008)
Weighted operations	Substitutions	Substitutions	All	Substitutions
Use of quality information	One-sided	One-sided	Two-sided	Two-sided
Designed for scoring matrix	Yes	Yes	No	Yes
Type of modification	Adaptation	Adaptation	Adaptation	Replacement
Parameter-free	Yes	No	Yes	No

### Model-based variation detection

Callahan et al. (2016) developed the software package DADA2 for modelling and correcting errors in Illumina-sequenced amplicons. The package implements an improved version of an algorithm called *DADA* (Divisive Amplicon Denoising Algorithm) for inferring the ESVs from a collection of amplicons (Rosen et al., 2012). The algorithm uses a model-based approach for correcting amplicon errors by dividing the collection of dereplicated amplicons into partitions until all of them are consistent with the error model (Algorithm 1). At its core, the algorithm repeatedly searches for the amplicon most inconsistent with its current partition. This amplicon forms the centre of a new partition and other amplicons are allowed to join it when the error model indicates that the new centre is a more likely origin for them. These centres form the representatives of the partitions and are then considered as the ESVs or sample sequences.

**Algorithm 1:** The divisive partitioning algorithm of DADA2 for inferring the composition of a read sample. The dereplicated amplicons are associated with aggregated abundance values and consensus quality profiles.

**Input:***R* = collection of dereplicated amplicon reads, Ω_*A*_ = abundance *p*-value threshold
**Output:** Collection of inferred sample sequences
1 Place all reads in the initial partition *P*_0_ = *R*;
2 Set centre *c*_0_ of *P*_0_ to the most abundant read;
3 **for** *r* ∈ *R* **do**
4 Compute error rate and abundance *p*-value of *r* w.r.t. *c*_0_;
5 **end**/* Let *p_A_*(*r*) be the abundance *p*-value of read *r* w.r.t. to the centre of its current partition. */
6 *i* = 0;
7 **while** min{*p_A_*(*r*) |*r* ∈ *R*} < Ω_*A*_ **do**
8 *i* = *i* + 1;
9 Determine read *s* = arg min{*p_A_*(*r*) |*r* ∈ *R*};
10 Initialise new partition *P_i_* = {*s*} with *c_i_* = *s*;
11 Compute error rate for each non-centre read from *R* w.r.t. *c_i_*;
12 Shuffle each non-centre read to its most likely partition;
13 **end**
14 **return** {*c_0_*, . . . , *c_i_*};

In the following, we describe the formal details of the error model underlying DADA2. The error model depends on transition probabilities $p(a \to b,q)$ between nucleotides *a* and *b*, with *q* being the quality score associated with *b*. These transition probabilities are used to determine *error rates λ*_*c*,*r*_ between two amplicons, a partition centre *c* and a non-centre read *r*, quantifying the rate at which *r* is produced from *c*. To that end, a pairwise sequence alignment between *c* and *r* is computed and the substitutions (*i.e*. matches and mismatches) between the amplicons are collected. Let *S* be the set of substitutions in the alignment between *c* and *r* and let each substitution be represented by a triple (*a*, *b*, *q*), with *a* and *b* a nucleotide from *c* and *r*, respectively, and *q* the quality score of *b*. Assuming that errors occur independently both within and between reads, the computation of the error rate reduces to the product of the transition probabilities of the substitutions in the alignment:

(1) $${\lambda _{c,r}} = \prod\limits_{(a,b,q) \in S} p(a \to b,q).$$

The error rate *λ*_*c*,*r*_ is then used to set up a Poisson model for the abundance of read *r* (denoted as *ab*(*r*)). For independent errors, the number of copies of *r* obtained from *c* is Poisson-distributed with an expectation equal to the product of the error rate and the expected number of reads of *c*. Here, the total abundance of the partition with centre *c* (denoted as *n*_*c*_) is used as that expected number. Using the Poisson model, the *abundance p-value p*_*A*_(*m*, *n*), describing the probability of obtaining at least *m* copies of *r* when amplifying and sequencing *n* copies of centre *c*, is defined as

(2) $${p_A}(m,n): = \displaystyle{1 \over {1 - pois(0;\,n)}} \cdot \sum\limits_{k = m}^\infty pois(k;\,n),$$

for *pois*(*k* ; *n*) the probability mass function of the Poisson distribution with expected value *n*. Parameter *k* describes the number of events occurring within an interval (in our case, copies obtained from amplification and sequencing). Thus, the abundance *p*-value for read *r* and centre *c* is then computed as *p*_*A*_(*ab*(*r*), *n*_*c*_ · *λ*_*c*,*r*_). A (Bonferroni-corrected) abundance *p*-value below a given threshold *Ω*_*A*_ indicates that *r* is not consistent with the error model, as the abundance of *r* is too high to be explained by errors in the sequencing process. Consequently, the read *r* is moved to its own partition if it is the most inconsistent one in the current iteration of the partitioning process in Algorithm 1. In the subsequent shuffling step, the partition might attract other reads if it is the one with the highest expected number of copies of those reads. Once the abundance *p*-values of all reads are above the threshold *Ω*_*A*_, the whole partitioning is considered consistent and the iterative division process stops. By default, DADA2 employs the empirically derived threshold *Ω*_*A*_ = 10^−40^. While this default value is very small, it was found to be appropriate in a range of sequencing experiments by the authors of DADA2 and others (Tsuji et al., 2020).

**Algorithm 2:** Consistency check. Additional test in the consistency-checked clustering methods to decide whether a candidate amplicon *a*, similar to the current subseed *s*, should be added to the current cluster.

ISCONSISTENT (*s, a, E, Ω_A_, N*)
**Input:***s* = subseed amplicon, *a* = candidate amplicon, *E* = error matrix (transition probabilities), Ω_A_ = abundance *p*-value threshold, *N* = number of all amplicons
**Output:** Boolean value indicating whether *a* is consistent with *s*
1 Align *s* and *a* to determine the set of substitutions *S*;
2 Calculate *λ_s,a_* from *S* and *E*; // Equation (1)
3 Determine abundance *p*-value *p_A_*(*ab*(*a*),*λ_s,a_*·*ab*(*s*)); // Equation (2)
4 Compute Bonferroni-corrected abundance *p*-value *p*′*_A_* = *p_A_*(*ab*(*a*),*λ_s,a_*·*ab*(*s*))·*N*;
5 **return** *p′_A_* ≥ Ω_*A*_;
6 **end**

## Quality-aware clustering methods

As outlined above, sequencing is not an error-free process and traditional alignment algorithms use only the called bases and disregard the (typically available) quality information associated with them. Thus, they do not consider the possible reasons for the observed operation in an alignment and, instead, always score it in the same way. Therefore, we want to consider the quality of the called bases with the following intuition: If both bases have high quality scores, we are rather confident in the called bases and the quality weighting should have little effect on the alignment score. On the other hand, consider the case in which we observe, *e.g*., a mismatch between a high-quality and low-quality base. Usual alignment algorithms would apply the full mismatch penalty. With quality weighting, the penalty should, however, be reduced as the chance is increased that the mismatch was caused by a sequencing error in the read with the base of lower quality and that it, possibly, hides an actual match between the sequences. Such a penalty reduction can make the difference between satisfying and exceeding a clustering threshold, especially in clustering scenarios with strict (*i.e*. low) thresholds.

We complement this nucleotide-wise quality awareness by a second, model-supported approach inspired by the denoising method of DADA2. The presented methods incorporate similar consistency checks to explore whether OTU clustering can be enhanced by adopting ideas from the computation of ESVs. By considering quality and abundance information in addition to the sequences themselves, they aim for supporting the decision on whether amplicons should be clustered together. Finally, we describe how the quality-aware methods are implemented in GeFaST.

### Quality-weighted alignments

Our first quality-aware technique comprises a group of methods that directly incorporate the sequence quality into the alignment score. To this end, the scoring functions consider the quality of the participating bases during the alignment process.

In order to simplify the presentation of our quality-weighted methods, we first fix some notations regarding the used scoring functions and the conversion between quality scores and error probabilities. We refer to the sequence alphabet (*e.g*. the set of nucleotides A, C, G and T) as *Σ* and denote its size by |*Σ*|. We further denote the quality score of a character *c* in an aligned sequence as *q*(*c*). Here, the gap symbol−has, by definition, a quality score of zero (*i.e. q*( − ) = 0). As mentioned earlier, the quality score *q*(*c*) of each character in a sequence can be translated into the (base-calling) error probability *p*(*c*), which is given by *p*(*c*) : = 10^−*q*(*c*)/10^. Conversely, the quality score corresponding to an error probability *p* can be expressed as *score*(*p*): = − 10 · log_10_(*p*).

Some of the original methods that we adapt have been developed with respect to maximising scoring functions (*i.e*. the optimal alignment is the one with the highest score). Therefore, if not stated otherwise, we assume that we are initially given a scoring function, which is affine, maximising and has fixed costs for the operations independent on the involved nucleotides. We formally represent the scoring function as a tuple *δ* = (*r*, *m*, *g*_*o*_, *g*_*e*_), consisting of match reward *r*, mismatch penalty *m*, gap-opening penalty *g*_*o*_ and gap-extension penalty *g*_*e*_. Smith, Waterman & Fitch (1981) established a transformation between maximising and minimising scoring functions and, as in Swarm (Mahé et al., 2014), we use this transformation to obtain an equivalent scoring function, producing pairwise alignments identical to the ones found by the original scoring function. The corresponding transformed, minimising scoring function is then similarly represented by *δ*′ = (0, *m*′, *g*_*o*_′, *g*_*e*_′). In the definition of our quality-weighted scoring functions, we also want to incorporate the affine gap-opening penalty and, thus, have to identify positions in an alignment at which a new gap starts (in order to apply the gap-opening penalty). Let the pair (*X*′, *Y*′) be an alignment of two sequences *X* and *Y*. Both aligned sequences *X*′ and *Y*′ can contain gap symbols, and we use a helper function *open*(*X*′, *Y*′, *i*), which returns 1 if and only if a new gap starts at position *i* in the alignment and 0 otherwise. More formally, the function is given by

$$open(X^\prime, Y^\prime, i) := \left\{ \matrix{1,\;\;\;\;\;\;i = 1 \: \land \: (X^\prime_i = - \: \lor \: Y^\prime_i = -) \cr 1,\quad  i > 1 \: \land \: ((X^\prime_i = - \: \land \: X^\prime_{i - 1} \neq -) \: \lor \: (Y^\prime_i = - \: \land \: Y^\prime_{i - 1} \neq -)) \cr\hskip -6.5pc 0,\quad  \textrm{otherwise} }\right.$$

where *X*′_*i*_ and *Y*′_*i*_ denote the *i*-th character in *X*′ and *Y*′, respectively.

Our quality-weighted approach rests on two key components: the quality-weighted alignment score based on a quality-weighted cost function and a boosting function.

**Boosting functions.**The quality-weighted cost functions use error probabilities derived from Phred-style quality scores. Due to the exponential decay of the error probability with increasing quality score, the effect of differences in the quality score can become almost negligible when directly applying the error probabilities as weights in the scoring function. The aim of a boosting function is, thus, to emphasise these differences (especially for higher quality scores) without leaving the range of valid values between 0 and 1.

**Definition 1 (Boosting function)***Let a probability value p ∈ [0, 1] be given. A boosting function*$b(p;\,\theta ): [0,1] \to [0,1]$*maps the value p to another, typically higher probability. Such a mapping can depend on additional parameters, which are listed after a semicolon. The collection of parameters (collectively denoted by θ) can be empty*.

GeFaST currently offers several parameterised boosting functions, which are applied to the (error) probabilities during the computation of the quality-weighted scores.

*No boosting*. If boosted error probabilities are not desired, the identity function can be used to directly hand over the probabilities to the quality-weighted cost function. Thus, the boosting function does not require any parameters: *unboosted*(*p*) : = *p*.

*Multiplicative reinforcement*. Boosting by multiplicative reinforcement involves the multiplication of the error probability with a constant numeric factor *c*. In addition, the multiplied value is restrained from leaving the range of valid probabilities by capping it at one: *mult*(*p*; *c*) : = *min*(*c* · *p*, 1). As a consequence, the exponential decay of the error probability is delayed to higher quality scores.

*Extracting roots and shifting*. In order to keep the exponential decay over the whole range of quality scores but still dampen its effect, the *d*-th root of *p* is computed and the resulting value is shifted by a constant *s* towards zero: $rootshift(p;\,d,s): = max(\root d \of p - s,0)$. Without shifting, especially higher degrees can lead to notably large boosted error probabilities for the highest quality scores in the used encoding. Shifting by *s* allows to reduce this effect, while still benefiting from the weakened exponential decay. We denote a shift as *full* if $s = \root d \of {{p_{max}}}$, with *p*_*max*_ the error probability associated with the highest quality score of the encoding used in the input files. Thus, the roots are shifted such that the boosted error probability for the highest quality score is zero. Moreover, we denote the boosting function for *s* = 0 as *root*(*p*; *d*) : = *rootshift*(*p*; *d*, 0).

*Partially linear decay*. Differences between error probabilities for higher quality scores quickly become very small and, thus, changes of the quality score have almost no effect in that range. Therefore, this boosting function keeps the exponential decay only up to a quality score *s* and switches to a linear decay for higher scores in order to emphasise differences in the quality scores for the higher range. This two-part behaviour can be formally described by

$$linear(p;\:s) = \left\{ \matrix { \hskip -4pcp,  r < s \\ p_s \cdot \frac{l - 1 - s - (r - s)}{l - 1 - s},  \textrm{otherwise} }\right.$$

with *p*_*s*_ the error probability of the quality score at which the behaviour changes, *l* the number of quality levels in the used encoding, and *r* the rank of the quality level corresponding to probability *p* in the encoding.

**Figure 2:**
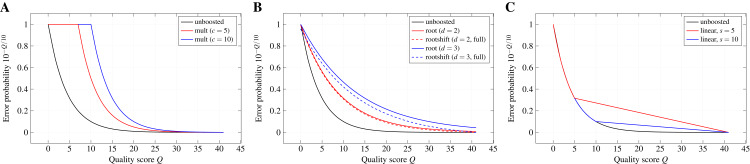
Probability boosting. The effect of (A) multiplicative reinforcement, (B) extracting roots (and shifting) and (C) using a partially linear function on the error probabilities is shown for the Illumina 1.8+ encoding and exemplary boosting parameters.

Figure 2 depicts the effect of the different boosting functions for exemplary parameters.

**Quality-weighted alignment scores**. Similar to the traditional definition of alignment scores, the quality-weighted score is defined as the sum of the scores of the alignment columns. However, the score of an alignment column should now depend on the operation, the participating bases and their quality scores. These quality scores (respectively the error probabilities obtained from them) are used to compute the probability of the observed operation (possibly after boosting) and the score of the alignment column is adapted accordingly. We formalise this notion by extending the traditional column-wise definition of the alignment score as follows:

**Definition 2 (Quality-weighted alignment score)** *Let an alphabet Σ, a scoring function δ and two strings S and T over the alphabet Σ be given. The quality-weighted score of an alignment (S′, T′) of length l of S and T is then defined as the sum of the scores of the alignment columns*

$$\delta_w(S^\prime, T^\prime) := \sum_{i = 1}^{l} w(S^\prime, T^\prime, i ; \delta, \beta_I, \beta_O, \theta)$$

*for a quality-weighted cost function w, which is parameterised by the scoring function δ, an inner boosting function β*_*I*_, *an outer boosting function β*_*O*_*and a possibly empty collection θ of parameters specific to w*.

The definition allows two boosting functions in order to apply the boosting at different levels. The inner boosting function directly processes the individual error probabilities of the nucleotides, while the outer boosting function applies to combined probabilities associated with observing an operation.

Table 2 provides an overview of all quality-weighted cost functions considered in this paper, differing in the alignment operations affected by the quality weighting and their parameterisation. Contrary to some of the original methods shown in Table 1, all adapted versions consider the quality information of both sequences. In this section, we exemplarily describe one of our quality-weighted cost functions in detail and provide its formal definition. Details of the remaining cost functions, including their definitions and how they have been derived, can be found in the supplement (Section A.1).

**Table 2:** Summary of the quality-weighted cost functions implemented in GeFaST. Provides, for each method, the function name (in parentheses), a short description of the underlying idea, the affected operations (‘all’ comprises substitutions, insertions and deletions) and the available parameters.

Method	Synopsis	Subject	Parameters
Clement (*w*_*CL*_)	Score a (mis)match by the linear combination of all possible substitutions, weighted by probabilities.	Substitutions	–
Frith (*w*_*FR*_)	Modify the provided scoring matrix by incorporating error probabilities into the underlying likelihood ratios.	Substitutions	Scaling factor
Malde-A (*w*_*MA*_)	Compute alternative scoring matrix by only using combined error probabilities.	Substitutions	Scaling factor
Malde-B (*w*_*MB*_)	Weight substitution costs directly by the combined error probabilities.	Substitutions	–
Malde-C (*w*_*MC*_)	Weight substitution, insertion and deletion costs directly by the (combined) error probabilities.	All operations	–
Kim-A (*w*_*KA*_)	Score an operation by the linear combination of all possible substitutions, insertions and deletions, weighted by approximated probabilities.	All operations	–
Kim-B (*w*_*KB*_)	Score an operation by the linear combination of all possible substitutions, insertions and deletions, weighted by precise probabilities.	All operations	–
Converge-A (*w*_*CA*_)	Let the costs of the operations converge with decreasing quality against a balance cost positioned between the match reward and the penalties of the other edit operations.	All operations	Balance factor
Converge-B (*w*_*CB*_)	Let the costs of the operations converge with decreasing quality against each other, up to an operation-specific amount.	All operations	Maximum deviations

**Convergence of operation costs**. The following quality-weighting technique considers the quality of both sequences and affects substitutions as well as insertions and deletions. The underlying idea, which—to the best of our knowledge—has not been described before, is that the match score on the one side and the scores of the actual edit operations on the other side should converge towards each other with decreasing sequence quality. Consequently, matches start to incur costs, while mismatches, insertions and deletions are penalised less when using a minimising scoring function.

In order to implement the convergence idea, we have to specify by how much the costs can change at most and how the deviations are related to the sequence quality. For substitutions, the latter is addressed by making the deviations proportional to the probability of the opposite operation. We can compute the probability of an actual mismatch based on the observed agreement of the bases *a* and *b* as

$$\eqalign{  Pr(mismatch\>|\>a = b) = 1 - Pr(match\;|\;a = b) \cr   \quad \quad \quad \quad \quad \quad \quad \quad \;\; = 1 - \underbrace {((1 - {p_a})(1 - {p_b})}_{{\rm{correctly\;called}}} + \underbrace {(|\Sigma | - 1){{{p_a}} \over {|\Sigma | - 1}}{{{p_b}} \over {|\Sigma | - 1}}}_{{\rm{consistently\;miscalled}}}) \cr   \quad \quad \quad \quad \quad \quad \quad  \quad \; =\  {p_a} + {p_b} - {{{p_a}{p_b}|\Sigma |} \over {|\Sigma | - 1}} \cr}$$

and, similarly, the probability of a match when observing a mismatch as

$$\eqalign{  Pr(match\>|\\gt a \ne b) = \underbrace {{{(1 - {p_a}){p_b}} \over {|\Sigma | - 1}} + {{{p_a}(1 - {p_b})} \over {|\Sigma | - 1}}}_{{\rm{single\;miscall}}} + \underbrace {(|\Sigma | - 2){{{p_a}{p_b}} \over {{{(|\Sigma | - 1)}^2}}}}_{{\rm{two\;different\;miscalls}}} \cr  \quad \quad \quad \quad \quad \quad \quad  =\ {{({p_a} + {p_b} - {p_a}{p_b})|\Sigma | - {p_a} - {p_b}} \over {{{(|\Sigma | - 1)}^2}}} \cr}$$

for *p*_*a*_ and *p*_*b*_ the error probabilities of *a* and *b*, respectively. For insertions and deletions, the deviation is proportional to the error probability of the single participating base.

In the following, we present two implementations of our method, differing in how the maximum deviation per operation is determined. The first version considers a balance cost that lies between zero and the smallest penalty in the transformed scoring function *δ*′. The scores of each operation converge towards this balance cost with decreasing quality. The balance cost can be positioned closer to zero (match) or to the penalties, thus determining the maximum deviations, through a balance factor between 0 and 1. Formally, this leads to the following cost function:

**Definition 3 (Converge cost function, Version A)***Let an alphabet Σ, a scoring function δ, an inner boosting function β*_*I*_, *an outer boosting function β*_*O*_*and an alignment (X′, Y′) ∈ (Σ ∪ { − })* * *× (Σ ∪ { − })* * *be given. Further, let b ∈ [0,1] be a balance factor and c*_*b*_ *= b · *min*{m′, g′*_*o*_, *g′*_*e*_*} the corresponding balance cost. The quality-weighted Converge-A cost function for an alignment column is then defined as*

$$\eqalign{
  & {w_{CA}}({X^\prime },{Y^\prime },i;\delta ,{\beta _I},{\beta _O},b) = \left\{ \matrix{
  {c_b} \cdot {\beta _O}{p_{{X^\prime }}} + \left( {{p_{{Y^\prime }}} - {{{p_{{X^\prime }}}{p_{{Y^\prime }}}|\Sigma |} \over {|\Sigma | - 1}}} \right),X_i^\prime  = Y_i^\prime  \hfill \cr 
  {m^\prime } - ({m^\prime } - {c_b}) \cdot {\beta _O}\left( {{{{p_{{X^\prime }}} + {p_{{Y^\prime }}} - {p_{{X^\prime }}}{p_{{Y^\prime }}})|\Sigma | - {p_{{X^\prime }}} - {p_{{Y^\prime }}}} \over {{{(|\Sigma | - 1)}^2}}}} \right),X_i^\prime  \ne Y_i^\prime ,\>X_i^\prime ,Y_i^\prime  \in \Sigma  \hfill \cr 
  g({p_{{Y^\prime }}},i),X_i^\prime  =  -  \hfill \cr 
  g({p_{{X^\prime }}},i),Y_i^\prime  =  -  \hfill \cr}  \right.  \cr 
  & for\;{p_{X'}} = {\beta _I}(p(X_i^\prime ))\;and\;{p_{{Y^\prime }}} = {\beta _I}(p(Y_i^\prime ))\;the\;error\;probabilities\;of\;X_i^\prime \;and\;Y_i^\prime \;andg(p,i)  \cr 
  &  = (g_e^\prime  - (g_e^\prime ) - {c_b} \cdot {\beta _O}(p)) + open({X^\prime },{Y^\prime },i) \cdot (g_o^\prime  - (g_o^\prime ) - {c_b} \cdot {\beta _O}(p)). \cr} $$

The second version of our convergence cost function does not use a balance factor. Instead, it allows to specify a maximum deviation per operation explicitly and, thus, is more flexible as this can, *e.g*., specifically exclude certain operations from the convergence. The corresponding cost function is then formally defined as follows:

**Definition 4 (Converge cost function, Version B)***Let an alphabet Σ, a scoring function δ, an inner boosting function β*_*I*_, *an outer boosting function β*_*O*_*and an alignment (X′, Y′) ∈ (Σ ∪ { − })* * *× (Σ ∪ { − })* * *be given. Further, let* ${m_{ma}},{m_{mi}},{m_{go}},{m_{ge}} \in {\rm {R}}_0^ +$ *be the maximum quality-dependent score deviations for matching, mismatching, opening and extending a gap, respectively. The quality-weighted Converge-B cost function for an alignment column is then defined as*

$${w_{CB}}({X}^{\prime},{Y}^{\prime},i;\,\delta ,{\beta _I},{\beta _O},{m_{ma}},{m_{mi}},{m_{go}},{m_{ge}})$$

$$\eqalignb{= \left\{ {\matrix{ {{m_{ma}} \cdot {\beta _O}\left( {{p_{{X}^{\prime}}} + {p_{{Y}^{\prime}}} - \displaystyle{{{p_{{X}^{\prime}}}{p_{{Y}^{\prime}}}|\Sigma |} \over {|\Sigma | - 1}}} \right),} \hfill  {{{X^{\prime}_{i}}} = {{{Y}^{\prime}}_i}} \hfill \cr {{m}^{\prime} - {m_{mi}} \cdot {\beta _O}\left( {\displaystyle{{({p_{{X}^{\prime}}} + {p_{{Y}^{\prime}}} - {p_{{X}^{\prime}}}{p_{{Y}^{\prime}}})|\Sigma | - {p_{{X}^{\prime}}} - {p_{{Y}^{\prime}}}} \over {{{(|\Sigma | - 1)}^2}}}} \right),} \hfill  {{{X^{\prime}_{i}}} \ne {{Y^{\prime}_{i}}},\,{{X^{\prime}_{i}}},{{{Y}^{\prime}}_i} \in \Sigma } \hfill \cr {g({p_{{Y}^{\prime}}},i),} \hfill  {{{{X^{\prime}_{i}}}} = - } \hfill \cr {g({p_{{X}^{\prime}}},i),} \hfill\hskip -4.4pc {{{Y^{\prime}_{i} = -} }}}}\right. \cr\hskip -27.5pc for \;p_{X^\prime} = \beta_I (p(X^\prime _ i)) \; {and} \; p_{Y^\prime} = \beta_I (p(Y^\prime _ i)) \; the \; error\; probabilities \;of\; X^\prime _ i\ and\ Y^\prime _ i \; and \cr\hskip -27.5pc g(p, i) = (g^\prime _ e - m_{ge} \cdot \beta_O (p)) + open (X^\prime, Y^\prime, i) \cdot (g^\prime _ o - m_{go} \cdot \beta_O (p)) \right.$$

### Model-supported methods

Our second group of quality-aware methods considers the sequence quality in the clustering and refinement phase of the iterative clustering framework in a more general way. Instead of using the quality information to weight alignment scores, these methods are inspired by DADA2’s model-based approach for the inference of ESVs and try to improve OTU clustering by drawing on the notion of consistency.

As previously described, the main idea of DADA2 can be summarised as a split-and-shuffle strategy. Based on the abundance *p*-value, a new partition is created for an amplicon if it is not consistent with its current partition. Subsequently, amplicons are shuffled to the partition that has produced it most likely (based on the expected number of copies of that sequence).

The key idea for using the approach of DADA2 in our OTU clustering scenario is to reverse its strategy in order to help deciding whether (and where) to add amplicons to clusters. While DADA2 removes an amplicon when it is not consistent with its current partition, we check whether, *e.g*., an amplicon is consistent with a cluster to which we would like to add it. If the amplicon is not consistent (*i.e*. DADA2 would rather put it in a separate partition), we abstain from adding it to the cluster (even though it might be similar to the current subseed based on their sequences). Similarly, if there are multiple consistent clusters, we choose the one with the highest expected number of copies.

Here, we focus on the conceptual description of the model-supported clustering and refinement methods. Additional information, including pseudocode descriptions, is available in the supplement (Section A.2).

#### Consistency-checked clustering

The central idea underlying consistency-checked clustering is to complement (or even replace) the purely distance-based decision on whether an amplicon is added to a cluster by an evaluation of the consistency between the current subseed and the candidate amplicon. The aim is to avoid possibly erroneous additions to the cluster by also consulting the consistency model, which involves the sequences and abundances as well as quality information. Algorithm 2 outlines the main steps of the consistency check, whose details have been provided in the description of DADA2. As mentioned there, the default abundance *p*-value threshold of DADA2 was found to be a robust choice. Consequently, we consider it a sensible starting point and also use Ω_*A*_ = 10^−40^ as the default value in GeFaST. In contrast to DADA2, however, GeFaST does not derive the transition probabilities $p(a \to b,q)$ in Eq. (1) from the data (but from quality score *q*) and, by default, only distinguishes between match and mismatch. This simplification reduces the number of parameters and avoids the problem of overfitting the model to the data.

**Clustering amplicons by similarity and consistency. **Our first model-supported clustering method (Algorithm S1) is a direct extension of the classic iterative clustering strategy. As before, an unswarmed amplicon has to be similar to the current (sub)seed with respect to clustering threshold *t*_*c*_ and the used notion of distance (*e.g*. the number of differences in an optimal alignment). In addition, an amplicon deemed similar is now also checked for consistency with respect to the subseed (Algorithm 2). Consequently, only those amplicons that are similar to and consistent with the current subseed are added. Besides the clustering threshold, the method also depends on the abundance *p*-value threshold Ω_*A*_ and can be customised by providing alternative transition probabilities.

**Clustering amplicons by consistency. **The second model-supported clustering method modifies the classic iterative clustering strategy by replacing the distance-based comparison with the consistency check (Algorithm S2). Consequently, this method does not depend on a clustering threshold to determine similar amplicons as candidates. Instead of first determining partners, it directly considers the consistency between the current subseed and each unswarmed amplicon (again Algorithm 2) in order to decide whether it should be added to the current cluster. Thus, the abundance *p*-value threshold Ω_*A*_ effectively replaces the distance threshold *t*_*c*_ here. Analogous to the previous method, custom transition probabilities can be specified to adapt the error model.

Amplicons with abundance one are kept as separate clusters. The Poisson model (Eq. (2)) borrowed from DADA2 always produces an abundance *p*-value of one for those amplicons. Consequently, all of them would be found consistent with the first seed, possibly distorting the clustering. Since each of the singleton clusters is also a light cluster (from the perspective of the fastidious refinement idea), the amplicons can be assigned to the other clusters by using one of the refinement methods presented below.

#### Consistency-guided cluster refinement

Next, we present alternatives to the generalised fastidious refinement method. Similarly, the methods distinguish between light and heavy clusters based on a mass boundary and attempt to attach light clusters to heavy ones. However, the proposed consistency-guided refinement methods do not depend on a distance-based refinement threshold. Instead, a light cluster can only be grafted onto a heavy one if a consistency is detected. Since a light cluster can be consistent with multiple heavy clusters, the final grafting target is determined by choosing the one with the highest expected abundance.

In contrast to the fastidious refinement, some of the consistency-guided refinement methods also offer several options for the handling of light clusters still unattached after the refinement. As outlined in Algorithm S3, the light clusters can be kept as unattached light clusters, discarded, united into a single cluster or reorganised by applying DADA2’s split-and-shuffle strategy to them.

**Attaching light swarms as a whole. **Similar to the distance-based refiners, the first consistency-guided refinement method handles the light clusters as indivisible units. For each light swarm, only its seed *l* is considered and compared to the seeds of the heavy clusters (Algorithm S4). The one showing the highest expected number of copies of *l* is recorded and eventually checked for consistency. The unattached light clusters are then processed by Algorithm S3 according to the chosen option and, subsequently, returned together with the heavy clusters.

**Attaching light-swarm amplicons independently. **The second refinement method does not consider light clusters as indivisible units. Instead, the given light clusters are disassembled in order to reassign the amplicons individually. Algorithm S5 outlines how the method proceeds: For each obtained amplicon, the heavy cluster with the highest expected number of copies of it is determined. The amplicon is attached to that cluster if it is consistent with it. Otherwise the amplicon forms a new, unattached light cluster. Thus, the amplicons are attached independently from the seed and other amplicons that have been in the same light cluster. Analogous to the previous method, the unattached light clusters are processed by Algorithm S3 and then returned together with the heavy clusters.

**Shuffling light-swarm amplicons. **The last refinement method presented here considers light clusters as loosely coupled groups that can be modified (Algorithm S6). The amplicons in a light cluster are again processed individually, with the seed being considered last, but in contrast to the previous method, the cluster is not disassembled right away. For each amplicon, the heavy cluster with the highest expected number of copies is determined. If the amplicon is consistent with that cluster, it is moved to it. Otherwise the amplicon remains in the light cluster. The seed is subject to the additional constraint that it can only leave its cluster if all other members have already been moved to others, then effectively resolving the cluster. When an amplicon leaves the cluster, it might cause a gap in the chain of links underlying the cluster. Thus, if a light cluster is not resolved, the remaining members are rearranged into a new star-shaped cluster with the seed as its centre. In contrast to the other two methods, the new light clusters are not processed subsequently.

### Quality-aware clustering in GeFaST

In order to incorporate the quality-aware methods presented above into GeFaST, its structure has been extensively revised. However, its usage has remained largely unchanged and the tool still provides a command-line interface very similar to Swarm. We outline the new structure of GeFaST in the following.

**Figure 3:**
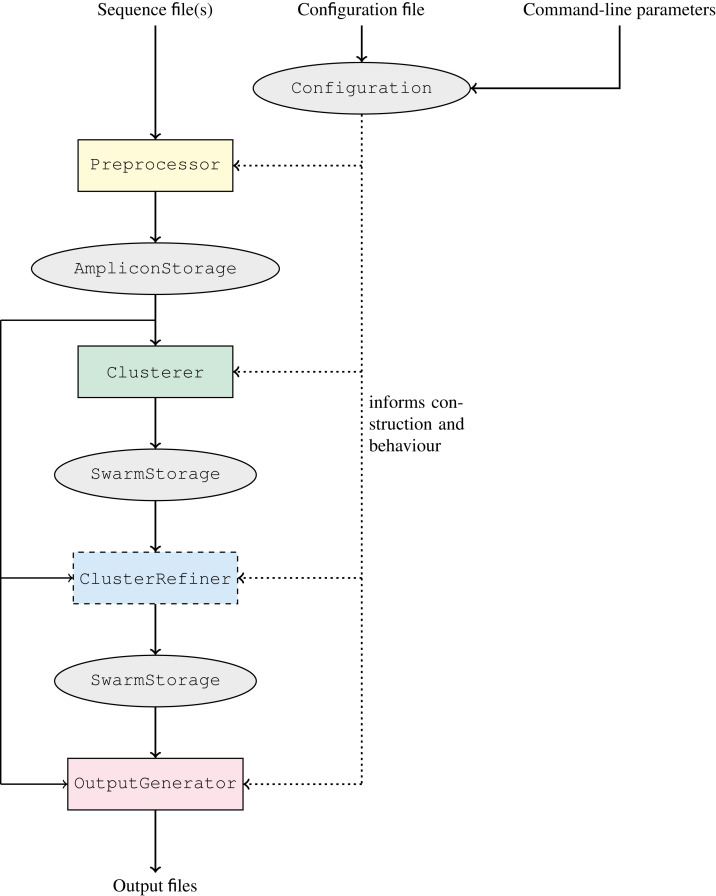
Clustering workflow of GeFaST. The workflow consists of the four phases preprocessing,clustering, cluster refinement (optional) and output generation. The overall execution of each phase is managed by a single, exchangeable component. The different phases communicate only through the amplicon and cluster representations. The actual implementations and their parameters are chosen according to the configuration.

The workflow of GeFaST, producing (structured) clusters from a set of amplicons, comprises up to four now clearly separated phases: preprocessing, clustering, cluster refinement (optional), and output generation (Fig. 3). Each phase is managed by and encapsulated in a different component of GeFaST in order to hide the inner workings from the other phases. Consequently, the components serve as blackboxes to the other components and the major data storages containing the amplicons and clusters, allowing only a predefined collection of intended interactions. Besides the restructured workflow, GeFaST now offers different modes in order to group related clustering techniques and notions of distance between amplicons. The chosen mode influences the Configuration component, which guides the execution of GeFaST and provides the parameters to the different phases. Moreover, the configuration is responsible for managing the construction of the components executing the phases, configuring them appropriately and injecting them into the workflow in order to run GeFaST in the selected mode. Taken together, the new structure allows to exchange implementations and to add new methods like the quality-aware ones more easily.

The two previously available notions of distance based on the number of edit operations, *i.e*. the Levenshtein (or edit) distance and the score-based Levenshtein distance (as used in Swarm), are now grouped into the *Levenshtein mode*. In preparation for the quality-weighted alignment methods, we added the *alignment-score mode*, which uses the score of an optimal alignment (instead of the number of edit operations in it) as the distance between two amplicons. Moreover, clustering amplicons solely by consistency is encapsulated in the *consistency mode* as it does not depend on a distance threshold like the other two modes. The classic iterative clustering strategy as well as its two consistency-checked variants are incorporated as Clusterer implementations. Similarly, the refinement methods are added as ClusterRefiner implementations. The distance-based clusterers and refiners are designed independent of particular distance functions, rather using them as blackboxes. We make use of this flexibility by implementing the quality-weighted alignment methods as distance functions that can be combined with these clusterers and refiners. These distance functions come with several additional parameters and in order to make their use more explicit, we included them in quality-weighted variations of the Levenshtein and alignment-score mode, respectively. The *quality Levenshtein mode* uses the score-based Levenshtein distance with a quality-weighted cost function and computes the distance between amplicons as the number of edit operations in an optimal alignment. Similarly, the *quality alignment-score mode* involves a quality-weighted cost function as well but considers the score of the resulting alignment as the distance.

Detailed information on the usage of the different components and a more technical description of GeFaST are available in the supplement (Section B).

## Evaluation of quality-aware otu clustering

In order to evaluate the effect of incorporating quality information into the OTU clustering process, we conducted several comparative analyses on a range of mock-community data sets. To this end, we determined the clustering quality of our new quality-aware methods on both synthetic and laboratory-determined amplicon data sets covering different hypervariable regions of the 16S rRNA gene and compared it to both quality-unaware iterative clustering and traditional *de novo* methods. The full evaluation workflow, including the commands to prepare the data and execute the analyses, is available in the evaluation repository accompanying the paper.

**Data sets. **The synthetic data sets were obtained as described by Franzén et al. (2015) through *in silico* sequencing using ART (Huang et al., 2011; VanillaIceCream release), simulating paired-end sequencing with the Illumina MiSeq platform. The mock communities assembled by Franzén et al. consist of references from the phylum Bacteroidetes, randomly selected from the Greengenes database (DeSantis et al., 2006, release 13_5) at three different levels of complexity: low (LC, containing 100 references), medium (MC, 250) and high (HC, 500). At each level, 10 mock communities were generated and each mock community was used to obtain two amplicon data sets covering the V3-V4 region (2 × 250 bp reads) and the V4 region (2 × 150 bp reads), respectively. Following the procedure described by Franzén et al., each read pair was turned into a single sequence by concatenating the reverse complement of the second read to the first read.

Moreover, we included two mock communities that were also used by Callahan et al. (2016) in the evaluation of DADA2. The associated amplicons were actually sequenced using the MiSeq platform (2 × 250 bp reads). The balanced mock community contains 59 bacterial and archaeal organisms (represented by 128 reference sequences), with reads coming from the V4 region. In contrast, the hmp community involves reads from the V3-V4, V4 and V4-V5 region, obtained from genomic isolates of 21 bacteria (115 reference sequences). The amplicons of each mock community were used to generate two data sets: single (using only the forward reads) and paired (merging forward and reverse reads).

Table 3 provides further statistics on the data sets. In addition, a more detailed description of the data sets and how they have been generated can be found in the supplement (Section C).

**Table 3:** Overview of the size and quality of the mock-community data sets used in the evaluation. For the different kinds of synthetic data sets, the values are averaged over the respective ten data sets of each kind.

	Data set(s)	Number of reads	Average length	Average quality
Synthetic	LC_V3-V4	1,988	500.0	33.9
	LC_V4	1,988	300.0	35.4
	MC_V3-V4	4,966	500.0	33.9
	MC_V4	4,966	300.0	35.4
	HC_V3-V4	9,958	500.0	33.9
	HC_V4	9,958	300.0	35.4
Laboratory	balanced_single	33,523	220.0	36.8
	balanced_paired	21,808	250.8	40.4
	hmp_single	73,071	220.0	33.6
	hmp_paired	19,882	296.5	37.9

**Comparison of traditional and quality-weighted alignments. **Our first evaluation analysed the effect of incorporating quality-weighted cost functions into the alignment process. To this end, we compared the clustering quality of GeFaST (v2.0.1) in the different quality-unaware and quality-weighted modes. Consequently, the evaluation was divided into two parts. First, we compared the alignment-score mode with the quality alignment-score mode and, then, repeated the analysis for the Levenshtein mode and its quality-weighted counterpart.

In both parts, we combined the respective quality mode with each of the nine quality-weighted cost functions listed in Table 2. Similar to previous analyses, we used Swarm’s and GeFaST’s default scoring function *δ* = (5, −4, −12, −4) throughout this evaluation to investigate the general behaviour of the iterative clustering approach. This default is then transformed into the minimising scoring function *δ*′ = (0, 18, 24, 13) as outlined in description of the quality-weighted alignment methods. In practice, the scoring function should be adapted to the research question and data at hand (States, Gish & Altschul, 1991; Pearson, 2013). We evaluated all cost functions with default parameters in their unboosted form as well as in various boosted variants. Each cost function was combined with the different boosting functions and parameters as follows: *linear* (*s* = 0, 5, …, 35), *mult* (*c* = 5, 10, 15, 20, 50, 100, 250, 500, 1000), *root* (*d* = 2, 3, …, 10) and *rootshift* (full shift, *d* = 2, 3, … 10.) In addition, each unboosted cost function was used in two variants (with weighted and unweighted matches). Thus, there were four variants of each combination of cost function and boosting function (with set parameters), which differed in their handling of the matches as well as in whether the boosting function was used for inner or outer boosting. Consequently, each cost function was considered in 142 variants. For the purpose of a more thorough evaluation, we also included Swarm (v3.0.0) and the classic *de novo* clustering tools USEARCH (Edgar, 2010) and VSEARCH (Rognes et al., 2016). Since GeFaST sorts the input by decreasing abundance, we ran USEARCH (v11.0.667) with the command cluster_fast and option -sort size and used the cluster_size command of VSEARCH (v2.14.2). In addition, we included the UPARSE-OTU algorithm, the recommended OTU clustering method in USEARCH, through the cluster_otus command. Hereinafter, we refer to USEARCH with the cluster_otus command as UPARSE. In order to assess the performance, we considered the variants of GeFaST shown in Table 4 and the other tools as listed above. For the sake of a fair comparison, all tools were run using a single thread.

**Table 4:** Overview of the unboosted and boosted variant considered as the best choice for the different quality-weighted cost functions in quality alignment-score and quality Levenshtein mode. Indicates whether matches are quality-weighted and, for the boosted variant, states the boosting type and function (with the used parameter in parentheses).

Cost function	Quality alignment-score mode			Quality Levenshtein mode		
	Matches	Type	Function	Matches	Type	Function
Clement						
– unboosted	unweighted	–	–	weighted	–	–
– boosted	unweighted	inner	mult (15)	weighted	inner	mult (1000)
Converge-A						
– unboosted	unweighted	–	–	weighted	–	–
– boosted	unweighted	outer	mult (15)	weighted	outer	mult (1000)
Converge-B						
– unboosted	unweighted	–	–	weighted	–	–
– boosted	unweighted	outer	mult (15)	weighted	outer	mult (1000)
Frith						
– unboosted	unweighted	–	–	weighted	–	–
– boosted	weighted	outer	rootshift (2)	weighted	inner	linear (0)
Kim-A						
– unboosted	unweighted	–	–	weighted	–	–
– boosted	unweighted	inner	root (9)	weighted	inner	root (10)
Kim-B						
– unboosted	unweighted	–	–	weighted	–	–
– boosted	unweighted	outer	linear (20)	weighted	inner	root (10)
Malde-A						
– unboosted	weighted	–	–	weighted	–	–
– boosted	weighted	outer	root (3)	weighted	outer	mult (100)
Malde-B						
– unboosted	unweighted	–	–	weighted	–	–
– boosted	unweighted	outer	mult (20)	weighted	inner	mult (1000)
Malde-C						
– unboosted	unweighted	–	–	weighted	–	–
– boosted	unweighted	outer	mult (10)	weighted	inner	rootshift (8)

All tools (except UPARSE) were executed with 10 different clustering thresholds on all the data sets described above, assuming an Illumina 1.8+ quality encoding. The threshold was varied between 1 and 10 when running Swarm or GeFaST in (quality) Levenshtein mode and between 20 and 200 (in steps of 20) in (quality) alignment-score mode. USEARCH and VSEARCH were run with thresholds from 0.99 to 0.90 with a step size of 0.01. UPARSE was used with an identity threshold of 0.97, which cannot be changed.

**Clustering and refinement with a consistency model. **The second evaluation investigated the impact of the consistency model on the iterative clustering strategy in order to assess whether OTU clustering can benefit from ideas coming from the inference of ESVs. The clustering quality of the model-supported clustering and refinement methods was evaluated by running GeFaST in three modes and with different combinations of Clusterer and ClusterRefiner implementations. Both the alignment-score and the Levenshtein mode used the ClassicSwarmer or ConsistentClassicSwarmer in the clustering phase, while the refinement was performed by the FastidiousRefiner, the LightSwarmAppender (LSA), the LightSwarmRefiner (LSR) or the LightSwarmShuffler (LSS). We refer to variants using the ClassicSwarmer and ConsistentClassicSwarmer as *unchecked* and *checked* clustering, respectively. LSA and LSR were evaluated for all four processing options and, when using the FastidiousRefiner, the refinement threshold *t*_*r*_ was obtained by either incrementing or doubling the clustering threshold *t*_*c*_. The consistency mode employed the ConsistencySwarmer in the clustering phase and all the refiners stated above (except the FastidiousRefiner) in the refinement phase. In total, 58 combinations of mode, clusterer and cluster refiner were evaluated. As in the first evaluation, we also included Swarm, USEARCH (cluster_fast command with the option -sort size), VSEARCH (cluster_size command) and UPARSE. While designed for the inference of exact sequences, we also ran DADA2 (v1.10.1) on the data sets and reconstructed the clusters from the inferred sequences and intermediate information on the partitions. Together with the results of the quality-unaware variants of GeFaST, these reconstructed clusters primarily served the assessment of the consistency-based variants combining concepts from both OTU clustering and sequence inference. All variants of GeFaST and the other tools described above were included in the performance evaluation (again using a single thread in each case).

GeFaST, Swarm, USEARCH and VSEARCH were again executed with 10 different clustering thresholds on all the data sets. The clustering threshold ranged from 1 to 10 for Swarm and in Levenshtein mode and from 20 to 200 (with increments of 20) in alignment-score mode. USEARCH and VSEARCH were run with thresholds from 0.99 to 0.90 with a step size of 0.01. DADA2 was executed only once with default parameters on each data set because it does not use a distance threshold, while UPARSE again used its fixed threshold of 0.97.

**Measurement of clustering quality and performance. **We assessed the clustering quality using ground truths and three metrics analogous to previous evaluations of the iterative OTU clustering strategy (Mahé et al., 2014; Müller & Nebel, 2018). As in these previous works, we mainly considered the *adjusted Rand index* (Rand, 1971; Hubert & Arabie, 1985), which measures the agreement between the clusters and the taxonomic assignment and corrects for chance. In addition, we also evaluated the *precision*, quantifying how much amplicons in a cluster also agree in their taxonomic assignment, and the *recall*, measuring the extent to which amplicons with the same taxonomic assignment are clustered together.

The ground truths were derived from the references provided with the data sets. For each synthetic data set, it is obtained by keeping track of the origins of the reads throughout the sequencing simulation. The ground truths of the mock communities were determined by matching the reads against the respective reference sequences using VSEARCH (usearch_global command) with a 97% identity threshold. Section C of the supplement provides additional details on the procedures.

We compared the clustering quality of the different variants (and tools) in terms of their peak performance and their robustness. In order to assess the *maximum* clustering quality of a variant on a given data set, we determined the run leading to the highest adjusted Rand index and recorded the corresponding precision, recall and adjusted Rand index. With respect to the robustness, we first considered the *average* clustering quality over the whole threshold range, recording the average precision, recall and adjusted Rand index over all 10 runs. Since the threshold range was chosen to be quite broad, it might have already contained some thresholds beyond the range viable for the examined variants. Therefore, we also determined the *N-best average* clustering quality, which averages the three quality metrics over the runs with the *N* highest adjusted Rand index values. We considered the N-best average for *N* = 5 in our evaluations.

While focusing on the clustering quality of the presented methods, we also evaluated their performance by measuring the runtime and memory consumption. To this end, we used the GNU time program with the resource specifiers e and M. Specifier e returns the elapsed wall clock time, while M provides the maximum resident set, describing the largest amount of main memory assigned to a program over the course of its execution.

### Clustering based on quality-weighted alignments

In the following, we present the main results of our first evaluation analysing the effect of incorporating quality information directly into the alignment computation. The description of the results is split into two parts, addressing the quality alignment-score and quality Levenshtein mode separately. Table 4 lists the best unboosted and boosted variant per cost function and mode. These are also the variants shown in the quality plots accompanying the descriptions. Additional plots and more detailed results on the different cost functions are provided in the supplement (Section C.2).

#### Quality alignment-score mode

**Figure 4:**
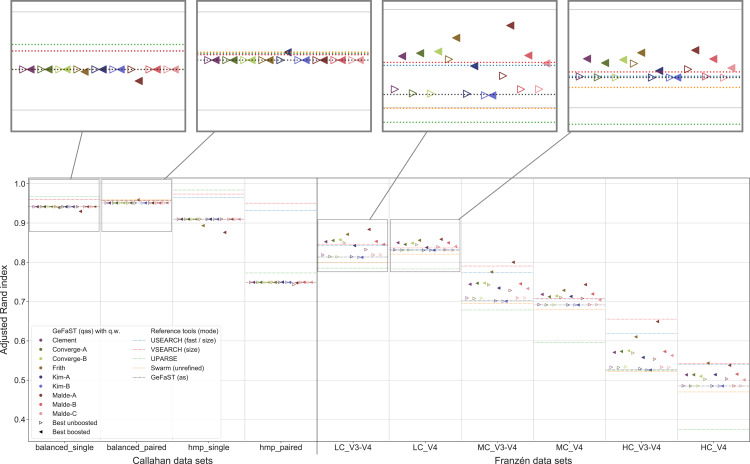
Clustering quality of GeFaST (modes: as, qas), USEARCH, VSEARCH, UPARSE and Swarm on the Callahan and Franzén data sets. Shows the maximum adjusted Rand index of each tool or variant (see Table 4) per data set. For Franzén data, the maxima of the 10 actual data sets per combination of complexity and read type have been averaged.

An overview of the clustering quality of GeFaST on the different data sets when using the quality-weighted cost functions to directly compute the distance between amplicons is provided in Fig. 4. In contrast to the Franzén data sets covered below, the alignment-score mode and its quality-weighted counterpart were mostly identical in terms of the maximum clustering quality on the Callahan data sets, almost regardless of the used quality-weighted cost function. With the exception of a few occasional and small improvements (*e.g*. using the Kim-A cost function on the balanced_paired data set), most variants did not change or (slightly) lowered the clustering quality. The effect on the average clustering quality was similar.

On all Franzén data sets, however, the clustering quality of the unboosted quality-weighted cost functions (in terms of the maximum adjusted Rand index) was already as good as or better than the one of the alignment-score mode. Among the unboosted variants, Frith and Malde-A attained a distinctly higher clustering quality, improving it on average by 4.2% and 2.8%, respectively. The best boosted variant of almost each cost function tended to provide another, even more notable improvement on top of its unboosted counterpart. Frith and Malde-A also showed the highest clustering quality of the boosted variants, increasing the maximum adjusted Rand index on average by 8.3% and 10.4%, respectively, compared to the alignment-score mode. Most of the other cost functions led to increases between 3% and 5%. The unboosted and boosted variant of the Kim-B cost function trailed behind notably, being the only cost function that did not improve on the alignment-score mode. Overall, using the quality-weighted cost functions increased the clustering quality more strongly on the V3-V4 data sets compared to the V4 data sets (at least one and a half times as strong for most cost functions). The observed gains in the adjusted Rand index were typically associated with an increased precision and, on LC and MC data sets, with an at least stable recall. On HC data sets, the recall tended to decrease more often but did not outweigh the increases in precision. The clustering quality of both the alignment-score mode and its quality-weighted equivalent decreased in a similar way with increasing mock-community complexity, but the absolute improvements on the V3-V4 and V4 data sets were relatively stable or grew even slightly.

The quality-weighted cost functions, especially in their boosted variants, also increased the average clustering quality, in particular on the V3-V4 data sets. While V3-V4 and V4 data sets showed a quite similar maximum clustering quality of the alignment-score mode on each level of complexity, the average quality differed considerably due to a large decline on V3-V4 data sets. The boosted quality-weighted cost functions shared this tendency with the alignment-score mode, but most of them were able to reduce the effect by at least half.

From Table 4 it is evident that the different cost functions also favoured different variants to attain their maximum clustering quality. Except for Frith and Malde-A, all cost functions required to exclude matches from the quality weighting to produce meaningful results. Frith and Malde-A, in contrast, were able to handle both weighted and unweighted matches (preferring different boosting functions in each case) but benefited more from including them. The majority of the cost functions worked best with outer boosting and while some cost functions (*e.g*. all Malde versions) attained similar results for inner and outer boosting, others (including the Kim cost functions) were heavily affected by the choice. Multiplicative reinforcement and extracting roots were the most popular boosting functions. The three occurrences of the latter were associated with inner boosting and a high degree (Kim-A) or outer boosting and a small degree (Frith, Malde-A). The multiplicative reinforcement, in turn, was used as an outer boosting function four out of five times and the best boosting parameter lay in a narrow range around 15.

In contrast to the alignment-score mode, GeFaST with quality-weighted cost functions (above all Frith and Malde-A) achieved a similar or even better maximum adjusted Rand index compared to USEARCH and VSEARCH on the different Franzén data sets. These improvements were accompanied by a reduction of the gap to these tools in terms of the precision and, partly, by an additional increase of the already higher recall. Compared to UPARSE and Swarm, the clustering quality of the quality-unware alignment-score mode was already similar or higher and, thus, the quality-weighted cost functions further increased the margins. In addition to the maximum clustering quality, the quality-weighted cost functions led to a similarly improved N-best average adjusted Rand index (in particular on the V3-V4 data sets). However, USEARCH, VSEARCH and UPARSE attained, in total, a higher clustering quality on the Callahan data sets. While GeFaST in quality alignment-score mode (as well as in alignment-score mode) showed a similar maximum and average clustering quality on the balanced data sets, it scored notably lower on the hmp data sets (especially on the paired one). The clustering quality of GeFaST and Swarm was essentially the same on the Callahan data sets, except for a small advantage of Swarm on the balanced_paired data set due to a higher recall.

#### Quality Levenshtein mode

In the second part of the analysis, we considered the clustering quality of GeFaST on the different data sets when using the number of edit operations in alignments based on the quality-weighted cost functions as the distance between amplicons. Similar to the first part, the clustering quality of the quality Levenshtein mode was almost identical to the one of the (quality-unaware) Levenshtein mode on the Callahan data sets. The vast majority of the examined variants did not affect the clustering quality (neither the maximum one nor on average). While we did observe some small improvements, these tended to be rather occasional or were accompanied by adverse results on other data sets.

**Figure 5:**
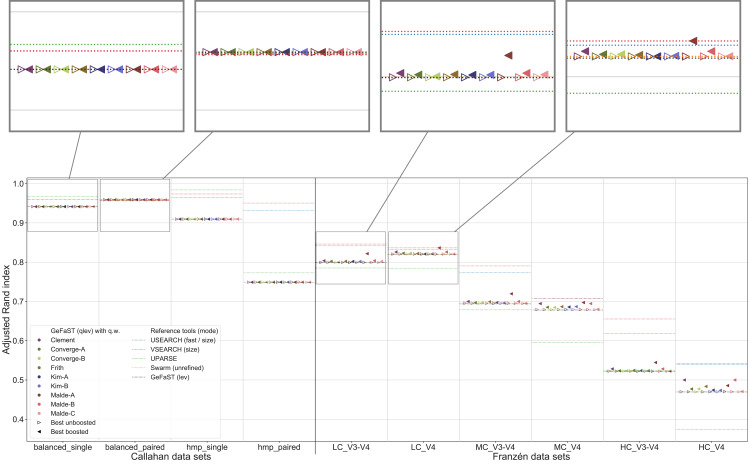
Clustering quality of GeFaST (modes: lev, qlev), USEARCH, VSEARCH, UPARSE and Swarm on the Callahan and Franzén data sets. Shows the maximum adjusted Rand index of each tool or variant (see Table 4) per data set. For Franzén data, the maxima of the 10 actual data sets per combination of complexity and read type have been averaged.

On the contrary, the maximum clustering quality of all unboosted quality-weighted cost functions was equally good or slightly better compared to the Levenshtein mode on Franzén data (Fig. 5). Whereas the unboosted cost functions increased the maximum adjusted Rand index on average by only 0.2%, the best boosted variants improved it up to 3.1%. Malde-A provided the largest gains, followed by Clement and Malde-B with an average increase of 1.8% and Frith with 1.0%. The higher adjusted Rand index usually involved an increased precision and a slightly reduced recall. On the HC_V4 data set, however, the trade-off tended to be in the opposite direction. Both the Levenshtein and the quality Levenshtein mode decreased similarly with increasing mock-community complexity, but the absolute improvements were at least stable for most cost functions (even though the overall differences were small). In addition, the quality Levenshtein mode improved the average clustering quality on Franzén data, especially when using boosted cost functions and on the V4 data sets.

The quality-weighted cost functions again required different variants in order to attain their maximum clustering quality (Table 4). All cost functions worked with both weighted and unweighted matches, but all tended to benefit slightly more from including them. Inner boosting was the preferred choice of the majority of the cost functions, even though the differences between the best variants with inner respectively outer boosting were quite small for most of them. Multiplicative reinforcement and extracting roots (both with and without shifting) were again the preferred boosting functions. The multiplicative boosting function worked best with the highest parameters (except when combined with Malde-A) but showed no clear preference for inner or outer boosting. The root boosting functions were usually associated with high degrees and inner boosting.

Even though the quality Levenshtein mode increased the maximum clustering quality of GeFaST compared to the Levenshtein mode on the Franzén data sets, it was still notably lower than the one of USEARCH and VSEARCH in most cases (in particular on the V3-V4 data sets). In contrast, the maximum clustering quality of the Levenshtein mode was already similar to the one of Swarm and higher than the one of UPARSE. As a consequence, the quality-weighted cost functions created an (even larger) advantage compared to these two tools. Similarly, the (N-best) average clustering quality improved by using quality-weighted cost functions. While the improvements enhanced the advantage of GeFaST on the V4 data sets, USEARCH, VSEARCH and UPARSE continued to be considerably better on the V3-V4 data sets. Overall, they also attained a higher clustering quality on Callahan data. GeFaST (in both Levenshtein modes) achieved a comparable maximum and average clustering quality on the balanced data sets but fell behind USEARCH, VSEARCH and UPARSE on the hmp data sets (especially on the paired one).

#### Performance

GeFaST in Levenshtein mode was faster than Swarm, USEARCH and VSEARCH for large parts of the considered threshold range and outperformed UPARSE notably irrespective of the used threshold (see Fig. S25 in the supplement). The alignment-score mode of GeFaST was notably slower than the Levenshtein mode, especially for larger thresholds. Consequently, it was comparable to the other tools only for the first three to four threshold steps. As expected, using quality-weighted cost functions tended to increase the runtime of GeFaST. Therefore, the quality Levenshtein and quality alignment-score mode typically approached and surpassed the other tools earlier. However, there were notable differences between the cost functions and the two quality modes. Except for the few largest thresholds, most unboosted variants incurred similar and relatively moderate runtime penalties. Averaged over the whole threshold range, all unboosted variants of the quality Levenshtein mode increased the runtime between 10% and 27%. Similarly, the unboosted variants of the quality alignment-score mode led to increases between 14% and 45%. The only exception was Malde-A, which lowered the runtime by more than one third. The boosted variants of the quality Levenshtein mode were slower than their unboosted counterparts. Except for Clement, Malde-B and to some extent Malde-A, their runtime remained quite comparable to the variants without boosting. Compared to the Levenshtein mode, the runtime of the boosted variants was between 14% and 70% higher (not including Clement and Malde-B). In the quality alignment-score mode, however, the boosted variants behaved differently. While Malde-A remained the fastest variant for most thresholds, there are now several variants which were faster than the alignment-score mode for large parts of the threshold range. On average, the boosted variants of Converge-A, Converge-B, Kim-B, Malde-A and Malde-C were between 1% and 40% faster, while the ones of the remaining cost functions increased the runtime by 8% to 22%.

The memory consumption of GeFaST was essentially the same in quality Levenshtein and quality alignment-score mode and was not influenced by the chosen quality-weighted cost function (Fig. S26). The two quality-unaware modes required less memory, with the difference corresponding to the memory occupied by the quality scores. The other tools, especially Swarm and UPARSE, had a considerably lower memory consumption than GeFaST. However, much of the difference was due to the memory-intensive preprocessing of the FASTQ file. When run with the same FASTA file provided to the other tools, the memory consumption of GeFaST was comparable to the one of USEARCH and VSEARCH.

#### Clustering with model-supported methods

Next, we describe the main results of our second evaluation analysing the effect of using the consistency model during the clustering and refinement phases. The supplement (Section C.3) provides additional plots and more detailed results on the different modes.

**Figure 6:**
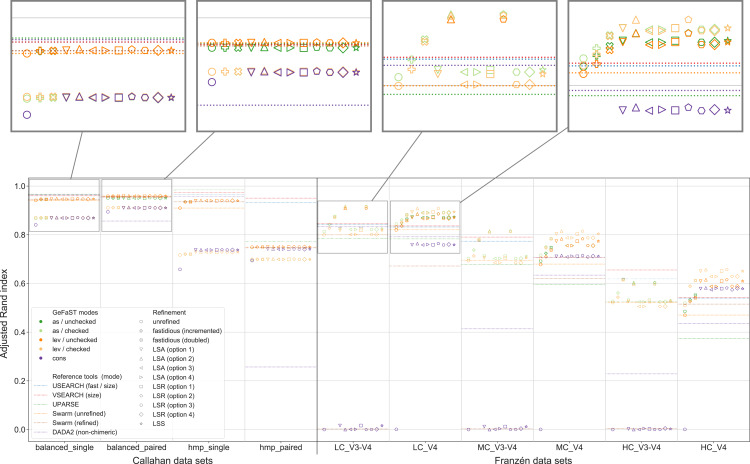
Clustering quality of GeFaST (modes: as, lev, cons), DADA2, USEARCH, VSEARCH, UPARSE and Swarm on the Callahan and Franzén data sets. Alignment-score and Levenshtein mode were evaluated with and without the consistency check in the clustering phase. Shows the maximum adjusted Rand index of each tool or variant per data set. For Franzén data, the average values of the 10 actual data sets per combination of complexity and read type have been averaged.

Figure 6 shows that the model-supported methods were often able to improve the clustering quality compared to the purely distance-based variants of GeFaST, even though there were distinct differences between some of the examined methods. While the consistency mode differed notably from the two distance-based modes, the choice between the alignment-score and the Levenshtein mode did not play a decisive role. The alignment-score mode attained a slightly higher maximum adjusted Rand index on V3-V4 data but tended to be very similar otherwise. The chosen clusterer made a bigger difference, with checked clustering reaching a similar or higher clustering quality on Franzén data, while unchecked clustering showed the clearly better results on Callahan data. The consistency mode was comparable to the distance-based modes with checked clustering on Callahan data but struggled on Franzén data. The unrefined and, on V3-V4 data, the refined variants suffered from massive undergrouping or overgrouping, leading to a clustering quality close to 0. On V4 data, in contrast, the refined variants attained a reasonable clustering quality. It was still lower than the one of the corresponding variants in the distance-based modes but the gap decreased notably with increasing complexity of the data sets.

Both fastidious and consistency-based refinement improved the clustering quality compared to unrefined clustering in the distance-based modes. The fastidious methods increased the maximum adjusted Rand index up to 12.7% and 0.9% on Franzén and Callahan data, respectively. The consistency-based methods, in turn, allowed improvements between 6.0% and 16.7% on Franzén data and between 0.4% and 1.2% on Callahan data. While the fastidious variants worked better on V3-V4 data, the consistency-based variants attained a higher maximum clustering quality on V4 and single data. They also increased the maximum adjusted Rand index of GeFaST in consistency mode by approximately 6% on Callahan data and were essential for obtaining meaningful results on the V4 data sets.

The effect of the different refinement methods tended to be quite similar for the two distance-based modes as well as for unchecked and checked clustering. On V4 data, however, the consistency-based methods notably reduced the difference in clustering quality between the alignment-score and Levenshtein mode observed for the unrefined and fastidious variants. While the consistency-based refinement variants attained almost the same clustering quality on Callahan data and in consistency mode, they formed three groups in the distance-based modes on Franzén data. LSA and LSR reached the highest clustering quality when discarding the remaining light swarms. The second group comprised the option of LSA and LSR keeping all remaining light swarms as well as LSS, while the remaining options of LSA and LSR formed the last group. The consistency-based refinement methods tended to increase the recall and, with the exception of the third group, kept the precision stable or even improved it on most data sets.

In comparison to the other tools, GeFaST in alignment-score and Levenshtein mode (especially with consistency-based refinement) attained a higher clustering quality on V4 data. While the unrefined variants were slightly worse than USEARCH and VSEARCH, their clustering quality exceeded the one of Swarm, UPARSE and DADA2. The fastidious refinement variants tended to surpass the other tools already slightly and the consistency-based refinement variants in alignment-score and Levenshtein mode increased the maximum adjusted Rand index of the other tools by at least 6.2%. On V3-V4 data, however, only the discarding consistency-based variants and the doubling fastidious variants were able to achieve a similar or better clustering quality than USEARCH and VSEARCH, while some variants of the distance-based modes showed a maximum adjusted Rand index that was up to 13.8% lower. Compared to the remaining tools, GeFaST also attained a higher clustering quality on V3-V4 data. As described above, GeFaST in consistency mode struggled on V3-V4 data but produced comparable results on the other data sets. On Callahan data, the best variants of GeFaST (unchecked clustering followed by consistency-based refinement) usually reached a clustering quality similar to or not far below the other tools. Except for hmp_paired, they were at most 4.5% lower. DADA2 was slightly better than the best variants of GeFaST on the single data sets, but GeFaST achieved an at least 4.4% higher clustering quality on the paired data sets.

Compared to the unrefined variants, the different refinement methods substantially lowered the number of clusters computed when reaching their respective maximum clustering quality. The consistency-based refinement methods tended to decrease their number more strongly than the fastidious ones on both Franzén and Callahan data, with an effect potentially being twice as high (or more on Callahan data). Then again, the variants of the second group mentioned above behaved similarly to the doubling fastidious variant on Franzén data. The differences between the alignment-score and the Levenshtein mode were also small in this regard, even though the former led to fewer clusters in some cases (mainly unrefined and fastidious variants). Moreover, variants involving unchecked clustering tended to produce a similar or lower number of clusters than those with checked clustering. GeFaST with consistency-based refinement also led to fewer clusters than USEARCH, VSEARCH and Swarm, while DADA2 and UPARSE usually produced the lowest number of clusters.

The runtime of GeFaST in Levenshtein and alignment-score mode was considerably higher when using consistency-checked clustering, especially for larger thresholds (Fig. S32). The Levenshtein mode remained comparable to the other tools in the lower half of the threshold range, whereas the alignment-score mode exceeded most of them already after the first three threshold steps. When performing a refinement step, the runtime of GeFaST also tended to quickly surpass the one of the other tools. Since checked clustering tended to produce more clusters, the same refinement step was slower when following on checked clustering compared to unchecked clustering. However, the consistency-based variants were similar to each other in terms of their runtime after the same clustering step. Both fastidious and consistency-based refinement could be very time-consuming. The fastidious variants required more time as the threshold increased due to their distance-based nature, while the consistency-based ones were particularly slow for the small thresholds leading to a higher number of clusters. Compared to the distance-based modes with consistency-checked clustering, the consistency mode of GeFaST was even slower (both with and without refinement) and, thus, was also not competitive with the other tools.

As explained for the quality-weighted methods, processing quality information notably increases the memory consumption of GeFaST compared to the other tools. Similarly, those variants with consistency-checked clustering or a consistency-based refinement method showed a higher memory consumption than, *e.g*., unchecked clustering due to the space occupied by the quality scores (Fig. S33). Performing a refinement did not increase the memory consumption and there were only minor differences between Levenshtein and alignment-score mode.

## Discussion

The evaluations in this paper showed that quality-aware methods can improve the clustering quality of the iterative, *de novo* OTU clustering approach. Many of the quality-weighted and model-supported methods implemented in GeFaST were able to attain at least the clustering quality of the quality-unaware variants and to increase it on different data sets, in some cases even notably. Other clustering tools like USEARCH and VSEARCH still achieved better results on several data sets, but the quality-aware methods often reduced the gap to them in these cases and surpassed them (even more) on other data sets. At the same time, several distinct similarities and differences became apparent among the quality-aware methods.

One of the most distinct similarities of both quality-weighted and model-supported methods was their tendency to improve the clustering quality on Franzén data more notably than on Callahan data. While both collections of data sets comprise reads of hypervariable regions of the 16S rRNA gene obtained from bacterial mock communities, they differ in several other respects. Above all, one could argue that the non-synthetic Callahan data sets lend themselves to more viable observations regarding the effect of the quality-aware methods than the synthetically sequenced Franzén data sets. The ground truths of the Franzén data sets, however, allow to evaluate the clustering quality more precisely because they are complete and the origin of each read can be traced back due to the synthetic sequencing. In contrast, only 79 to 86 % of the reads in the Callahan data sets could be assigned taxonomically through searches in the reference databases. Consequently, the Callahan ground truths may not be sufficiently complete or precise to fully recognise the potentially fine-scale effects of the quality-aware methods.

Moreover, the way in which the quality-aware methods typically improved the clustering quality might be another factor contributing to the lower effect on Callahan data. The quality-weighted methods were typically associated with improvements of the precision but it was already very close to the maximum when using quality-unaware methods on Callahan data. Thus, there was little room for improvement and an increased risk of overreaching, resulting in a lower clustering quality due to undergrouping. The behaviour on the balanced_paired data set supports this observation. On this data set, the precision was in general notably lower compared to the other Callahan data sets, thus leaving some room for improvement. At the same time, balanced_paired was the Callahan data set most amenable to improvements. We also observed a similar effect for the model-supported refinement methods, which attained slightly larger improvements on Callahan data (especially on the single data sets). These methods were more strongly related to increasing the recall than the quality-weighted ones and the quality-unaware methods did indeed leave more room for improving the recall than they did for the precision.

Due to its iterative nature, the approach of Swarm and GeFaST is at risk of overgrouping amplicons, eventually leading to a decreased clustering resolution. In terms of our taxonomy-based evaluation, this would manifest itself in the aggregation of different taxonomic groups in a single cluster and, thus, a lower precision. Comparing Swarm and the quality-unaware variants of GeFaST with the other tools, we indeed typically observed a lower precision. As described above, the presented quality-aware methods were often able to reduce or even close the gap to the other tools without lowering the overall clustering quality. Thus, quality-aware methods appear as a way to address this issue.

Our evaluations also showed that the quality-weighted methods had a much larger effect in the quality alignment-score mode than in the quality Levenshtein mode. This did not come as a surprise as the modified scoring functions contribute directly to the distance between the aligned sequences in the quality alignment-score mode, while changes in the alignment score do not necessarily lead to a different or more complex alignment. However, only such changes affect the distance in the quality Levenshtein mode. The fact that the largest improvements in this mode tended to involve more extreme boosting parameters and weighted matches (instead of rather modest parameters and unweighted matches like in the quality alignment-score mode) indicates an attenuated effect too. Nevertheless, the same cost functions (Malde-A and, to some degree, Frith) provided the largest gains in both quality-weighted modes. These two cost functions, in fact, also differ from the others in their approach. Instead of using probability weights or linear combinations directly, their approaches are both related to the likelihood ratios of substitution matrices. Frith and Malde-A were also the only cost functions profiting from weighted matches in the quality alignment-score mode, suggesting that methods based on likelihood ratios are more able to benefit from considering the quality information for both matches and mismatches. The other cost functions appeared to be affected too strongly from the gradually accumulating contributions provided by weighted matches. When considering the alignment score as the distance between two sequences, an excess of match contributions may start to obscure the contributions of the actual differences, thus confusing the clustering process. However, looking at the quality Levenshtein mode, we see that considering weighted matches during the alignment construction could still be beneficial to all cost functions. As explained above, the number of edit operations is more stable than the alignment score and, thus, the clustering process is less prone to be confused by fluctuations in the alignment score due to the contributions from weighted matches. In our evaluation, we also found that emphasising the differences between the quality score with the help of the boosting functions was typically beneficial to the clustering quality. The unboosted variants of the cost functions were also able to provide some improvements but it seemed that without boosting the effect of quality weighting was often too subtle and went unnoticed with respect to the distance function or the clustering threshold. Overall, the multiplicative and root boosting functions were the preferred choices, presumably because they increase the probabilities without fundamentally changing the relationship between quality score and error probability. The initial plateau of the multiplicative boosting function (see Fig. 2) had little impact because correspondingly low quality scores were rare. There were also some general tendencies with respect to the boosting parameter or the application of inner respectively outer boosting, but the combinations considered best were, at least to some degree, specific to the cost function. However, this was not surprising as the cost functions differ at least slightly in their approach and, thus, could not be expected to react in the same way to boosting. At the same time, these best combinations were quite robust and provided improvements on different data sets.

In contrast to the quality-weighted methods, the differences between model-supported methods used with the alignment-score and the Levenshtein mode were minor. Applying a consistency check during the clustering phase did not affect the close similarity of the two modes and, given comparable clusters, the consistency-based refiners performed similar refinements since they are oblivious of the previously used notion of distance. The consistency mode, in turn, performed worse than the distance-based modes as it tended to have problems with finding the balance between undergrouping and overgrouping. Among the model-supported methods, we also observed that some consistency-based refiners consistently increased the clustering quality at least slightly, while adding the consistency check to the clustering phase was not always preferable. In contrast to the Franzén data, checked clustering led to a notably lower clustering quality on Callahan data since its general tendency towards undergrouping by being too restrictive could not be compensated. As discussed above for quality-weighted methods, the already very high precision on these data sets might be a contributing factor. Furthermore, the consistency-based refinement methods turned out to be useful alternatives to the fastidious ones, able to attain a similar clustering quality, while also producing notably fewer clusters. Keeping the unattached clusters after the refinement provided improved and comparable results, while the variants (temporarily) combining the unattached clusters into a single one showed too much overgrouping. Even though the discarding variants look especially promising, their results are not directly comparable to the non-discarding variants and they have to be used with caution. While they usually discarded only a few per cent of the amplicons, they removed more than half of them on some V3-V4 data sets. The V3-V4 data sets also tended to be more difficult to cluster for consistency-based methods in general. In addition to the consistency-based refiners, GeFaST in consistency mode as well as DADA2 also reached a lower clustering quality than on V4 data. A possible reason could be the overall tendency of methods using the consistency model to have difficulties handling reads that cover more than one hypervariable region. Taken together, the evaluation of the model-supported methods showed that the incorporation of ideas from the inference of ESVs into OTU clustering is difficult yet potentially beneficial. The mixed results indicate that further research is necessary to explore the potential of such hybrid methods and fine-tune them.

The aforementioned improvements of the clustering quality obtained by the quality-aware methods were, however, not without cost. The quality-unaware variants of GeFaST were able to attain a better or similar runtime compared to the other tools, whereas the quality-weighted and model-supported methods were usually slower due to often moderate but sometimes severe runtime penalties. Consequently, they will not yet be able to process data sets as large as the quality-unaware methods and tools, even though this heavily depends on the selected method and threshold. Moreover, the other tools (including Swarm) support multithreading to scale up to larger data sets, while this is currently not the case with GeFaST. Similar to Swarm, a parallelisation of parts of the computation is, however, possible.

Based on our evaluation, we advocate the use of quality-aware methods on data sets which tend to be structurally uniform. More precisely, we recommend quality-weighted alignments, in particular the Frith and Malde-A variants with root boosting and weighted matches, when clustering reads coming from the same location, which may span multiple regions of interest (*e.g*. V3-V4 reads). Among these two cost functions, Malde-A was overall more robust. Frith, in turn, relied less on any boosting as its unboosted form already provided considerable improvements close to the boosted variants of all other cost functions except Malde-A. Furthermore, we suggest model-supported refinement, especially the simple LSA variant keeping all ungrafted amplicons, as an alternative to the fastidious method in the opposite scenario, *i.e*. when the read collection can originate from one or more locations but each individual read covers (primarily) only one region of interest (*e.g*. the single data sets). In general, the quality-aware alignments provided better results with the alignment-score mode, while the model-based refinement methods worked similarly well with both alignment-score and Levenshtein mode.

## Conclusions

In this work, we explored the applicability of quality-aware methods in the context of OTU clustering. To this end, we presented a range of quality-weighted and consistency-model supported methods for clustering and cluster refinement and implemented them as extensions of the iterative, *de novo* clustering approach in GeFaST. Our analyses showed that quality-aware methods are indeed able to improve the clustering quality.

These first encouraging findings call for additional analyses, ideally on a variety of data sets, to verify the improvements and to potentially corroborate connections between the characteristics of the data and the (gains in) clustering quality. This might also be combined with the examination of the parameters available for some of the quality-weighted cost functions (*e.g*. whether they can be used to adjust them to certain types of data). The applicability of the quality-aware methods would also benefit from directing further efforts to their performance in order to address the increased runtime and memory consumption. In addition, there is room for improving the quality-aware methods themselves. For example, the quality-weighted alignments could be adapted to consider different base frequencies (Frith, Mori & Asai, 2012). Instead of incorporating the quality information into transformed scoring functions, it might also be a worthwhile effort to investigate the application of the generalised transformation of matches and mismatches of varying degrees hinted at by Smith, Waterman & Fitch (1981). These degrees could be derived from the quality scores of the participating bases and might even consider quality-independent deviations in the error probabilities of different substitutions, *e.g*. observed for transitions and transversions by Tikhonov, Leach & Wingreen (2015).

## Supplemental Information

10.7717/peerj.11717/supp-1 Supplemental Information 1 Supplemental information on methods and results.

## Additional Information and Declarations

### Data Availability

The following information was supplied regarding data availability: GeFaST and the workflow to reproduce the evaluation are available at GitHub: https://github.com/romueller/gefast and https://github.com/romueller/gefast-qa-evaluation. Associated input and result files are available from PUB at https://pub.uni-bielefeld.de/record/2951742.
