# Supplementary material for: On the use of sequence-quality information in OTU clustering
Source: PeerJ. 2021 Aug 16;9:e11717. doi: 10.7717/peerj.11717 (PMC8375510; doi:10.7717/peerj.11717)
Supplement: Supplemental Information 1 [file peerj-09-11717-s001.pdf]

On the use of sequence-quality information in OTU clustering
Robert Müller and Markus E. Nebel

Supplemental information

The following supplements provide additional information on the quality-aware methods (Supplement A) and GeFaST (Supplement B) as well as a more detailed description of the evaluation data sets and results (Supplement C).

A Quality-aware methods

This supplement describes the quality-aware methods in detail, including the formal definition and derivation of the quality-weighted cost functions (Section A.1) and pseudocode descriptions of the model-supported clustering and refinement methods (Section A.2).

A.1 Quality-weighted methods

In the following, we provide the definitions of the remaining quality-weighted cost functions described in Table 2 and describe how they have been derived. In the process, we use the notations introduced in Section "Quality-weighted alignments".

Our clustering framework employs a distance-based notion and, therefore, uses minimising scoring functions with a match score of zero during the alignment computation. Thus, the number of matches has no impact on the alignment score, also reducing the impact of the sequence length. In the quality-weighted context, however, the (number of) matches can make a difference. Even the highest quality scores correspond to non-zero error probabilities and, consequently, even high-quality matches contribute slightly to the alignment score. These gradually accumulating contributions can lead to an undesirable or unexpected dependency between distance threshold and sequence length. Therefore, we propose our quality-weighted cost functions in two forms – with and without quality-weighted matches. As an example, consider the Converge-A cost function from Definition 3 in Section "Quality-weighted alignments":

$$w_{CA}(X',Y',i;\delta,\beta_I,\beta_O,b)=\begin{cases} c_b\cdot\beta_O\left(p_{X'}+p_{Y'}-\frac{p_{X'}p_{Y'}|\Sigma|}{|\Sigma|-1}\right), & X'_i=Y'_i\\ m'-(m'-c_b)\cdot\beta_O\left(\frac{(p_{X'}+p_{Y'}-p_{X'}p_{Y'})|\Sigma|-p_{X'}-p_{Y'}}{(|\Sigma|-1)^2}\right), & X'_i\neq Y'_i,\ X'_i,Y'_i\in\Sigma\\ g(p_{Y'},i), & X'_i=-\\ g(p_{X'},i), & Y'_i=-\end{cases}$$

Its alternative form, abstaining from quality-weighting observed matches, is similarly defined as

$$\widehat{w}_{CA}(X',Y',i;\delta,\beta_I,\beta_O,b)=\begin{cases} 0, & X'_i=Y'_i\\ m'-(m'-c_b)\cdot\beta_O\left(\frac{(p_{X'}+p_{Y'}-p_{X'}p_{Y'})|\Sigma|-p_{X'}-p_{Y'}}{(|\Sigma|-1)^2}\right), & X'_i\neq Y'_i,\ X'_i,Y'_i\in\Sigma\\ g(p_{Y'},i), & X'_i=-\\ g(p_{X'},i), & Y'_i=-\end{cases},$$

differing only in the match case. The alternative forms of the other quality-weighted cost functions are defined analogously and are also denoted using $\widehat{w}$ (instead of w).

A.1.1 Linear combination of substitutions (Clement)

In order to incorporate possible ambiguities originating from the base-calling process (e.g. when different bases show similar probabilities), Clement et al. [1] introduced the read mapper GNUMAP (genomic next-generation universal mapper). Instead of using only the base with the highest probability, their mapper considers the probabilities of all bases in an adapted Needleman-Wunsch algorithm. GNUMAP calculates a probabilistic Needleman-Wunsch score for a read R (provided as a position-weight matrix) and a genomic sequence S by filling the dynamic-programming matrix as follows:

$$NW_{i,j}=\max\begin{cases} NW_{i-1,j-1}+\sum_{k\in\{A,C,G,T\}}PWM(k,j)\cdot cost(k,S_i)\\ NW_{i-1,j}+g\\ NW_{i,j-1}+g\end{cases} \tag{S1}$$

for $PWM(k,j)$ the probability that $R_j = k$, $cost(k, S_i)$ the cost of aligning base k with S_i, and gap cost g. Thus, the quality weighting affects only the substitution costs.

Adjustments for clustering. We have made some modifications in order to adapt the method of Clement et al. for our clustering purposes. To this end, we consider the quality scores of both sequences but use only a single quality score per sequence and position (as provided in, e.g., FASTQ files). In order to assign probabilities to the other, uncalled bases, we distribute the error probability uniformly among them. Moreover, we use affine gap costs instead of linear ones.

Based on the adaptations described above, we substitute the linear combination in Equation (S1) by $m' \cdot Pr(mismatch) = m' \cdot (1 - Pr(match))$, with $Pr(match)$ the probability that the actual bases in the sequences do match (even though the called bases might do not). Consider an alphabet Σ and two characters $a, b \in \Sigma$. Let p_a and p_b be the probabilities that the bases a and b, respectively, have not been called correctly.
The probability of having a match between the actual bases in the sequences given an observed match between the called bases a and b, denoted by $Pr(match \mid a = b)$, comprises the cases that both calls have been correct or that they have been miscalled in the same way:

$$\begin{aligned} Pr(mismatch \mid a = b) &= 1 - Pr(match \mid a = b) \\ &= 1 - \Big(\underbrace{(1 - p_a)(1 - p_b)}_{\text{correctly called}} + \underbrace{(|\Sigma| - 1) \frac{p_a}{|\Sigma| - 1} \frac{p_b}{|\Sigma| - 1}}_{\text{consistently miscalled}} \Big) \\ &= p_a + p_b - \tfrac{p_a p_b |\Sigma|}{|\Sigma| - 1} \end{aligned} \tag{S2}$$

On the other hand, a single miscall affecting either a or b or two different miscalls have to be involved in order to observe a mismatch when the actual bases do match:

$$\begin{aligned} Pr(mismatch \mid a \neq b) &= 1 - Pr(match \mid a \neq b) \\ &= 1 - \Big(\underbrace{\frac{(1 - p_a) p_b}{|\Sigma| - 1} + \frac{p_a (1 - p_b)}{|\Sigma| - 1}}_{\text{single miscall}} + \underbrace{(|\Sigma| - 2) \frac{p_a p_b}{(|\Sigma| - 1)^2}}_{\text{two different miscalls}} \Big) \\ &= 1 - \tfrac{(p_a + p_b - p_a p_b)|\Sigma| - p_a - p_b}{(|\Sigma| - 1)^2} \end{aligned} \tag{S3}$$

With the help of these probabilities, we define the following columnwise cost function that can be used in the computation of a quality-weighted alignment score (see Definition 2):

Definition S1 (Clement cost function) *Let an alphabet Σ, a scoring function δ, an inner boosting function β_I, an outer boosting function β_O and an alignment $(X', Y') \in (\Sigma \cup \{-\})^* \times (\Sigma \cup \{-\})^*$ be given. The quality-weighted* Clement cost function *for an alignment column is then defined as*

$$w_{CL}(X', Y', i; \delta, \beta_I, \beta_O) = \begin{cases} m' \cdot \beta_O \left(p_{X'} + p_{Y'} - \frac{p_{X'} p_{Y'} |\Sigma|}{|\Sigma| - 1} \right), & X'_i = Y'_i \\ m' \cdot \beta_O \left(1 - \frac{(p_{X'} + p_{Y'} - p_{X'} p_{Y'})|\Sigma| - p_{X'} - p_{Y'}}{(|\Sigma| - 1)^2} \right), & X'_i \neq Y'_i \wedge X'_i, Y'_i \in \Sigma \\ g'_e + g'_o \cdot open(X', Y', i), & \textit{otherwise} \end{cases}$$

for $p_{X'} = \beta_I(p(X'_i))$ and $p_{Y'} = \beta_I(p(Y'_i))$ the error probabilities of X'_i and Y'_i.

A.1.2 Modified scoring matrix by adapting likelihood ratios (Frith)

Another technique to integrate per-base quality data when aligning sequences was proposed by Frith et al. [2]. Similar to the approach of Clement et al. described in Section A.1.1, the method was developed in the context of read mapping and, thus, originally only considers the error probabilities of the read sequence. Given a maximising scoring function with a scoring matrix S, they interpret the entries S_{xy}, describing the score for aligning the bases x and y, as log-likelihood ratios

$$S_{xy} = s \cdot \ln(R_{xy}),$$

with s a scaling factor and R_{xy} the likelihood ratio of observing the bases x and y aligned. For a given scaling factor, the likelihood ratios can be computed from the given scoring function as $R_{xy} = \exp(S_{xy}/s)$. A new quality-dependent scoring matrix is then defined by

$$S'_{xd} = s \cdot \ln \left(\sum_{y \in \Sigma} R_{xy} \cdot Pr(y \mid d) \right)$$

for Σ the base alphabet and $Pr(y \mid d)$ the probability that the read base is y given some observation d.

For scoring functions with fixed match reward r and mismatch cost m and quality scores as given in the FASTQ format, Frith et al. describe a simplification of their method. For a called base y with quality score q (as observation d), the quality-dependent probabilities are given by

$$Pr(z \mid y, q) = \begin{cases} 1 - 10^{-q/10}, & z = y \\ 10^{-q/10}/(|\Sigma| - 1), & \text{otherwise.} \end{cases}$$

With the two remaining likelihood ratios $R_{ma} = \exp(r/s)$ and $R_{mi} = \exp(m/s)$ for match and mismatch, respectively, the computation of the quality-dependent scoring matrix simplifies to

$$S'_{xq} = s \cdot \ln \left(Pr(x \mid x, q) \cdot R_{ma} + (1 - Pr(x \mid x, q)) \cdot R_{mi} \right).$$

Adjustments for clustering. As before and similar to a later work by Frith et al. [3], we modify their original method by considering the quality scores of both sequences. Along the lines of Equation (S2), we can compute the probability of an actual match based on the observed agreement of the bases a and b as

$$Pr(match \mid a = b) = 1 - p_a - p_b + \frac{p_a p_b |\Sigma|}{|\Sigma| - 1},$$

with p_a and p_b the error probabilities of a and b, respectively. Given a scoring function δ, the quality-dependent match score is then determined by

$$\begin{aligned} S'_{ma}(p_a, p_b) &= s \cdot \ln \left(Pr(match \mid a = b) \cdot R_{ma} + (1 - Pr(match \mid a = b)) \cdot R_{mi} \right) \\ &= s \cdot \ln \left(\left(1 - p_a - p_b + \tfrac{p_a p_b |\Sigma|}{|\Sigma| - 1} \right) \cdot R_{ma} + \left(1 - \left(1 - p_a - p_b + \tfrac{p_a p_b |\Sigma|}{|\Sigma| - 1} \right) \right) \cdot R_{mi} \right) \end{aligned}$$

for likelihood ratios R_{ma} and R_{mi} as defined above. For a more concise definition of the cost function below, we already incorporate the outer boosting function into the formula for the quality-dependent match score:

$$\begin{aligned} S'_{ma}(p_a, p_b; \beta) &= s \cdot \ln \left(\beta(Pr(match \mid a = b)) \cdot R_{ma} + (1 - \beta(Pr(match \mid a = b))) \cdot R_{mi} \right) \\ &= s \cdot \ln \left(\beta \left(1 - p_a - p_b + \tfrac{p_a p_b |\Sigma|}{|\Sigma| - 1} \right) \cdot R_{ma} + \left(1 - \beta \left(1 - p_a - p_b + \tfrac{p_a p_b |\Sigma|}{|\Sigma| - 1} \right) \right) \cdot R_{mi} \right) \end{aligned}$$

Using Equation (S3), we similarly derive

$$Pr(match \mid a \neq b) = \frac{(p_a + p_b - p_a p_b)|\Sigma| - p_a - p_b}{(|\Sigma| - 1)^2}$$

and, subsequently, the quality-dependent mismatch score

$$\begin{aligned} S'_{mi}(p_a, p_b) &= s \cdot \ln \left(Pr(match \mid a \neq b) \cdot R_{ma} + (1 - Pr(match \mid a \neq b)) \cdot R_{mi} \right) \\ &= s \cdot \ln \left(\tfrac{(p_a + p_b - p_a p_b)|\Sigma| - p_a - p_b}{(|\Sigma| - 1)^2} \cdot R_{ma} + \left(1 - \tfrac{(p_a + p_b - p_a p_b)|\Sigma| - p_a - p_b}{(|\Sigma| - 1)^2} \right) \cdot R_{mi} \right). \end{aligned}$$

As before, we repeat the formula with an included outer boosting function:

$$\begin{aligned} S'_{mi}(p_a, p_b; \beta) &= s \cdot \ln \left(\beta(Pr(match \mid a \neq b)) \cdot R_{ma} + (1 - \beta(Pr(match \mid a \neq b))) \cdot R_{mi} \right) \\ &= s \cdot \ln \left(\beta \left(\tfrac{(p_a + p_b - p_a p_b)|\Sigma| - p_a - p_b}{(|\Sigma| - 1)^2} \right) \cdot R_{ma} + \left(1 - \beta \left(\tfrac{(p_a + p_b - p_a p_b)|\Sigma| - p_a - p_b}{(|\Sigma| - 1)^2} \right) \right) \cdot R_{mi} \right). \end{aligned}$$

In order to obtain a minimising scoring function suitable for our distance-based clustering approach, we use a three-step process. In contrast to the Clement cost function, we cannot directly apply the transformation of the scoring function described by Smith et al. [4] to the quality-dependent scores. This would require a generalisation of the transformation to scoring schemes with multiple match and mismatch scores. According

to Smith et al., such a generalisation is possible but, in our case, it would lead to an inconsistency in the way in which transformed scoring functions are determined and used, thereby reducing the comparability of the different cost functions. Therefore, we first transform the quality-independent scoring function δ as usual. Then, we determine the minimum and maximum (mis)match scores

$$\begin{aligned} L_x &= \min\left\{S'_x(prob(s_1), prob(s_2);\, \beta) \mid s_{min} \leq s_1, s_2 \leq s_{max}\right\} \\ U_x &= \max\left\{S'_x(prob(s_1), prob(s_2);\, \beta) \mid s_{min} \leq s_1, s_2 \leq s_{max}\right\}, \end{aligned} \tag{S4}$$

for $x \in \{ma, mi\}$, dependent on the used quality encoding in order to find the borders of the score ranges. Here, $prob(s)$ represents the error probability associated with a quality score s, while s_{min} and s_{max} denote the smallest and largest quality score in the used encoding. Finally, we map the quality-dependent match and mismatch scores from the range $[L_{ma}, U_{ma}]$ and $[L_{mi}, U_{mi}]$, respectively, to the range $[0, m']$ of the minimising scoring function by considering the relative change

$$\frac{U_x - S'_x(p_a, p_b;\, \beta)}{U_x - L_x} \tag{S5}$$

for $x \in \{ma, mi\}$. For both match and mismatch, the relative change becomes larger when the quality-dependent score tends towards the lower border L_x, which corresponds to a more likely mismatch in both cases. Consequently, we use this relative change as a factor of the mismatch cost m'.

Combining the adapted ideas of Clement et al. with the transformation process above and using affine gap costs, we obtain a suitable cost function:

Definition S2 (Frith cost function) *Let an alphabet Σ, a scoring function δ, an inner boosting function β_I, an outer boosting function β_O and an alignment $(X', Y') \in (\Sigma \cup \{-\})^* \times (\Sigma \cup \{-\})^*$ be given. Further, let $s \in \mathbb{R}^+$ be a scaling factor. The quality-weighted* Frith cost function *for an alignment column is then defined as*

$$w_{FR}(X', Y', i;\, \delta, \beta_I, \beta_O, s) = \begin{cases} \frac{U_{ma} - S'_{ma}(p_{X'}, p_{Y'};\, \beta_O)}{U_{ma} - L_{ma}} \cdot m', & X'_i = Y'_i \\ \frac{U_{mi} - S'_{mi}(p_{X'}, p_{Y'};\, \beta_O)}{U_{mi} - L_{mi}} \cdot m', & X'_i \neq Y'_i,\ X'_i, Y'_i \in \Sigma \\ g'_e + g'_o \cdot open(X', Y', i), & otherwise \end{cases}$$

for $p_{X'} = \beta_I(p(X'_i))$ and $p_{Y'} = \beta_I(p(Y'_i))$ the error probabilities of X'_i and Y'_i.

Yu et al. [5] described a method to determine the scaling factor (and, thus, the likelihood ratios R_{ma} and R_{mi}) corresponding to a given scoring function. For example, the default scoring function of GeFaST with match reward $r = 5$ and mismatch penalty $m = -4$ is associated with a scaling factor $s \approx 5.22113$.

A.1.3 Alternative scoring matrix from error probabilities (Malde)

Malde [6] presented a procedure to adapt alignment algorithms such that they consider the quality information of both sequences. Similar to Frith et al., he considers a scoring matrix of log-likelihood ratios $S_{xy} = s \cdot \ln(R_{xy})$. However, instead of incorporating the quality information into a given scoring function, Malde replaces it by new scores originating solely from error probabilities. For f_z the individual frequency of a base z and q_{xy} the frequency of a substitution of x by y, the likelihood ratios are defined as

$$R_{xy} = \frac{q_{xy}}{f_x \cdot f_y}.$$

Observing that $q_{xy} = f_x \cdot Pr(x \rightarrow y|x)$ and assuming that each base occurs with the same frequency f, the likelihood ratio simplifies to

$$R_{xy} = \frac{f_x \cdot Pr(x \rightarrow y|x)}{f_x \cdot f_y} = Pr(x \rightarrow y|x)/f,$$

with $Pr(x \rightarrow y|x)$ the probability of a substitution from x to y when observing x. For a base alphabet Σ with uniform base frequency $f = \frac{1}{|\Sigma|}$ and a base-independent error probability ϵ, the scoring matrix consists of only two entries:

$$\begin{aligned} S_{xy} &= \begin{cases} s \cdot \ln\left((1-\epsilon)/f\right), & x = y \\ s \cdot \ln\left(\frac{\epsilon}{(|\Sigma|-1)\cdot f}\right), & x \neq y \end{cases} \\ &= \begin{cases} s \cdot \ln\left((1-\epsilon) \cdot |\Sigma|\right), & x = y \\ s \cdot \ln\left(\epsilon \cdot \frac{|\Sigma|}{|\Sigma|-1}\right), & x \neq y \end{cases} \end{aligned} \tag{S6}$$

In order to compute the score of a (mis)match between two bases with error probabilities ϵ_1 and ϵ_2, respectively, the combined error probability

$$err(\epsilon_1, \epsilon_2) := \epsilon_1 + \epsilon_2 - \frac{|\Sigma|}{|\Sigma| - 1} \cdot \epsilon_1 \cdot \epsilon_2 \tag{S7}$$

is used as ϵ in Equation (S6).

In the following, we present three adaptations of Malde's ideas, differing in the affected operations and how the combined error probabilities are used.

Adjustments for clustering (Version A). Our first adapted version computes the alternative scores for match and mismatch as described above (using a notation similar to one in Section A.1.2):

$$\begin{aligned} S'_{ma}(p_a, p_b) &= s \cdot \ln\left((1 - err(p_a, p_b)) \cdot |\Sigma|\right) \\ S'_{mi}(p_a, p_b) &= s \cdot \ln\left(err(p_a, p_b) \cdot \tfrac{|\Sigma|}{|\Sigma|-1}\right) \end{aligned}$$

With an outer boosting function, the corresponding formulae are rewritten as:

$$\begin{aligned} S'_{ma}(p_a, p_b; \beta) &= s \cdot \ln\left((1 - \beta(err(p_a, p_b))) \cdot |\Sigma|\right) \\ S'_{mi}(p_a, p_b; \beta) &= s \cdot \ln\left(\beta(err(p_a, p_b)) \cdot \tfrac{|\Sigma|}{|\Sigma|-1}\right) \end{aligned}$$

As for the method of Frith et al., we have to map these maximising scores to a minimising scoring function but cannot directly apply the transformation to the quality-dependent scores. Therefore, we use the same three-step process to obtain the quality-dependent factors of mismatch cost m' (see Equations (S4) and (S5)). Using affine gap costs, we obtain the following cost function:

Definition S3 (Malde cost function, Version A) *Let an alphabet Σ, a scoring function δ, an inner boosting function β_I, an outer boosting function β_O and an alignment $(X', Y') \in (\Sigma \cup \{-\})^* \times (\Sigma \cup \{-\})^*$ be given. Further, let $s \in \mathbb{R}^+$ be a scaling factor. The quality-weighted* Malde-A cost function *for an alignment column is then defined as*

$$w_{MA}(X', Y', i; \delta, \beta_I, \beta_O, s) = \begin{cases} \frac{U_{ma} - S'_{ma}(p_{X'}, p_{Y'}; \beta_O)}{U_{ma} - L_{ma}} \cdot m', & X'_i = Y'_i \\ \frac{U_{mi} - S'_{mi}(p_{X'}, p_{Y'}; \beta_O)}{U_{mi} - L_{mi}} \cdot m', & X'_i \neq Y'_i,\ X'_i, Y'_i \in \Sigma \\ g'_e + g'_o \cdot open(X', Y', i), & otherwise \end{cases}$$

for $p_{X'} = \beta_I(p(X'_i))$ and $p_{Y'} = \beta_I(p(Y'_i))$ the error probabilities of X'_i and Y'_i.

A typical choice for the scaling factor is $s = \frac{1}{\ln(2)}$, scaling the costs to bit units [6]. The method of Yu et al. [5] does not apply here, because the likelihood ratios of the original scoring function are not required.

Adjustments for clustering (Version B). The second version also considers the combined error probability from Equation (S7). However, instead of computing an alternative scoring matrix (Equation (S6)), the probabilities $Pr(x \to y|x)$ are directly used as quality-dependent factors of the mismatch cost m'. The gap costs remain unweighted as in Version A.

Definition S4 (Malde cost function, Version B) *Let an alphabet Σ, a scoring function δ, an inner boosting function β_I, an outer boosting function β_O and an alignment $(X', Y') \in (\Sigma \cup \{-\})^* \times (\Sigma \cup \{-\})^*$ be given. The quality-weighted* Malde-B cost function *for an alignment column is then defined as*

$$w_{MB}(X', Y', i; \delta, \beta_I, \beta_O) = \begin{cases} m' \cdot \beta_O(err(p_{X'}, p_{Y'})), & X'_i = Y'_i \\ m' \cdot \left(1 - \frac{\beta_O(err(p_{X'}, p_{Y'}))}{|\Sigma|-1}\right), & X'_i \neq Y'_i,\ X'_i, Y'_i \in \Sigma \\ g'_e + g'_o \cdot open(X', Y', i), & otherwise \end{cases}$$

for $p_{X'} = \beta_I(p(X'_i))$ and $p_{Y'} = \beta_I(p(Y'_i))$ the error probabilities of X'_i and Y'_i.

Adjustments for clustering (Version C). The last adaptation of the ideas of Malde is an extension of Version B. We again directly use the combined error probability as a quality-dependent factor of the substitution costs. In addition, insertions and deletions are now also weighted by using the error probability of the single participating base. With decreasing quality of that base, the gap costs are also decreasing.

Definition S5 (Malde cost function, Version C) *Let an alphabet* Σ*, a scoring function* δ*, an inner boosting function* β_I*, an outer boosting function* β_O *and an alignment* $(X', Y') \in (\Sigma \cup \{-\})^* \times (\Sigma \cup \{-\})^*$ *be given. The quality-weighted* Malde-C cost function *for an alignment column is then defined as*

$$w_{MC}(X', Y', i; \delta, \beta_I, \beta_O) = \begin{cases} m' \cdot \beta_O(err(p_{X'}, p_{Y'})), & X'_i = Y'_i \\ m' \cdot \left(1 - \frac{\beta_O(err(p_{X'}, p_{Y'}))}{|\Sigma|-1}\right), & X'_i \neq Y'_i,\ X'_i, Y'_i \in \Sigma \\ (1 - \beta_O(p_{Y'})) \cdot (g'_e + g'_o \cdot open(X', Y', i)), & X'_i = - \\ (1 - \beta_O(p_{X'})) \cdot (g'_e + g'_o \cdot open(X', Y', i)), & Y'_i = - \end{cases}$$

for $p_{X'} = \beta_I(p(X'_i))$ *and* $p_{Y'} = \beta_I(p(Y'_i))$ *the error probabilities of* X'_i *and* Y'_i*.*

A.1.4 Linear combination of all edit operations (Kim)

Kim et al. [7] proposed an adaptation of the sequence alignment considering quality information of both sequences. For a maximising scoring function with fixed scores for matches, mismatches and gaps (r, m and g, respectively), the score of an alignment column (x, y) is calculated as the probability-weighted linear combination of the different operations

$$S(x, y) = r \cdot Pr(match \mid x, y) + m \cdot Pr(mismatch \mid x, y) + g \cdot Pr(gap \mid x, y) \tag{S8}$$

with probabilities depending on the operation observed in that alignment column.

Consider an alphabet Σ (without the gap symbol $-$) and two characters $a, b \in \Sigma$. Let p_a and p_b be the probabilities that the bases a and b, respectively, have not been called correctly. In order to assign probabilities to the other, uncalled bases at the position in the respective string, we distribute the error probability uniformly among them and the gap symbol.

As mentioned above, the probabilities for Equation (S8) depend on the observed bases a and b. When observing a match, the probabilities of the actual relationship between the sequences at that position (match, mismatch or gap) are computed as follows:

$$Pr(match \mid a = b) = \underbrace{(1 - p_a)(1 - p_b)}_{\substack{\text{correctly called}\\ \text{as match}}} + \underbrace{(|\Sigma| - 1)\frac{p_a \cdot p_b}{|\Sigma|^2}}_{\substack{\text{consistently miscalled}\\ \text{as another match } (\triangle)}} \tag{S9}$$

$$Pr(mismatch \mid a = b) = \underbrace{(|\Sigma| - 1)\frac{(1 - p_a)p_b}{|\Sigma|} + (|\Sigma| - 1)\frac{p_a(1 - p_b)}{|\Sigma|}}_{\text{single miscall}} + \underbrace{(|\Sigma| - 1)(|\Sigma| - 2)\frac{p_a \cdot p_b}{|\Sigma|^2}}_{\text{two different miscalls } (\triangle)} \tag{S10}$$

$$Pr(gap \mid a = b) = \underbrace{(1 - p_a)\frac{p_b}{|\Sigma|} + \frac{p_a}{|\Sigma|}(1 - p_b)}_{\text{single gap, no miscall}} + \underbrace{(|\Sigma| - 1)\frac{p_a \cdot p_b}{|\Sigma|^2} + (|\Sigma| - 1)\frac{p_a \cdot p_b}{|\Sigma|^2}}_{\text{single gap, other base miscalled } (\triangle)} \tag{S11}$$

Similarly, we compute the probabilties of actually having a match, mismatch and gap between the sequences for an observed mismatch as:

$$Pr(match \mid a \neq b) = \underbrace{\frac{(1 - p_a)p_b}{|\Sigma|} + \frac{p_a(1 - p_b)}{|\Sigma|}}_{\text{single miscall}} + \underbrace{(|\Sigma| - 2)\frac{p_a \cdot p_b}{|\Sigma|^2}}_{\text{two different miscalls } (\triangle)} \tag{S12}$$

$$Pr(mismatch \mid a \neq b) = \underbrace{(1-p_a)(1-p_b)}_{\text{mismatch correctly called}}$$
$$+ \underbrace{(|\Sigma|-2)\frac{(1-p_a)p_b}{|\Sigma|} + (|\Sigma|-2)\frac{p_a(1-p_b)}{|\Sigma|}}_{\text{single miscall not creating a match}} + \underbrace{((|\Sigma|-1)^2 - (|\Sigma|-2))\frac{p_a \cdot p_b}{|\Sigma|^2}}_{\text{two miscalls not creating a match } (\triangle)} \tag{S13}$$

$$Pr(gap \mid a \neq b) = \underbrace{(1-p_a)\frac{p_b}{|\Sigma|} + \frac{p_a}{|\Sigma|}(1-p_b)}_{\text{single gap, no miscall}} + \underbrace{(|\Sigma|-1)\frac{p_a \cdot p_b}{|\Sigma|^2} + (|\Sigma|-1)\frac{p_a \cdot p_b}{|\Sigma|^2}}_{\text{single gap, other base miscalled } (\triangle)} \tag{S14}$$

Lastly, we consider the corresponding probabilities when observing an insertion or deletion. Kim et al. model the match and mismatch probability as zero because an observed gap symbol is considered as fixed, leaving no probability over for the other bases in that sequence.

$$Pr(match \mid gap) = 0 \tag{S15}$$
$$Pr(mismatch \mid gap) = 0 \tag{S16}$$
$$Pr(gap \mid gap) = \underbrace{\begin{cases} 1 - \frac{p_b}{|\Sigma|}, & a = - \\ 1 - \frac{p_a}{|\Sigma|}, & b = - \end{cases}}_{\text{other base not also a gap}} \tag{S17}$$

Kim et al. subsequently simplify their equations by ignoring terms with presumably negligible values (marked with ($\triangle$) in above equations). Therefore, we describe our adaptations in two versions, differing in the use of the marked terms.

Adjustments for clustering (Version A). We incorporate the method of Kim et al. by reusing their idea of a probability-weighted linear combination of the operation scores. However, since we are eventually using the transformed minimising scoring function with a match score of zero, we do not need the probability for actually having a match between the sequences at the respective position. For our first adaptation, we follow Kim et al. and do not use the parts of the equations marked with ($\triangle$).
Using Equations (S10) and (S11), we obtain the probability weights

$$\begin{aligned} P_{mi \leftarrow ma}(p_a, p_b) &= (|\Sigma|-1)\tfrac{(1-p_a)p_b}{|\Sigma|} + (|\Sigma|-1)\tfrac{p_a(1-p_b)}{|\Sigma|} \\ P_{g \leftarrow ma}(p_a, p_b) &= (1-p_a)\tfrac{p_b}{|\Sigma|} + \tfrac{p_a}{|\Sigma|}(1-p_b) \end{aligned}$$

for mismatch and gap when observing a match between bases a and b with respective error probabilities p_a and p_b. For an oberved mismatch, the weights

$$\begin{aligned} P_{mi \leftarrow mi}(p_a, p_b) &= (1-p_a)(1-p_b) + (|\Sigma|-2)\tfrac{(1-p_a)p_b}{|\Sigma|} + (|\Sigma|-2)\tfrac{p_a(1-p_b)}{|\Sigma|} \\ P_{g \leftarrow mi}(p_a, p_b) &= (1-p_a)\tfrac{p_b}{|\Sigma|} + \tfrac{p_a}{|\Sigma|}(1-p_b) \end{aligned}$$

are similarly derived from Equations (S13) and (S14). Since the probability of a mismatch is zero when observing a gap, we only need

$$P_{g \leftarrow g}(p_a, p_b, a, b) = \begin{cases} 1 - \frac{p_b}{|\Sigma|}, & a = - \\ 1 - \frac{p_a}{|\Sigma|}, & b = - \end{cases},$$

obtained from Equation (S17), in this last case.

Using affine gap costs instead of linear ones, we then define the following cost function:

Definition S6 (Kim cost function, Version A) *Let an alphabet Σ, a scoring function δ, an inner boosting function β_I, an outer boosting function β_O and an alignment $(X', Y') \in (\Sigma \cup \{-\})^* \times (\Sigma \cup \{-\})^*$ be given. The quality-weighted* Kim-A cost function *for an alignment column is then defined as*

$$w_{KA}(X', Y', i; \delta, \beta_I, \beta_O) = \begin{cases} m' \cdot \beta_O(P_{mi \leftarrow ma}(p_{X'}, p_{Y'})) + g(i) \cdot \beta_O(P_{g \leftarrow ma}(p_{X'}, p_{Y'})), & X'_i = Y'_i \\ m' \cdot \beta_O(P_{mi \leftarrow mi}(p_{X'}, p_{Y'})) + g(i) \cdot \beta_O(P_{g \leftarrow mi}(p_{X'}, p_{Y'})), & X'_i \neq Y'_i,\ X'_i, Y'_i \in \Sigma \\ g(i) \cdot \beta_O(P_{g \leftarrow g}(p_{X'}, p_{Y'}, X'_i, Y'_i)), & \textit{otherwise} \end{cases}$$

for $p_{X'} = \beta_I(p(X'_i))$ and $p_{Y'} = \beta_I(p(Y'_i))$ the error probabilities of X'_i and Y'_i and $g(i) = g'_e + g'_o \cdot open(X', Y', i)$.

Adjustments for clustering (Version B). Our second adaptation corresponds to the first one, except that the probability weights now also include the previously neglected terms marked with $(\triangle)$ in Equations (S9) to (S14).
Consequently, we obtain the following weights for the case of observing a match:

$$P^{(\triangle)}_{mi \leftarrow ma}(p_a, p_b) = (|\Sigma| - 1)\frac{(1-p_a)p_b}{|\Sigma|} + (|\Sigma| - 1)\frac{p_a(1-p_b)}{|\Sigma|} + (|\Sigma| - 1)(|\Sigma| - 2)\frac{p_a \cdot p_b}{|\Sigma|^2}$$

$$P^{(\triangle)}_{g \leftarrow ma}(p_a, p_b) = (1 - p_a)\frac{p_b}{|\Sigma|} + \frac{p_a}{|\Sigma|}(1 - p_b) + 2(|\Sigma| - 1)\frac{p_a \cdot p_b}{|\Sigma|^2}$$

The weights for an observed mismatch change to

$$\begin{aligned} P^{(\triangle)}_{mi \leftarrow mi}(p_a, p_b) &= (1 - p_a)(1 - p_b) + (|\Sigma| - 2)\frac{(1-p_a)p_b}{|\Sigma|} \\ &\quad + (|\Sigma| - 2)\frac{p_a(1-p_b)}{|\Sigma|} + ((|\Sigma| - 1)^2 - (|\Sigma| - 2))\frac{p_a \cdot p_b}{|\Sigma|^2} \end{aligned}$$

$$P^{(\triangle)}_{g \leftarrow mi}(p_a, p_b) = (1 - p_a)\frac{p_b}{|\Sigma|} + \frac{p_a}{|\Sigma|}(1 - p_b) + 2(|\Sigma| - 1)\frac{p_a \cdot p_b}{|\Sigma|^2},$$

while the weight for the last case remains unchanged:

$$P^{(\triangle)}_{g \leftarrow g}(p_a, p_b, a, b) = \begin{cases} 1 - \frac{p_b}{|\Sigma|}, & a = - \\ 1 - \frac{p_a}{|\Sigma|}, & b = - \end{cases}.$$

This leads to the following, adapted cost function:

Definition S7 (Kim cost function, Version B) *Let an alphabet Σ, a scoring function δ, an inner boosting function β_I, an outer boosting function β_O and an alignment $(X', Y') \in (\Sigma \cup \{-\})^* \times (\Sigma \cup \{-\})^*$ be given. The quality-weighted* Kim-B cost function *for an alignment column is then defined as*

$$w_{KB}(X', Y', i; \delta, \beta_I, \beta_O) = \begin{cases} m' \cdot \beta_O(P^{(\triangle)}_{mi \leftarrow ma}(p_{X'}, p_{Y'})) + g(i) \cdot \beta_O(P^{(\triangle)}_{g \leftarrow ma}(p_{X'}, p_{Y'})), & X'_i = Y'_i \\ m' \cdot \beta_O(P^{(\triangle)}_{mi \leftarrow mi}(p_{X'}, p_{Y'})) + g(i) \cdot \beta_O(P^{(\triangle)}_{g \leftarrow mi}(p_{X'}, p_{Y'})), & X'_i \neq Y'_i,\ X'_i, Y'_i \in \Sigma \\ g(i) \cdot \beta_O(P^{(\triangle)}_{g \leftarrow g}(p_{X'}, p_{Y'}, X'_i, Y'_i)), & \textit{otherwise} \end{cases}$$

for $p_{X'} = \beta_I(p(X'_i))$ and $p_{Y'} = \beta_I(p(Y'_i))$ the error probabilities of X'_i and Y'_i and $g(i) = g'_e + g'_o \cdot open(X', Y', i)$.

A.2 Model-supported methods

This section contains additional remarks on and pseudocode descriptions of the model-supported methods presented in Section "Model-supported methods". The following table relates the different model-supported clustering and refinement methods to their implementations in GeFaST:

Implementing class	Synopsis
`ConsistentClassicSwarmer`	Clustering by similarity and consistency (Algorithm S1)
`ConsistencySwarmer`	Clustering by consistency alone (Algorithm S2)
`LightSwarmAppender`	Attaching light swarms as a whole (Algorithm S4)
`LightSwarmRefiner`	Attaching light-swarm amplicons independently (Algorithm S5)
`LightSwarmShuffler`	Shuffling light-swarm amplicons (Algorithm S6)

Error-matrix computation. The transition probabilities in DADA2 are either provided by the user or can be estimated from the data. In GeFaST, the transition probabilities can also be specified by the user or a default error matrix based on the quality score is computed. The default matrix distinguishes only between match and mismatch and does not consider the participating nucleotides. Let a and b be the nucleotides of the two sequences participating in a substitution. Since the error model only considers the quality of the second sequence (Equation (1)), only the error probability $p(b)$ is used. The transition probability is $1 - p(b)$ for $a = b$ and $\frac{p(b)}{|\Sigma|-1}$ otherwise.

Bonferroni correction. In DADA2, the consistency of the current partitioning is checked by testing the abundance p-value of each amplicon. Thus, a large number of related hypothesis tests is performed, increasing the risk of observing a significant result by chance and, thus, falsely recognising the partitioning as inconsistent. Similarly, we apply the Bonferroni correction to avoid the multiple testing problem in GeFaST, because we also compare individuals against (possibly) many other subseeds (clustering) or heavy-swarm seeds (refinement).

A.2.1 Consistency-checked clustering

Both clusterers follow the iterative approach and depend on the consistency check described in Algorithm 2.

Algorithm S1: Consistency-checked, iterative clustering on one amplicon pool. The abundance comparison (see the comment above line 9) is only performed if the breaking mechanism is activated. Even though the consistency check itself involves an alignment, this model-supported clustering method can be used with any (binary) distance function d available in GeFaST. Implemented in the `ConsistentClassicSwarmer` clusterer.

Input: $\mathcal{A}$ = amplicon pool, t_c = clustering threshold, d = distance function, E = error matrix, Ω_A = abundance p-value threshold, N = number of all amplicons
Output: $\mathcal{C}$ = collection of clusters

1 $\mathcal{C} = \emptyset$;
2 **while** unswarmed amplicon in $\mathcal{A}$ **do**
3 Select most abundant unswarmed amplicon s from $\mathcal{A}$;
4 Mark s as swarmed;
5 Initialise cluster C and (sub)seed queue Q with seed s;
`/* amplicons in` Q `are sorted by generation (ascending) and within each generation by abundance (descending) */`
6 **while** $Q \neq \emptyset$ **do**
7 Remove next subseed s' from Q;
8 Find partners P of s' among unswarmed amplicons in $\mathcal{A}$;
`/*` p `is a partner if` $d(s', p) \leq t_c$ `(and` $ab(p) \leq ab(s')$`) */`
9 **for** $p \in P$ **do**
10 **if** IsConsistent(s', p, E, Ω_A, N) **then** `// see Algorithm 2`
11 Add amplicon p and edge (s', p) with weight $d(s', p)$ to C;
12 Insert p into Q;
13 Mark p as swarmed;
14 **end**
15 **end**
16 **end**
17 Add cluster C to $\mathcal{C}$;
18 **end**
19 **return** $\mathcal{C}$;

Algorithm S2: Consistency-based iterative clustering on one amplicon pool. The clustering is independent of a distance function. Implemented in the `ConsistencySwarmer` clusterer.

Input: $\mathcal{A}$ = amplicon pool, E = error matrix (transition probabilities), Ω_A = abundance p-value threshold, N = number of all amplicons

Output: $\mathcal{C}$ = collection of clusters

$\mathcal{C} = \emptyset$;
while unswarmed amplicon in $\mathcal{A}$ **do**
    Select most abundant unswarmed amplicon s from $\mathcal{A}$;
    Mark s as swarmed;
    Initialise cluster C and (sub)seed queue Q with seed s;
    `/* amplicons in` Q `are sorted by generation (ascending) and within each generation by abundance (descending) */`
    while $Q \neq \emptyset$ **do**
        Remove next subseed s' from Q;
        for each unswarmed amplicon p in $\mathcal{A}$ with $ab(s') \geq ab(p) > 1$ **do**
            if IsConsistent(s', p, E, Ω_A, N) **then** `// see Algorithm 2`
                Add amplicon p and edge (s', p) to C;
                Insert p into Q;
                Mark p as swarmed;
            end
        end
    end
    Add cluster C to $\mathcal{C}$;
end
return $\mathcal{C}$;

A.2.2 Consistency-guided cluster refinement

The light clusters remaining after the refinement can be processed in four ways (Algorithm S3). The first two options are straightforward and simply retain or discard all of them. The third option combines all light clusters into a single cluster, using the first amplicon (of the first light cluster) as the seed and attaching all other amplicons as direct children to it. The fourth and last option is the most involved one. It starts by collecting the amplicons of the light clusters and putting them into a single partition (with the most abundance amplicon as its centre). Then, it applies DADA2's split-and-shuffle strategy, basically running Algorithm 1 but returning the full partitions (instead of just the sample sequences).

Algorithm S3: Processing of light clusters that have not been grafted onto a heavy one.

ProcessLight($\mathcal{L}$, opt)
  Input: $\mathcal{L}$ = collection of light clusters, opt = processing option
  Output: $\mathcal{L}'$ = collection processed light clusters
  switch *opt* **do**
    case 1 **do** `// keep the clusters without further changes`
      $\mathcal{L}' = \mathcal{L}$;
    end
    case 2 **do** `// discard the clusters`
      $\mathcal{L}' = \emptyset$;
    end
    case 3 **do** `// combine the clusters`
      Unite all clusters in $\mathcal{L}$ into a single (star-shaped) cluster L;
      $\mathcal{L}' = \{L\}$;
    end
    case 4 **do** `// combine and repartition the clusters`
      Collect the members of all clusters in $\mathcal{L}$ in R;
      Partition R into $\mathcal{P}$ by applying DADA2's split-and-shuffle strategy;
      Transform each partition $P \in \mathcal{P}$ into a star-shaped cluster;
      $\mathcal{L}' = \mathcal{P}$;
    end
  end
  return $\mathcal{L}'$;
end

Algorithm S4: Consistency-guided cluster refinement as implemented in `LightSwarmAppender`. Light swarms are considered as units and are only attached as a whole, depending on the consistency of the seed. Unattached light swarms are processed according to the chosen option.

Input: $\mathcal{C}$ = collection of clusters from clustering phase, b = boundary threshold, N = number of all amplicons, E = error matrix (transition probabilities), Ω_A = abundance p-value threshold, opt = option for handling of light swarms not appended

Output: Collection of refined clusters

Split $\mathcal{C}$ into $\mathcal{C}_{<b}$ and $\mathcal{C}_{\geq b}$; `// light resp. heavy clusters, Equation (S18)`
$\mathcal{L} = \emptyset$; `// ungrafted light clusters`
for each $L \in \mathcal{C}_{<b}$ **do**
  $max_{exp} = 0$; `// highest expected abundance`
  $max_{cl} = \bot$; `// heavy cluster yielding` max_{exp}
  $max_{p_A} = 0$; `// abundance` p`-value w.r.t.` max_{cl}
  for each $H \in \mathcal{C}_{\geq b}$ **do**
    `/* Let` ℓ `and` h `be the seeds of` L `and` H`, respectively. */`
    Align ℓ and h to determine the set of substitutions S;
    Calculate $\lambda_{\ell,h}$ from S and E; `// Equation (1)`
    if $\lambda_{\ell,h} \cdot ab(h) > max_{exp}$ **then**
      $max_{exp} = \lambda_{\ell,h} \cdot ab(h)$;
      $max_{cl} = H$;
      $max_{p_A} = p_A(ab(\ell), \lambda_{\ell,h} \cdot mass(H))$; `// Equation (2)`
    end
  end
  Compute Bonferroni-corrected abundance p-value $p'_A = max_{p_A} \cdot N$;
  if $max_{cl} \neq \bot \wedge p'_A \geq \Omega_A$ **then**
    Attach L to H;
  else
    Add L to $\mathcal{L}$;
  end
end
$\mathcal{L}$ = ProcessLight($\mathcal{L}$, opt); `// see Algorithm S3`
return $\mathcal{C}_{\geq b} \cup \mathcal{L}$;

Algorithm S5: Consistency-guided cluster refinement as implemented in `LightSwarmResolver`. Light swarms are disassembled and the obtained amplicons are attached individually. Unattached light swarms are processed according to the chosen option.

Input: $\mathcal{C}$ = collection of clusters from clustering phase, b = boundary threshold, N = number of all amplicons, E = error matrix (transition probabilities), Ω_A = abundance p-value threshold, opt = option for handling of light swarms not appended

Output: Collection of refined clusters

Split $\mathcal{C}$ into $\mathcal{C}_{<b}$ and $\mathcal{C}_{\geq b}$; `// light resp. heavy clusters, Equation (S18)`
$\mathcal{L} = \emptyset$; `// ungrafted light clusters`
for each $L \in \mathcal{C}_{<b}$ **do**
  for each *amplicon a in L* **do**
    $max_{exp} = 0$; `// highest expected abundance`
    $max_{cl} = \perp$; `// heavy cluster yielding` max_{exp}
    $max_{p_A} = 0$; `// abundance` p`-value w.r.t.` max_{cl}
    for each $H \in \mathcal{C}_{\geq b}$ **do**
      `/* Let` h `be the seed of` H`. */`
      Align a and h to determine the set of substitutions S;
      Calculate $\lambda_{a,h}$ from S and E; `// Equation (1)`
      if $\lambda_{a,h} \cdot ab(h) > max_{exp}$ **then**
        $max_{exp} = \lambda_{a,h} \cdot ab(h)$;
        $max_{cl} = H$;
        $max_{p_A} = p_A(ab(a), \lambda_{a,h} \cdot mass(H))$; `// Equation (2)`
      end
    end
    Compute Bonferroni-corrected abundance p-value $p'_A = max_{p_A} \cdot N$;
    if $max_{cl} \neq \perp \wedge p'_A \geq \Omega_A$ **then**
      Graft a onto H;
    else
      Initialise new cluster with a and add it to $\mathcal{L}$;
    end
  end
end
$\mathcal{L}$ = PROCESSLIGHT($\mathcal{L}$, opt); `// see Algorithm S3`
return $\mathcal{C}_{\geq b} \cup \mathcal{L}$;

Algorithm S6: Consistency-guided cluster refinement as implemented in `LightSwarmShuffler`. The amplicons from a light cluster are indivually reassigned to heavy clusters. A seed can only be attached if it is the last remaining member of its cluster. The remaining members of a light cluster are rearranged into a star. Unattached light clusters are not processed afterwards.

Input: $\mathcal{C}$ = collection of clusters from clustering phase, b = boundary threshold, N = number of all amplicons, E = error matrix (transition probabilities), Ω_A = abundance p-value threshold

Output: Collection of refined clusters

Split $\mathcal{C}$ into $\mathcal{C}_{<b}$ and $\mathcal{C}_{\geq b}$; `// light resp. heavy clusters, Equation (S18)`

$\mathcal{L} = \emptyset$; `// ungrafted light clusters`

for each $L \in \mathcal{C}_{<b}$ **do**

  $\mathcal{A} = \emptyset$; `// ungrafted amplicons of` L

  `/* Below iteration over the amplicons proceeds in reverse, breadth-first order, i.e. the seed is processed last. */`

  for each *amplicon a in L* **do**

    $max_{exp} = 0$; `// highest expected abundance`

    $max_{cl} = \perp$; `// heavy cluster yielding` max_{exp}

    $max_{p_A} = 0$; `// abundance` p`-value w.r.t.` max_{cl}

    for each $H \in \mathcal{C}_{\geq b}$ **do**

      `/* Let` h `be the seed of` H`. */`

      Align a and h to determine the set of substitutions S;

      Calculate $\lambda_{a,h}$ from S and E; `// Equation (1)`

      if $\lambda_{a,h} \cdot ab(h) > max_{exp}$ **then**

        $max_{exp} = \lambda_{a,h} \cdot ab(h)$;

        $max_{cl} = H$;

        $max_{p_A} = p_A(ab(a), \lambda_{a,h} \cdot mass(H))$; `// Equation (2)`

      end

    end

    Compute Bonferroni-corrected abundance p-value $p'_A = max_{p_A} \cdot N$;

    if $a \neq seed(L) \vee \mathcal{A} = \emptyset$ **then**

      if $max_{cl} \neq \perp \wedge p'_A \geq \Omega_A$ **then**

        Graft a onto H;

      else

        Add a to $\mathcal{A}$;

      end

    end

  end

  Initialise new cluster from $\mathcal{A}$ and add it to $\mathcal{L}$;

end

return $\mathcal{C}_{\geq b} \cup \mathcal{L}$;

B GeFaST clustering framework

Supplement B contains an extended description of the workflow of GeFaST and the involved components. Furthermore, information on how to configure and execute GeFaST are provided.

B.1 A deeper look at the workflow

Configuring the execution of GeFaST. One of the mandatory arguments of GeFaST is the mode, based on which the appropriate `Configuration` implementation is chosen:

Mode	Abbreviation	Configuration
Levenshtein	`lev`	`LevenshteinConfiguration`
Alignment score	`as`	`AlignmentScoreConfiguration`
Quality Levenshtein	`qlev`	`QualityLevenshteinConfiguration`
Quality alignment score	`qas`	`QualityAlignmentScoreConfiguration`
Consistency	`cons`	`ConsistencyConfiguration`

Next, the configuration is filled with default values (if applicable) and the parameters read from the configuration file. Afterwards, the other command-line parameters (besides the mode abbreviation) are considered, adding or overwriting information in the configuration.

All the implementations of `Configuration` share a basic set of general parameters needed for properly running GeFaST, e.g. the clustering threshold, the output file(s) and filtering options for the preprocessors. In addition, the different modes can specify additional mode-specific parameters. Those parameters, e.g. the selected quality-weighted cost function and boosting function for the quality Levenshtein mode, are provided via the configuration file.

Preprocessing and amplicon representation. Implementations of `Preprocessor` are responsible for reading sequence information from one or more input files, preprocessing the sequences according to the configuration and inserting them in an amplicon storage. An amplicon storage consists of one more amplicon pools (each represented by an `AmpliconCollection`), if possible separating groups of amplicons between which there can be no links based on the clustering (and refinement) threshold. While preprocessors extract the available information from the input files, they do not determine which pieces of information are accessible during the later phases. The used `AmpliconStorage` implementation decides on the kept information. For example, when GeFaST is run in a quality-unaware mode but the sequences are provided in FASTQ format, the FASTQ preprocessor also reads the quality scores and hands them over to the amplicon storage but they might be discarded there since they are not needed in the clustering and refinement phases.

Preprocessors are also responsible for formatting and, optionally, filtering the input. All sequences are normalised to upper-case letters during the preprocessing and, by default, only sequences from the alphabet $\Sigma = \{A, C, G, T\}$ are kept. Furthermore, minimum and maximum sequence length and abundance values can be specified. The filtering is strict in a sense that a sequence is dropped completely if it does not meet a filter criterion (instead of, e.g., just deleting non-alphabet letter, trimming the length or reducing the abundance value).

Currently, GeFaST can handle files in FASTA and FASTQ format but for a single run of GeFaST all files must have the same format. The `FastqPreprocessor` accepts only single-line (sequential) sequence files, while the `FastaPreprocessor` can also handle multi-line (interleaved) files. By default, the `FastqPreprocessor` assumes FASTQ files to be in Sanger format but also supports the Illumina 1.3+, Illumina 1.5+ and Illumina 1.8+ format. Moreover, the `FastqPreprocessor` dereplicates the sequences and, by default, averages the quality scores of the same sequences (per position). Both preprocessors finish off the preprocessing by sorting the amplicons within each pool by their abundance in decreasing order.

Clustering phase. The mandatory clustering phase is the centrepiece of GeFaST as it performs the primary partitioning of the given amplicons into clusters. This phase is executed by `Clusterer` implementations and strongly depends on the configuration, which also provides the key parameters such as the clustering threshold t_c. Below, we describe the original clustering method of GeFaST, now generalised to arbitrary distance functions.

The ClassicSwarmer implements the iterative clustering strategy of Swarm. However, it is not specific to a particular distance function (such as score-based Levenshtein distance). Instead, it is an abstract implementation into which any distance function (once implemented in GeFaST) can be incorporated. The distance calculation – as part of the search for similar sequences (also referred to as partners) – is encapsulated in methods provided by an auxiliary data structure. These distance function-specific data structures (of type AuxiliaryData) also allow the use of further supporting data structures, e.g. segment filters to improve the runtime when using the edit distance [8]. The auxiliary data structures are built per amplicon pool with the help of the configuration. Algorithm S7 describes the operations of ClassicSwarmer on one amplicon pool. ClassicSwarmer also implements the non-parameterised breaking mechanism used in newer Swarm versions.

Algorithm S7: Iterative clustering on one amplicon pool as implemented in GeFaST's ClassicSwarmer clusterer. The abundance comparison (see the comment above line 9) is only performed if the breaking mechanism is activated.

Input: $\mathcal{A}$ = amplicon pool, t_c = clustering threshold, d = distance function
Output: $\mathcal{C}$ = collection of clusters
1 $\mathcal{C} = \emptyset$;
2 **while** unswarmed amplicon in $\mathcal{A}$ **do**
3   Select most abundant unswarmed amplicon s from $\mathcal{A}$;
4   Mark s as swarmed;
5   Initialise cluster C and (sub)seed queue Q with seed s;
  /* amplicons in Q are sorted by generation (ascending) and within each generation by abundance (descending) */
6   **while** $Q \neq \emptyset$ **do**
7   Remove next subseed s' from Q;
8   Find partners P of s' among unswarmed amplicons in $\mathcal{A}$;
    /* p is a partner if $d(s', p) \leq t_c$ (and $ab(p) \leq ab(s')$) */
9   **for** $p \in P$ **do**
10   Add amplicon p and edge (s', p) with weight $d(s', p)$ to C;
11   Insert p into Q;
12   Mark p as swarmed;
13   **end**
14   **end**
15   Add cluster C to $\mathcal{C}$;
16 **end**
17 **return** $\mathcal{C}$;

Similar to the definition for Swarm, the partners of an amplicon a with respect to distance function d and clustering threshold t_c can be defined as

$$P_d(a, \mathcal{A}', t_c, br) = \left\{ b \in \mathcal{A}' \mid d(a, b) \leq t_c \wedge (br \Rightarrow ab(a) \geq ab(b)) \right\},$$

with $\mathcal{A}' \subseteq \mathcal{A}$ the unswarmed amplicons in $\mathcal{A}$ and br a Boolean expression indicating whether the breaking mechanism is activated.

Swarm representation. Each individual cluster is represented by a Swarm instance, allowing access to the members and overall properties such as its mass or number of generations. Similar to amplicons, the clusters coming from one pool are stored together (in a Swarms instance). Overall, the clusters (or swarms) from the different pools are managed in a SwarmStorage, which is then used in the refinement and output phases.

Refinement phase. The optional refinement phase aims for improving the quality of the clusters determined in the previous clustering phase. The refinement is controlled by implementations of ClusterRefiner and works on the provided Swarms instance. Similar to the clustering phase, the chosen implementation and key parameters such as the refinement threshold t_r and the boundary threshold b are obtained from the configuration. In the following, we describe the generalised fastidious refinement method implemented in GeFaST, now also being independent from a specific distance function.

The FastidiousRefiner generalises Swarm's fastidious cluster refinement strategy in two ways, while keeping the idea of grafting light clusters onto heavy ones. First, it is no longer restricted to clustering threshold

$t_c = 1$. Second, the refinement threshold t_r is now freely adjustable and independent of t_c (instead of being twice the clustering threshold). This allows more or less conservative fastidious refinement as needed.

The distance calculation is again encapsulated in auxiliary data structures built per amplicon pool with the help of the configuration. For the fastidious refinement, the auxiliary data structures are also responsible for ensuring that partners are only found between light and heavy clusters.
In contrast to the clustering phase, finding partners does not result in the immediate generation of new links between amplicons. The search rather results in a collection of potential grafting links, which are prioritised and subject to certain restrictions detailed below. Once the so-called valid grafting links have been determined, the actual grafting is performed.

Subsequently, we provide a more formal description of the fastidious refinement process. As in Swarm, we distinguish between light and heavy clusters based on their mass. For a collection of clusters $\mathcal{C}$ and boundary threshold b, we define the respective groups of clusters as follows:

$$\mathcal{C}_{<b} = \left\{ (V, E, s, w) \in \mathcal{C} \;\middle|\; \sum_{a \in V} ab(a) < b \right\}, \quad \mathcal{C}_{\geq b} = \left\{ (V, E, s, w) \in \mathcal{C} \;\middle|\; \sum_{a \in V} ab(a) \geq b \right\} \tag{S18}$$

A *grafting link* can only be established between an amplicon from a light cluster and another one from a heavy cluster. Let

$$L_b(\mathcal{C}) = \bigcup_{(V,E,s,w) \in \mathcal{C}_{<b}} V, \quad H_b(\mathcal{C}) = \bigcup_{(V,E,s,w) \in \mathcal{C}_{\geq b}} V$$

be the sets of amplicons from all light and heavy clusters, respectively. The set of *potential grafting links* with respect to distance function d and threshold t is then defined as

$$\mathcal{L}_d(\mathcal{C}, t, b) = \{(h, l) \mid h \in H_b(\mathcal{C}) \wedge l \in L_b(\mathcal{C}) \wedge d(h, l) \leq t\}.$$

For an amplicon $l \in L_b(\mathcal{C})$, there can be multiple potential grafting partners $h \in H_b(\mathcal{C})$, but only the one with the highest abundance is actually considered during the grafting process. Furthermore, a light cluster is grafted at most once, even if there are potential grafting links to several heavy clusters. Hereinafter, we assume that $\mathcal{L}_d(\mathcal{C}, t, b)$ is sorted such that for all (h_i, l_i) and (h_j, l_j) with $i < j$

$$ab(h_i) > ab(h_j) \vee (ab(h_i) = ab(h_j) \wedge ab(l_i) > ab(l_j)).$$

holds. Finally, the *valid grafting links*, which are used in the actual grafting step (Algorithm S8), are defined as

$$\mathcal{V}_d(\mathcal{C}, t, b) = \left\{(h_i, l_i) \in \mathcal{L}_d(\mathcal{C}, t, b) \;\middle|\; \neg\exists (h_j, l_j) \in \mathcal{L}_d(\mathcal{C}, t, b).(j < i \wedge cl(l_i) = cl(l_j))\right\}$$

where $cl(a)$ denotes the cluster containing amplicon a.

Algorithm S8: Fastidious cluster refinement in GeFaST

Input: $\mathcal{C}$ = collection of clusters from clustering phase, t_r = refinement threshold, b = boundary threshold, d = distance function
Output: $\mathcal{C}$ = collection of refined clusters

```
Determine V_d(C, t_r, b);
for (h, l) ∈ V_d(C, t_r, b) do
    Let (V_h, E_h, s_h, w_h) = cl(h) and (V_l, E_l, s_l, w_l) = cl(l);
    V_h = V_h ∪ V_l;
    E_h = E_h ∪ E_l ∪ {(h, l)};
    w_h = w_h ∪ w_l ∪ {(h, l) ↦ d(h, l)};
    C = C \ {cl(l)};
end
return C;
```

Output generation. `OutputGenerator` implementations manage the final phase of GeFaST's workflow: creating output files describing the clusters determined during the clustering and refinement phases. Together with the configuration, an output generator defines the produced output files and their format. Currently, there is only one general output generator (`ClassicOutputGenerator`), mimicking the output of Swarm (v2) and providing up to four main output files. The *OTU output* lists the members of all clusters, using one line per cluster, while the links between the amplicons are described in the *internal-structures output*. For each link, details such as the distance between the participating amplicons and the generation of the child amplicon are stated. Additional information on the clusters, such as their size, mass and number of generations, is provided in the *statistics output*. The *seeds output* contains each OTU seed in FASTA format, using the mass of its cluster as the abundance value.

B.2 Usage of GeFaST

GeFaST has been developed for Linux systems and offers a simple command-line interface. The most basic usage of GeFaST involves just three mandatory parameters:

```
GeFaST <mode> <input> <configuration file>
```

The first required parameter is the abbreviation of the selected mode (e.g. `lev`), while the second paramter specifies the input files as a comma-separated list of file paths. The third mandatory parameter is the file path to the configuration file.

Configuration and command-line parameter syntax. The configuration file is a (possibly empty) plain-text file containing key-value pairs. Each configuration parameter is written in its own line and has the form `<key>=<value>`. Empty lines and comment lines (starting with #) are allowed. An exemplary configuration could look like the following:

```
# basic configuration
threshold=3
output_otus=otus.txt
use_score=1
```

Based on this configuration file, GeFaST would use a clustering threshold of three and create the specified OTU output file. The last entry, `use_score=1`, is a parameter specific to the Levenshtein mode and would activate the score-based Levenshtein distance. Other modes would simply ignore this parameter.
The basic configuration is then extended by or (partly) overwritten by the command-line parameters. This allows to comfortably reuse large configurations and to add or adapt the more variable parameters via the command line. Most command-line parameters have a short and a long form and expect a value (e.g. both `-o` and `-output_otus` can be used to specify the OTU output file). Other parameters do not have a short form (usually less common ones) or do not expect a value, but the order of the parameters can be chosen freely.

As an example, consider the command

```
GeFaST lev data/amplicons.fasta run.conf -t 2 -w seeds.fasta
```

together with above configuration file (stored as `run.conf`). Here, GeFaST runs in Levenshtein mode (still using the score-based Levenshtein distance) and reads amplicons from a single input file (`data/amplicons.fasta`). However, GeFaST now uses a clustering threshold of two (for this parameter, the initial configuration read from `run.conf` is overwritten) and outputs the cluster seeds in FASTA format in addition to the OTU output file.

GeFaST also offers a help function that can be called by `GeFaST -h` or `GeFaST -help`. More documentation, including a full list of the (mode-specific) parameters, can be found in GeFaST's manual, which is available at `https://github.com/romueller/gefast`.

Compatibility of modes and components. The new modular structure of GeFaST allows to reuse major components in multiple modes. Nevertheless, there are also some restrictions, usually to separate the notions of distance embodied by different modes. While all modes are compatible with the `FastqPreprocessor`, only the Levenshtein and the alignment-score mode can also use the `FastaPreprocessor` as they do not require quality information. In addition, all modes work with the `ClassicOutputGenerator`. The following table shows the compatibility of the modes with the available clustering and refinement methods.

<table>
<tr><th>Mode</th><th>Distance function(s)</th><th>Clusterers</th><th>Cluster refiners</th></tr>
<tr><td>as</td><td>BoundedScoreDistance,
BoundedBandedScoreDistance</td><td rowspan="4">ClassicSwarmer,
Consistent-
ClassicSwarmer</td><td rowspan="4">IdleRefiner,
FastidiousRefiner,
LightSwarmAppender,
LightSwarmRefiner,
LightSwarmShuffler</td></tr>
<tr><td>lev</td><td>BoundedLevenshteinDistance,
BoundedScoreLevenshteinDistance</td></tr>
<tr><td>qas</td><td>BoundedQualityAlignmentScore,
BoundedBandedQualityAlignmentScore</td></tr>
<tr><td>qlev</td><td>BoundedQualityLevenshteinDistance</td></tr>
<tr><td>cons</td><td>—</td><td>ConsistencySwarmer</td><td>IdleRefiner,
LightSwarmAppender,
LightSwarmResolver,
LightSwarmShuffler</td></tr>
</table>

Above table also indicates the distance functions that can be used with the different modes. All the distance functions are *bounded*, i.e. they do not necessarily compute the actual distance between two amplicons. The underlying alignment computations are equipped with early-termination checks and stop the computation as soon as it is detected that the distance between the amplicons is definitely larger than the distance threshold. In this case, the threshold plus one is returned instead of the actual distance. Most of the distance functions (in fact, all except `BoundedQualityAlignmentScore`) are also *banded*. The corresponding alignment computations are sped up by restricting the computation to a (small) number of bands on each side of the main diagonal of the dynamic-programming matrix. Usually, the number of bands necessary to avoid influencing the result of the computation can be determined automatically from the distance threshold and operation costs. `BoundedBandedQualityAlignmentScore`, however, can have minute operation costs contributing to the alignment score (and, thus, distance), which would effectively lead to the unbanded version. In order to have a similar speed up, the number of bands is therefore computed from the unweighted scoring function as for the other distance functions (or is specified by the user). While this could possibly exclude an optimal alignment from the computation, preliminary evaluations (not shown here) have indicated a marginal impact.

C Evaluation

This supplement contains detailed information on the evaluation data sets (Section C.1) and a more comprehensive analysis of the evaluation results (Sections C.2 and C.3).

C.1 Data sets

This section provides additional information on the different mock-community data sets used in our evaluation, including a description of how to obain and prepare the reads. The exact commands are available in the evaluation repository.

C.1.1 From synthetic sequencing

The first group of data sets comprises amplicons obtained from synthetic sequencing as described by Franzén et al. [9]. The underlying mock communities were derived from reference 16S rRNA sequences selected from the Greengenes database [10] (v.13.5.99), restricted to the phylum Bacteroidetes (one of the dominant phyla with respect to mammalian microbiotas) and sequences at least 1400 bp long. In addition, genera with more than 1000 sequences were downsampled to 100 sequences. The mock communities were randomly selected at three levels of complexity: low (`LC`, containing 100 references), medium (`MC`, 250) and high (`HC`, 500). At each level, 10 mock communities were generated. The composition of the different mock communities is listed in the supplement of Franzén et al. (Additional file 9).

In preparation of the in silico sequencing with the read simulator ART [11], a hand-curated 16S rRNA multiple sequence alignment (`bacteria16S_508_mod5.stk`) was downloaded via `http://rdp.cme.msu.edu/download/RDPinfernalTraindata.zip` and, subsequently, formatted with the help of Infernal [12] (v1.1.2, command `cmbuild --ere 1.4`). Next, two data sets covering the V3-V4 and V4 region, respectively, were created from each mock community as follows:

1) **Obtaining the mock-community references**
   Extract the sequences of the mock community from the Greengenes database (release 13_5) and remove any ambiguous nucleotides (such as `N`) from them.

2) **Identification of the hypervariable regions in the references**
   Align the extracted sequences to the 16S rRNA multiple sequence alignment using Infernal (command `cmalign` with default parameters).

3) **Extracting the regions to be sequenced from the references**
   Extract subsequences of the aligned sequences corresponding to the V3-V4 respectively V4 region.
   - V3-V4: positions 227 to 801
   - V4: positions 389 to 801

4) **Synthetic sequencing**
   Simulate MiSeq paired-end sequencing with ART (VanillaIceCream release).
   - V3-V4 (2 x 250 bp reads): `art_illumina -amp -na -p -l 250 -f 100`
   - V4 (2 x 150 bp reads): `art_illumina -amp -na -p -l 150 -f 100`

5) **Generating the amplicon data sets**
   For each set of paired-end reads, the reverse complement of the second read in each pair is concatenated with the first read (in contrast to Franzén et al. without spacer `N`s). The concatenated reads are then subsampled to 20 reads per reference and shuffled.

6) **Obtaining the ground truth**
   For both amplicon data sets, the taxonomy file in blast6out format used as the ground truth is generated from the amplicon identifiers, which contain the identifier of the mock-community reference. Since we only need the first two columns (query and target label) of the blast6out format in our evaluations, the remaining values are filled with placeholders.

The ground truths of the synthetic data sets can be considered perfect because the source of each read can be traced back and, thus, each read could be assigned to the reference it originated from. Below table shows the number of reads, their average length and average quality score for each data set:

Community	Number of reads		Average length		Average quality score	
	V3-V4	V4	V3-V4	V4	V3-V4	V4
LC_1	1980	1980	500.0	300.0	33.88	35.38
LC_2	1980	1980	500.0	300.0	33.86	35.37
LC_3	2000	2000	500.0	300.0	33.87	35.38
LC_4	2000	2000	500.0	300.0	33.86	35.40
LC_5	1980	1980	500.0	300.0	33.89	35.37
LC_6	2000	2000	500.0	300.0	33.88	35.38
LC_7	2000	2000	500.0	300.0	33.87	35.37
LC_8	1980	1980	500.0	300.0	33.87	35.36
LC_9	1980	1980	500.0	300.0	33.89	35.39
LC_10	1980	1980	500.0	300.0	33.87	35.38
MC_1	4960	4960	500.0	300.0	33.88	35.37
MC_2	4940	4940	500.0	300.0	33.88	35.39
MC_3	4980	4980	500.0	300.0	33.88	35.38
MC_4	4980	4980	500.0	300.0	33.88	35.38
MC_5	4940	4940	500.0	300.0	33.87	35.38
MC_6	4960	4960	500.0	300.0	33.87	35.38
MC_7	4960	4960	500.0	300.0	33.88	35.38
MC_8	4980	4980	500.0	300.0	33.88	35.38
MC_9	4980	4980	500.0	300.0	33.88	35.38
MC_10	4980	4980	500.0	300.0	33.88	35.38
HC_1	9940	9940	500.0	300.0	33.88	35.38
HC_2	9960	9960	500.0	300.0	33.88	35.38
HC_3	9960	9960	500.0	300.0	33.88	35.37
HC_4	9980	9980	500.0	300.0	33.88	35.38
HC_5	9980	9980	500.0	300.0	33.87	35.38
HC_6	9960	9960	500.0	300.0	33.88	35.38
HC_7	9940	9940	500.0	300.0	33.87	35.38
HC_8	9980	9980	500.0	300.0	33.88	35.38
HC_9	9940	9940	500.0	300.0	33.88	35.38
HC_10	9940	9940	500.0	300.0	33.88	35.38

Please note that the number of reads in the data sets did not necessarily correspond to the expected number (i.e. the number of references multiplied by 20). The slight deviations apparently stem from minor differences in the versions of the used release of the Greengenes database. Consequently, a few of the listed references could not be found during the construction of the data sets.

C.1.2 From non-synthetic sequencing

We further evaluated our clustering methods on mock-community data sets containing amplicons actually sequenced with the Illumina MiSeq platform. The data sets were obtained and prepared for our evaluations as described by Callahan et al. [13].

Balanced mock community. The `balanced` mock community comprises 59 bacterial and archaeal organisms from various habitats at nominally equal frequencies (see Table S6 in the supplement of Schirmer et al. [14] for details of the composition). The paired reads were obtained from sequencing the V4 region of the 16S rRNA gene using the Illumina MiSeq platform with read length 2 x 250 bp. The forward reads alone respectively the paired reads were then used to obtain two data sets for our evaluations as follows:

1) **Obtaining the read files**
According to Callahan et al., the data set is named *DS 35* in the work of Schirmer et al. [14] and is available on the European Nucleotide Archive under the study accession number PRJEB6244. However, the accession numbers of the data sets provided in Table S5 in the supplement to Schirmer et al. do not fit the ones provided for the study on ENA. Table 1 in the supplement of Callahan et al. states that the data set contains 593,868 forward resp. reverse reads and the only run with the appropriate number of

reads is ERR777695.
The forward and reverse reads were then downloaded via:
`ftp://ftp.sra.ebi.ac.uk/vol1/fastq/ERR777/ERR777695/ERR777695_1.fastq.gz`
`ftp://ftp.sra.ebi.ac.uk/vol1/fastq/ERR777/ERR777695/ERR777695_2.fastq.gz`

2) **Filtering**
Subsequently, the reads for the `single` (forward only) and `paired` (forward and reverse) data sets were filtered using DADA2 along the lines of the description from Callahan et al.:

   - `single`: `fastqFilter` with $maxN = 0$, $maxEE = 2$, $truncQ = 2$, $truncLen = 240$, $trimLeft = 20$
   - `paired`: `fastqPairedFilter` with $maxN = 0$, $maxEE = 2$, $truncQ = 2$, $truncLen = c(240, 220)$, $trimLeft = (20, 20)$

3) **Merging**
The filtered reads of the `paired` data set were then merged using USEARCH's `fastq_mergepairs` command with `-fastq_minovlen 20` and `-fastq_maxdiffs 1`.

4) **Dereplication and change of abundance separator**
The filtered (and merged) reads were then dereplicated using USEARCH's `derep_fulllength` command with `-sizeout` and `-minuniquesize 1`. For an easier use with GeFaST, we also changed the abundance separator from `;size=` to an underscore using the `sed` command.

5) **Obtaining the ground truth**
The associated reference sequences were obtained from the Stanford Digital Repository entry for Callahan et al. (https://purl.stanford.edu/mh194vj6733), downloading `Balanced_Data.zip` and extracting reference file `BalancedRefSeqs.fasta`. The reference file did not require further preprocessing. The taxonomic assignment of the reads was obtained by matching them against the reference sequences. To this end, we used VSEARCH's `usearch_global` command with an identity threshold (`--id`) of 0.97 and the `--blast6out` output option.

In contrast to the synthetic data sets, the ground truths are not complete because only 85.9 % of the amplicons in the `single` data set and 85.5 % of the amplicons in the `paired` data set could be assigned to one of the provided reference sequences. The majority of the unassigned sequences in both data sets is presumably chimeric. For example, using VSEARCH's `uchime_ref` command and the reference sequences accompanying the `balanced` mock community, 74.5 % (55.1 %) of the unassigned sequence in the `paired` (`single`) data set were identified as chimeras. Both chimeric and non-chimeric unassigned sequences had a much lower average abundance than the assigned sequences, possibly hinting at a larger share of sequencing artefacts. Among the unassigned sequences, the average abundance of the non-chimeric ones tended to be higher. The more abundant sequences in that group might be proper reads which fell short of the identity threshold used for obtaining the ground truth (but lowering that threshold would also assign more chimeric sequences). We also inspected exemplary clusterings obtained with different tools and found that the unassigned sequences had a strong tendency to cluster together separately from the assigned sequences in smaller clusters.
The following table describes the development of the number of reads, the average read length and the average quality score during the preparation steps. The rows printed in italics correspond the data sets used in our evaluations.

	Data set	Number of reads	Average length	Average quality score
`single`	forward (raw)	593868	250.0	35.9
	forward (filtered)	558059	220.0	36.7
	forward (dereplicated)	*33523*	*220.0*	*36.8*
`paired`	forward (raw)	593868	250.0	35.9
	reverse (raw)	593868	250.0	33.5
	forward (filtered)	493046	220.0	36.9
	reverse (filtered)	493046	200.0	36.7
	merged	467652	250.9	40.1
	merged (dereplicated)	*21808*	*250.8*	*40.4*

HMP mock community. The HMP mock community contains 21 bacteria at nominally equal frequences [15]. The paired reads in the data set were obtained from sequencing the hypervariable regions V3-V4, V4 and V4-V5 of the 16S rRNA gene using the Illumina MiSeq platform with read length 2 x 250 bp. As before, the forward reads alone respectively the paired reads were used to obtain two data sets for our evaluations:

1) **Obtaining the read files**
As described in Callahan et al., we downloaded the data collection of run 130403 via `https://www.mothur.org/MiSeqDevelopmentData/130403.tar`. Subsequently, we extracted the files containing the forward and reverse reads of the *Mock1* community from the archive (`Mock1_S1_L001_R1_001.fastq.bz2` and `Mock1_S1_L001_R2_001.fastq.bz2`).

2) **Filtering**
Subsequently, the reads for the `single` (forward only) and `paired` (forward and reverse) data sets were filtered using DADA2 along the lines of the description from Callahan et al.:

- `single`: `fastqFilter` with $maxN = 0$, $maxEE = 2$, $truncQ = 2$, $truncLen = 240$, $trimLeft = 20$
- `paired`: `fastqPairedFilter` with $maxN = 0$, $maxEE = 2$, $truncQ = 2$, $truncLen = c(240, 200)$, $trimLeft = (20, 20)$

3) **Merging**
The filtered reads of the `paired` data set were then merged using USEARCH's `fastq_mergepairs` command with `-fastq_minovlen 20` and `-fastq_maxdiffs 1`.

4) **Dereplication and change of abundance separator**
The filtered (and merged) reads were then dereplicated using USEARCH's `derep_fulllength` command with `-sizeout` and `-minuniquesize 1`. For an easier use with GeFaST, we also changed the abundance separator from `;size=` to an underscore using the `sed` command.

5) **Obtaining the ground truth**
The associated reference sequences were also obtained from the same Stanford Digital Repository entry for Callahan et al., this time downloading `HMP_Data.zip` and extracting file `HMP_MOCK.fasta`. In contrast to the reference file for the `balanced` mock community, entries in the file belonging to the same species are numbered consecutively (e.g. A.baumannii.1 and A.baumannii.2) and, thus, would be considered as different in the evaluations. Therefore, we removed the numbering from the identifiers. The taxonomic assignment of the reads was obtained by matching them against the reference sequences. To this end, we used VSEARCH's `usearch_global` command with an identity threshold (`--id`) of 0.97 and the `--blast6out` output option.

As with the `balanced` mock-community data sets, the ground truths are not complete. Only 84.5 % of the amplicons in the `single` data set and 79.4 % of the amplicons in the `paired` data set could be assigned to one of the references. Again, large portions of the `hmp` data sets can be considered chimeric. VSEARCH's `uchime_ref` command identified 86.9 % (42.8 %) of unassigned sequences in the `paired` (`single`) data set as chimeras when using the reference sequences of the `hmp` mock community. Other observed differences between assigned and unassigned sequences were very similar to the ones described for the `balanced` data sets.
The table below again provides information on the number of reads, the average read length and the average quality score, with the rows printed in italics corresponding to the data sets used in our evaluations.

	Data set	Number of reads	Average length	Average quality score
`single`	forward (raw)	613352	250.9	32.3
	forward (filtered)	449409	220.0	34.9
	forward (dereplicated)	*73071*	*220.0*	*33.6*
`paired`	forward (raw)	613352	250.9	32.3
	reverse (raw)	613352	250.3	28.7
	forward (filtered)	303363	220.0	35.3
	reverse (filtered)	303363	180.0	33.9
	merged	208157	244.7	39.1
	merged (dereplicated)	*19882*	*296.5*	*37.9*

Differences in the data handling for DADA2. The recommended workflow of Callahan et al. for DADA2 merges the reads after running DADA2 on them, while the merge happens first for the examined clustering tools. Therefore, the workflow of DADA2 on the `balanced` and `hmp` data sets deviated from the above descriptions after the second step. Using the filtered reads, the remaining steps were replaced by functions of DADA2, involving dereplication, running the main function to determine the sample composition, chimera detection and, in case of `paired` data, merging.

C.2 Quality-weighted alignments

In this section, we elaborate on the main results described in Section "Clustering based on quality-weighted alignments" by providing additional details on how the different quality-weighted cost functions affected the clustering quality and performance for both the quality alignment-score and quality Levenshtein mode. The full analyses with additional plots are available as Jupyter notebooks in the evaluation repository.

Since we analysed the cost functions in a variety of configurations, we refer to the different boosted variants by using short descriptors, containing information on the boosting function (and its parameter), the boosting type and whether matches are part of the quality-weighting process. The boosting type indicates whether the boosting function is used as the inner or outer boosting function (while the other is set to the identity function *unboosted*). Variants with weighted matches use the original definitions of the cost functions, while those with unweighted matches apply the modified functions (denoted as $\widehat{w}$, see Section A.1). The different combinations of boosting type and (un)weighted matches are abbreviated as *v1* to *v4* as follows:

Abbreviation	Boosting type	Matches
v1	inner	weighted
v2	inner	unweighted
v3	outer	weighted
v4	outer	unweighted

For example, the descriptor *v2 / mult / 15* describes the boosted variant that uses the multiplicative boosting function with boosting parameter (factor) 15 as the inner boosting function β_I and that does not weight observed matches by the quality scores. The two unboosted variants (with weighted or unweighted matches) use *unboosted* for both the inner and the outer boosting function and are sometimes referred to as *mw* and *muw*, respectively.

C.2.1 Quality alignment-score mode

The maximum, average and N-best average clustering quality of the GeFaST in quality alignment-score mode, using the best variants for the different cost functions, are depicted in Figures S1, S2 and S3, respectively. The remarks on each quality-weighted cost function are combined with a plot summarising the maximum adjusted Rand index of all examined variants of that cost function. Similar plots for precision and recall as well as for the (N-best) average clustering quality can be found in the corresponding notebooks.

Clement cost function. The Clement cost function with weighted matches did not improve the clustering quality, but rather led to a much lower adjusted Rand index for most variants on almost all data sets. While many variants improved the precision notably, this usually came with massive losses in recall. On the contrary, the maximum clustering quality could be increased when excluding matches from the quality weighting. As can be seen in Figure S4, the adjusted Rand index could be improved on all Franzén data sets and – to differing extents – for all boosting functions. Inner boosting and especially the multiplicative boosting function provided the largest gains. However, there were no similar improvements on the Callahan data sets, on which the clustering quality corresponded to the one obtained with the quality-unweighted alignment-score mode for the most part. The increased adjusted Rand index was generally accompanied by an improved precision and a relatively stable recall. In addition to the maximum clustering quality, the quality-weighted cost function also increased the average quality.
The unboosted variant of the Clement cost function with unweighted matches was as good as or slightly better than the quality-unweighted alignment-score mode, in terms of both maximum and average adjusted Rand index. On Franzén data, the maximum adjusted Rand index was increased up to 1.3 % (on HC_V3-V4). A closer examination of the different boosted variants showed that a couple of variants with inner, multiplicative boosting (small to medium factor) worked best, usually increasing both the maximum and the average adjusted Rand index. The chosen representative (*v2 / mult / 15*) provided a good compromise between maximum and average clustering quality on the different data sets. While the differences in maximum quality were negligible on Callahan data, the improvement on the Franzén data sets varied between 2.3 and 8.4 %.

Converge-A cost function. As for the Clement cost function, variants with weighted matches did reduce the clustering quality in the vast majority of cases. Increases in precision were similarly coupled with more severe decreases in recall, allowing small isolated overall improvements at best. Figure S5 shows, however, that we again obtained an increased maximum clustering quality with unweighted matches. The adjusted

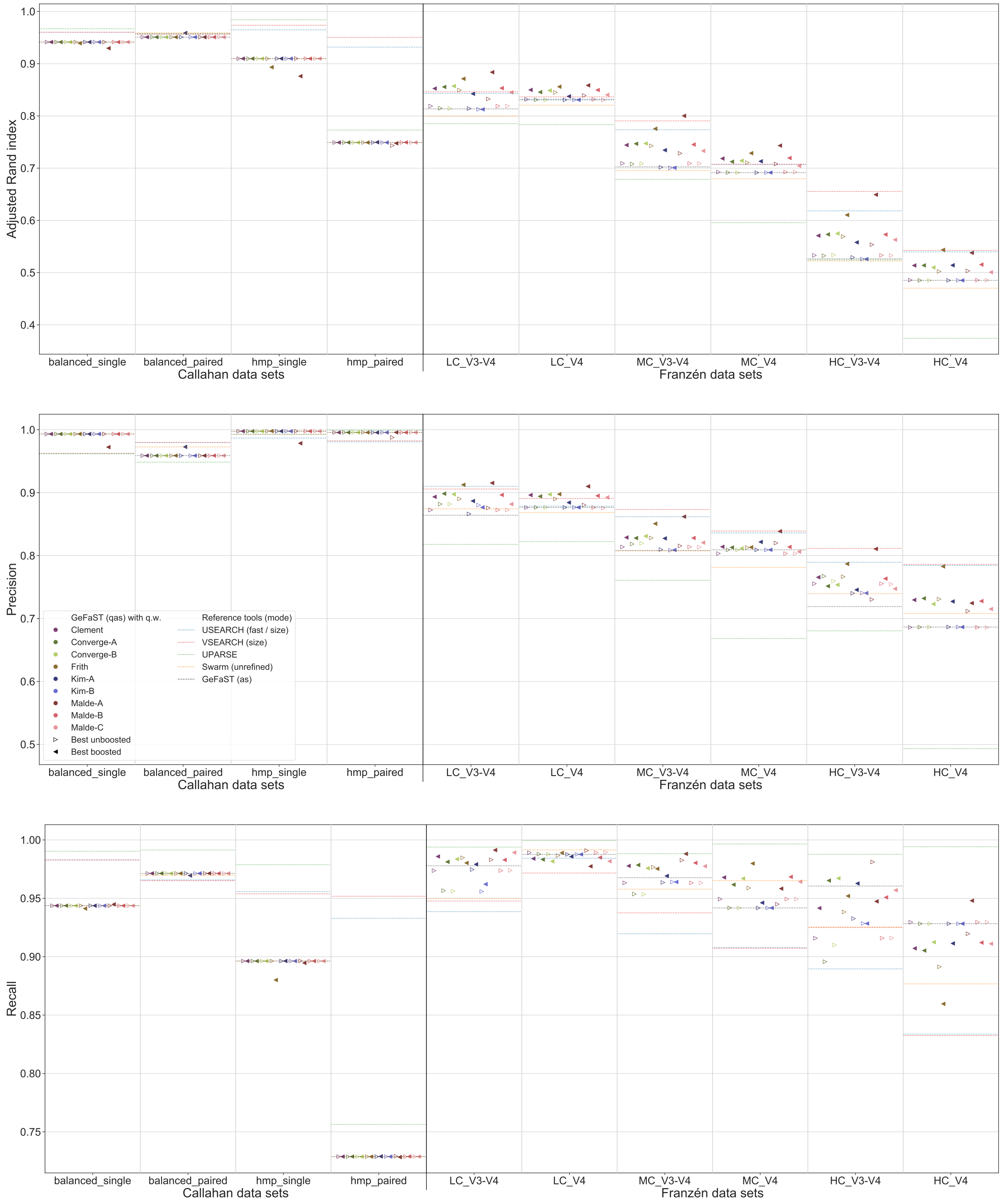

Figure S1: Clustering quality of GeFaST (modes: as, qas), USEARCH, VSEARCH, UPARSE and Swarm on the Callahan and Franzén data sets. Shows the maximum adjusted Rand index (and the corresponding precision and recall) of each tool or variant (see Table 4) per data set. For Franzén data, the maxima of the 10 actual data sets per combination of complexity and read type have been averaged. The plot of the maximum adjusted Rand index corresponds to Figure 4.

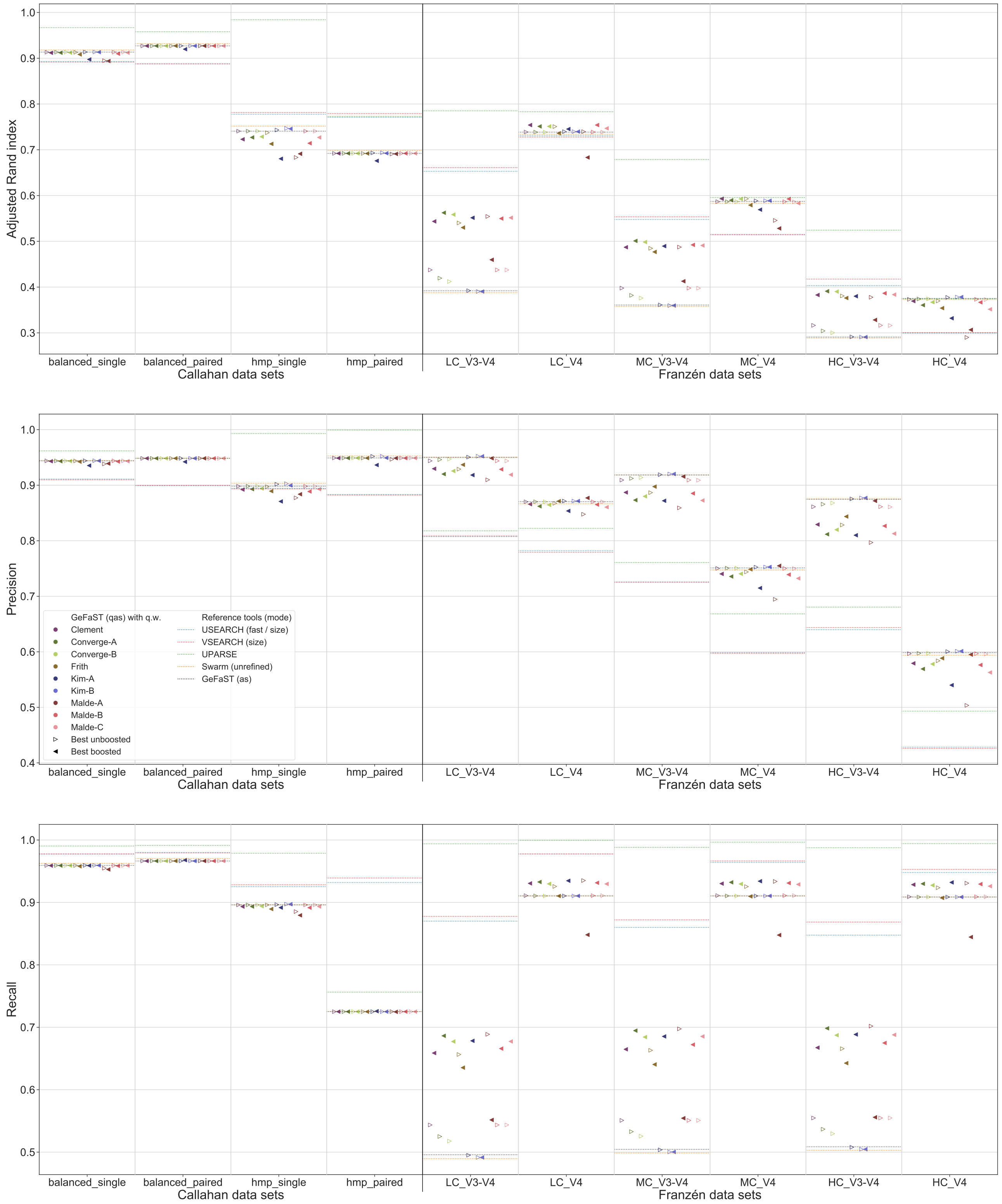

Figure S2: Clustering quality of GeFaST (modes: as, qas), USEARCH, VSEARCH, UPARSE and Swarm on the Callahan and Franzén data sets. Shows the average adjusted Rand index (and the corresponding precision and recall) of each tool or variant (see Table 4) per data set. For Franzén data, the average values of the 10 actual data sets per combination of complexity and read type have been averaged.

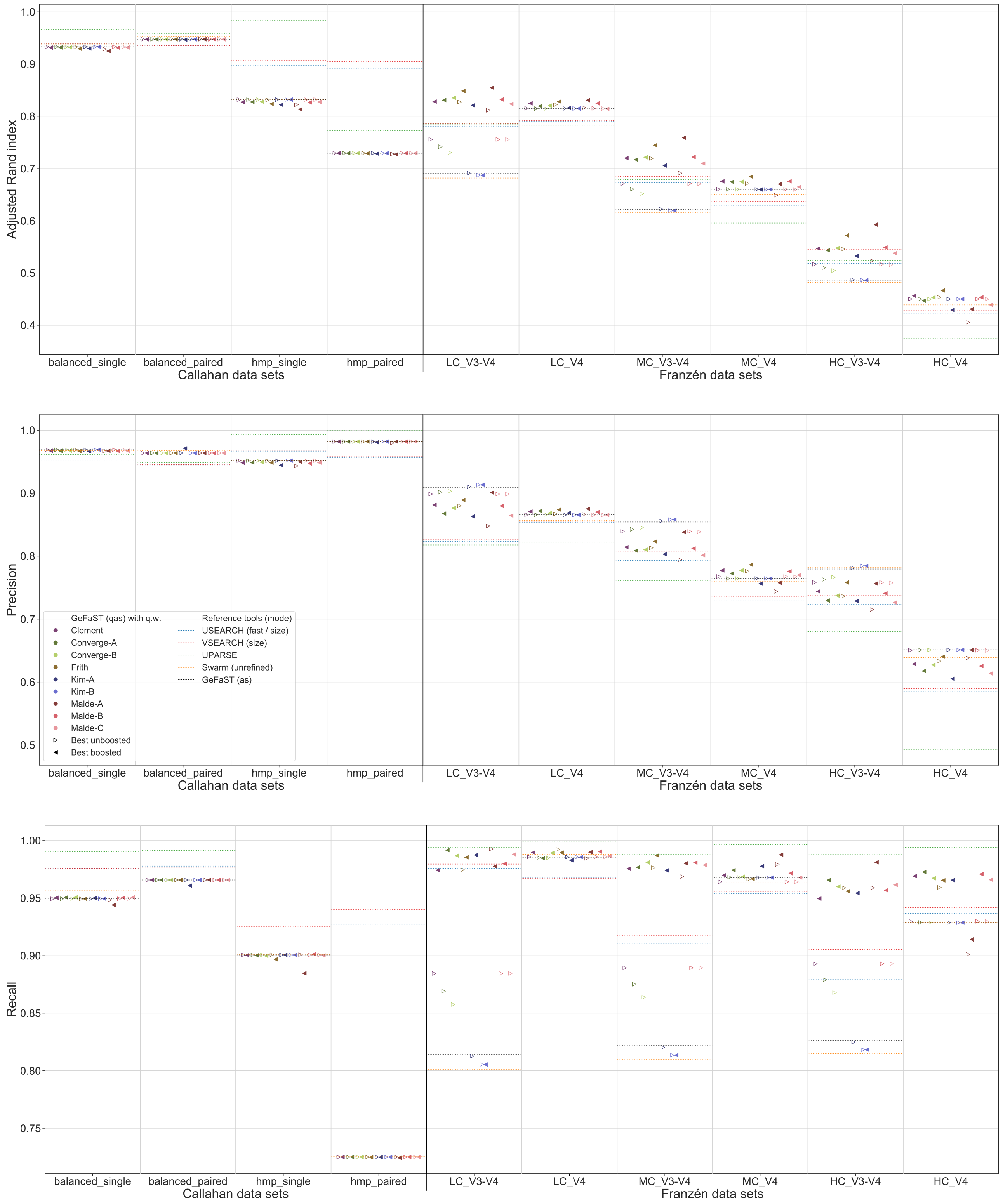

Figure S3: Clustering quality of GeFaST (modes: as, qas), USEARCH, VSEARCH, UPARSE and Swarm on the Callahan and Franzén data sets. Shows the N-best average adjusted Rand index (and the corresponding precision and recall) of each tool or variant (see Table 4) per data set. For Franzén data, the N-best average values of the 10 actual data sets per combination of complexity and read type have been averaged.

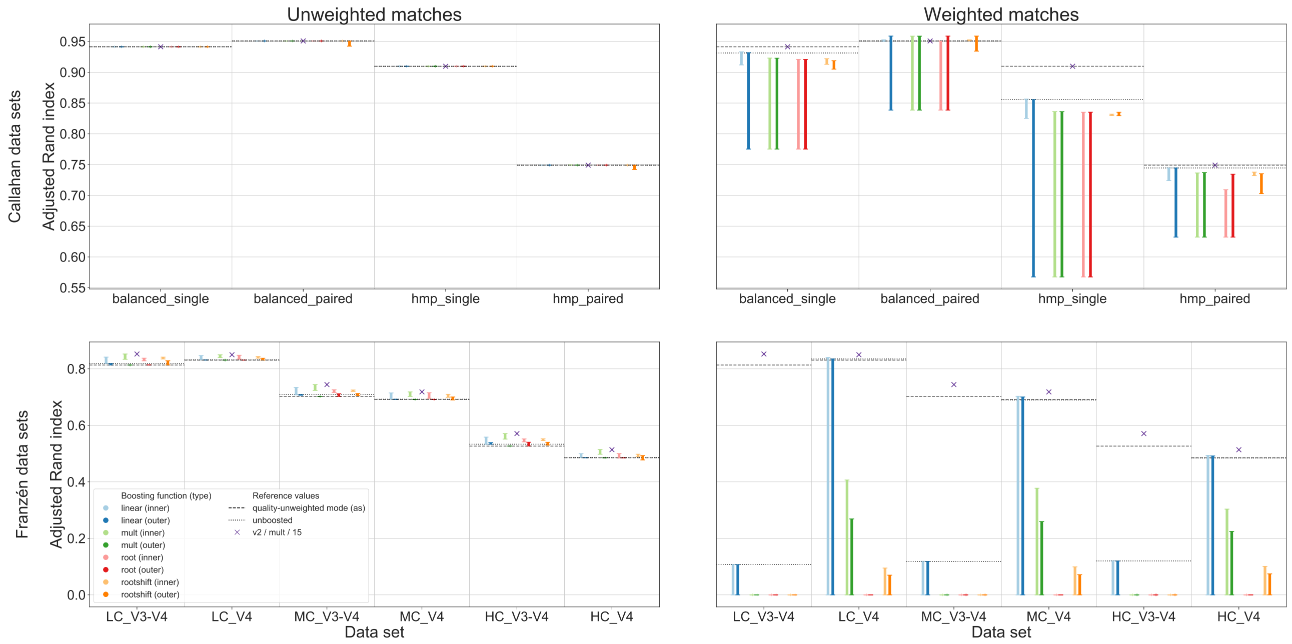

Figure S4: Maximum clustering quality of the examined variants of the quality-weighted Clement cost function, with (right column) and without (left) weighted matches, in GeFaST's quality alignment-score mode. Each vertical line corresponds to a group of variants using the same boosting function (e.g. *root*) and boosting type (inner or outer) and depicts the range of adjusted Rand index values covered by varying the boosting parameter (e.g. the degree of the root). The cross marks the clustering quality of the chosen representative of the Clement cost function on the different data sets.

Rand index could be improved on all Franzén data sets and for all boosting functions. Here, outer boosting was the preferred boosting type. On the Callahan data sets, the clustering quality of the different variants once more corresponded in general to the quality-unweighted alignment score mode. As before, the increased adjusted Rand index was generally accompanied by an improved precision and a relatively stable recall, and the adjusted Rand index was also improved on average.

The unboosted variant of the cost function with unweighted matches was usually at least as good as the quality-unweighted mode in terms of maximum and average adjusted Rand index. The maximum adjusted Rand index was increased up to 1.1 % on Franzén data (`HC_V3-V4`). Concerning the boosted variants, multiplicative boosting with small factors and extracting roots of medium degree led to the largest improvements of the maximum clustering quality. The average adjusted Rand index was, for the most part, only increased on Franzén data. The variant *v4 / mult / 15* was chosen as the representative as it provided the best combination of maximum and average clustering quality. The differences in maximum quality were again minute on Callahan data. On the Franzén data sets, it attained improvements between 1.8 % and 8.9 %.

Converge-B cost function. Overall, the quality-weighted Converge-B cost function behaved very similar to the Converge-A version. Weighted matches worsened the clustering quality for the same reasons, while variants with unweighted matches were able to improve the maximum clustering quality (Figure S6). The adjusted Rand index again increased on all Franzén data sets and for all boosting functions, especially with outer boosting, while it remained largely unchanged on Callahan data. As for Converge-A, we observed an improved precision and rather stable recall related to the improvements in adjusted Rand index.

The unboosted variant of the cost function with unweighted matches was the preferred choice and was again usually at least as good as the quality-unweighted mode in terms of maximum and average adjusted Rand index. On Franzén data, the maximum adjusted Rand index was increased up to 1.4 % (on `HC_V3-V4`). Boosted variants using the multiplicative function with small factors and outer boosting were, as before, the variants providing the best results in terms of the maximum and average clustering quality on Franzén data sets, but they were also not able to deviate (positively) from the quality-unweighted mode on Callahan data. The chosen variant (*v4 / mult / 15*, the same as for Converge-A) allowed to increase the maximum adjusted Rand index on the Franzén data sets by 2.2 % to 9.2 %.

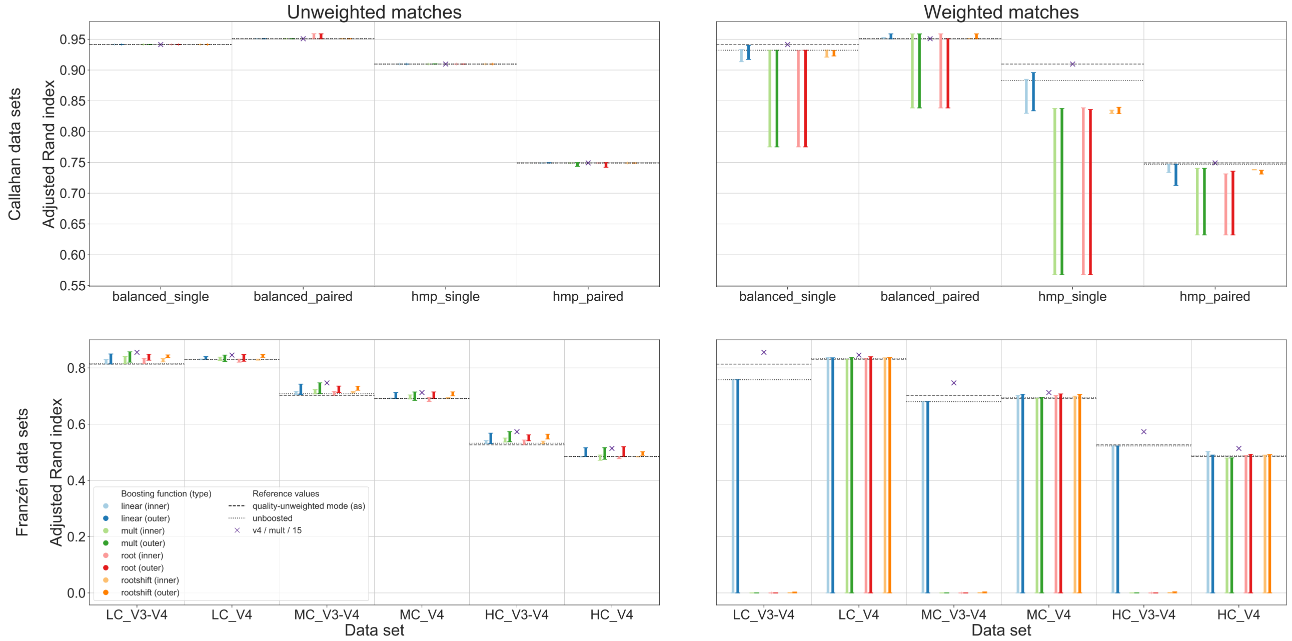

Figure S5: Maximum clustering quality of the examined variants of the quality-weighted Converge-A cost function, with (right column) and without (left) weighted matches, in GeFaST's quality alignment-score mode. Each vertical line corresponds to a group of variants using the same boosting function (e.g. *root*) and boosting type (inner or outer) and depicts the range of adjusted Rand index values covered by varying the boosting parameter (e.g. the degree of the root). The cross marks the clustering quality of the chosen representative of the Converge-A cost function on the different data sets.

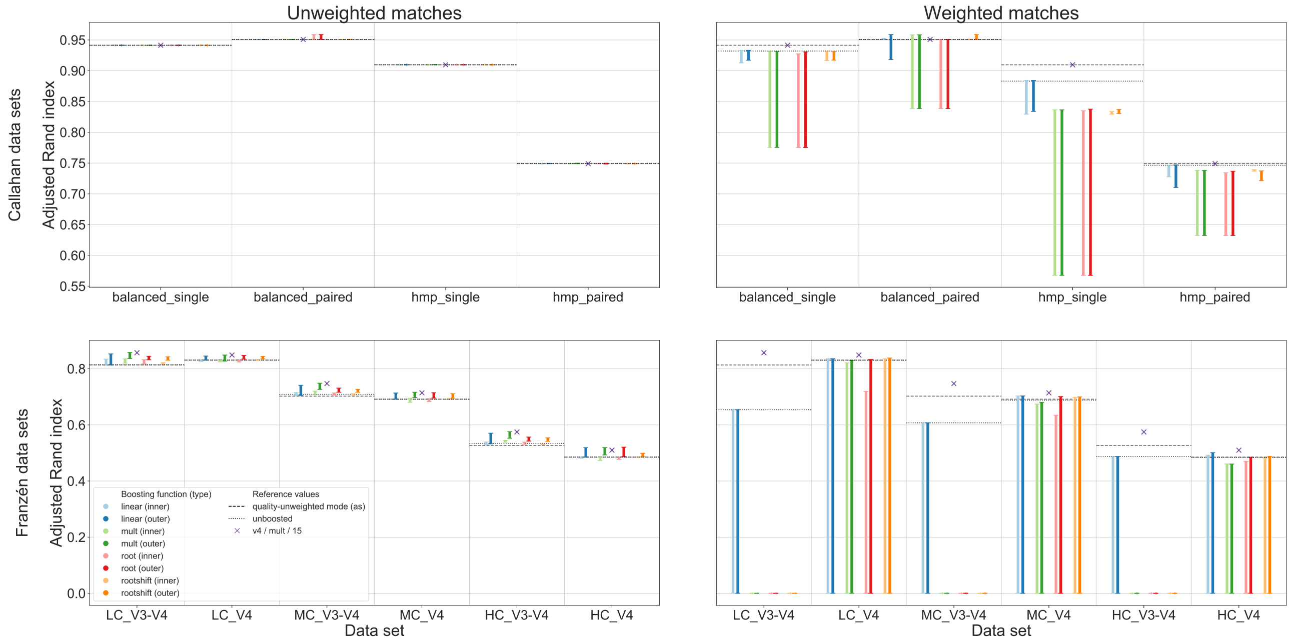

Figure S6: Maximum clustering quality of the examined variants of the quality-weighted Converge-B cost function, with (right column) and without (left) weighted matches, in GeFaST's quality alignment-score mode. Each vertical line corresponds to a group of variants using the same boosting function (e.g. *root*) and boosting type (inner or outer) and depicts the range of adjusted Rand index values covered by varying the boosting parameter (e.g. the degree of the root). The cross marks the clustering quality of the chosen representative of the Converge-B cost function on the different data sets.

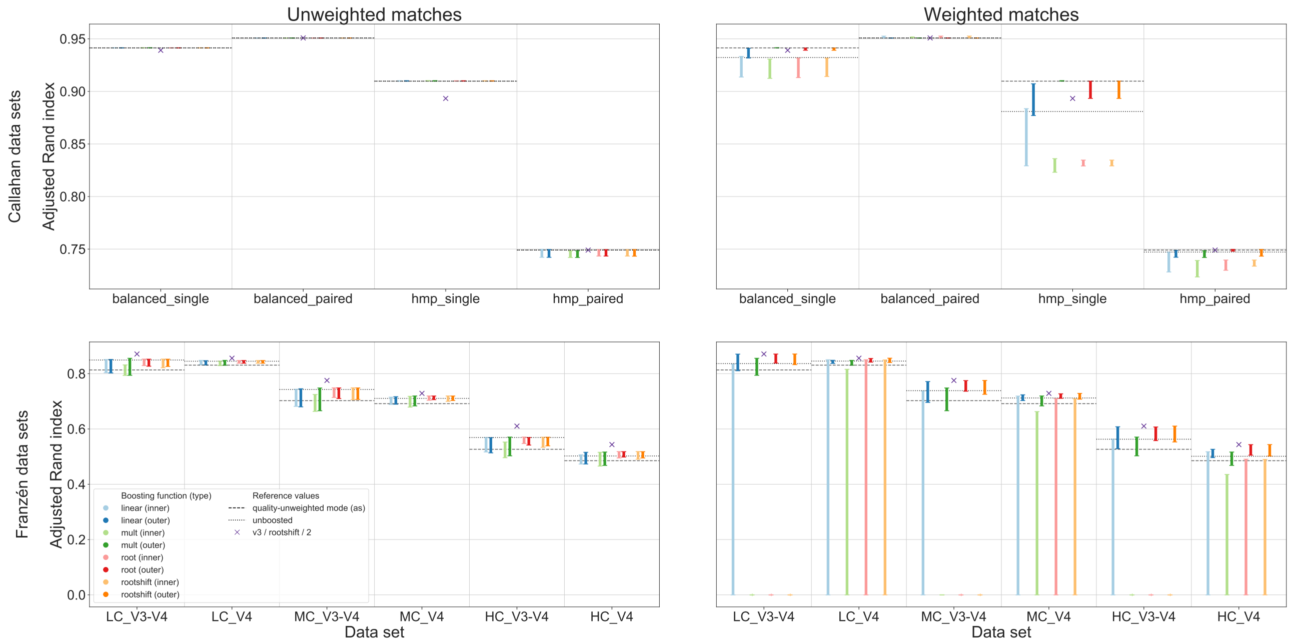

Figure S7: Maximum clustering quality of the examined variants of the quality-weighted Frith cost function, with (right column) and without (left) weighted matches, in GeFaST's quality alignment-score mode. Each vertical line corresponds to a group of variants using the same boosting function (e.g. *root*) and boosting type (inner or outer) and depicts the range of adjusted Rand index values covered by varying the boosting parameter (e.g. the degree of the root). The cross marks the clustering quality of the chosen representative of the Frith cost function on the different data sets.

Frith cost function. In contrast to most other cost functions, the quality-weighted Frith cost function worked with both weighted and unweighted matches (Figure S7) – at least on Franzén data. On Callahan data, however, the maximum clustering quality could also not be improved. The unboosted variants, regardless of using weighted or unweighted matches, already provided quite large improvements in the adjusted Rand index. Even the best boosted variants with unweighted matches hardly exceeded the maximum adjusted Rand index of the unboosted variant on the different Franzén data sets. For weighted matches, in turn, variants using any boosting function with outer boosting were able to further enhance the clustering quality, but there were also some parameter choices that led to a lower adjusted Rand index value (especially with multiplicative boosting) The increased adjusted Rand index was once more often accompanied by an improved precision and a largely stable recall. The average clustering quality of the (best) boosted variants tended to be decreased slightly compared to the quality-unweighted mode (except for the `V3-V4` data sets), especially when averaging over the whole threshold range. When considering the more relevant portion of the threshold range (N-best average), the reduction was reduced or disappeared.

Both unboosted variants of the Frith cost function achieved a similar maximum clustering quality but the one with unweighted matches was more robust over the threshold range. On Franzén data, the maximum adjusted Rand index increased between 1.7 and 8.1 %. Examining the boosted variants more closely, *v3 / rootshift / 2* emerged as one of the best choices on Franzén data, allowing larger increases in maximum clustering quality, while keeping the losses in average clustering quality quite small. Using this boosted variant, the improvements in maximum adjusted Rand index varied between 3.0 and 15.9 %. On Callahan data (especially the `single` data sets), the unboosted variant or the quality-unweighted mode were preferable.

Kim-A cost function. Variants of the Kim-A cost function with weighted matches lowered the clustering quality in almost all cases. Similar to Clement and the Converge cost functions, these reductions stemmed from extremely large drops in recall. Figure S8 shows, however, that there were again variants with unweighted matches and inner boosting that improved the maximum adjusted Rand index on Franzén data and, at least, were as good as the quality-unweighted mode on Callahan data. The root boosting function (followed by the multiplicative and linear ones) allowed the largest gains by increasing the precision and keeping the recall stable. In addition to the maximum clustering quality, the better variants also increased the average quality on the `V3-V4` data sets, but suffered from reductions on some `V4` data sets.

The unboosted variant of the Kim-A cost function with unweighted matches was as good as or very slightly

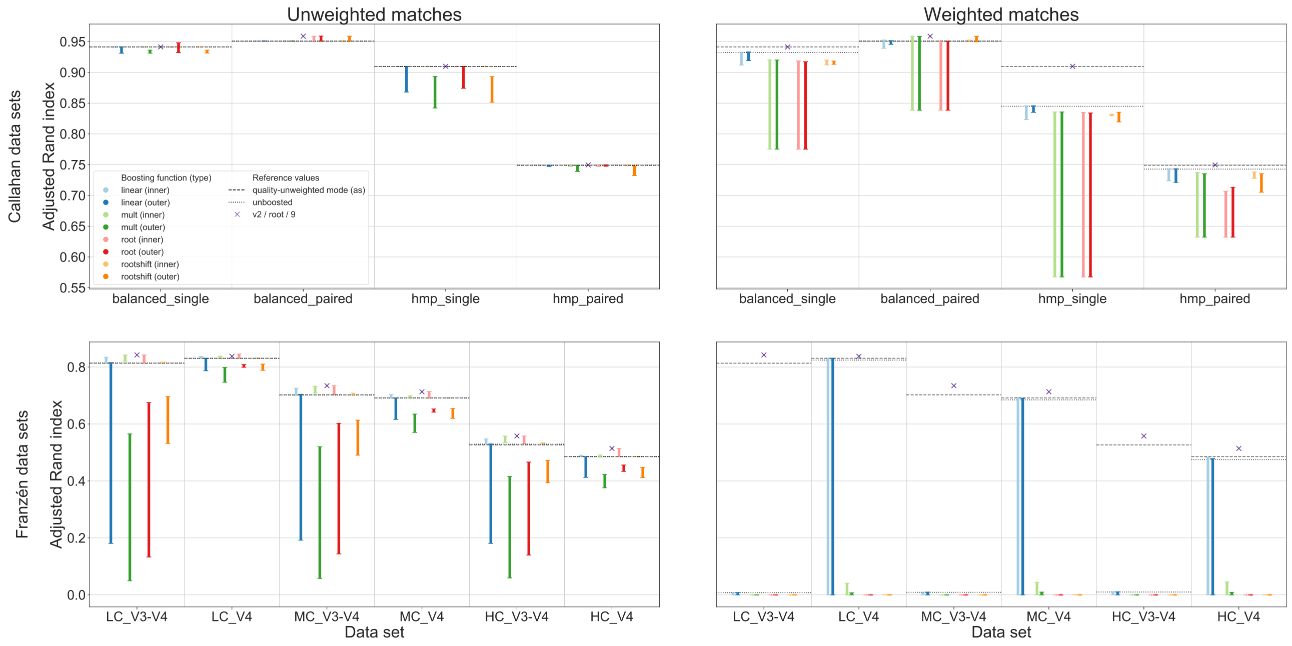

Figure S8: Maximum clustering quality of the examined variants of the quality-weighted Kim-A cost function, with (right column) and without (left) weighted matches, in GeFaST's quality alignment-score mode. Each vertical line corresponds to a group of variants using the same boosting function (e.g. *root*) and boosting type (inner or outer) and depicts the range of adjusted Rand index values covered by varying the boosting parameter (e.g. the degree of the root). The cross marks the clustering quality of the chosen representative of the Kim-A cost function on the different data sets.

better than the quality-unweighted alignment-score mode, in terms of both maximum and average adjusted Rand index. On Franzén data, the maximum adjusted Rand index was increased up to 0.5 % (on `HC_V3-V4`). With respect to the boosted variants, the root boosting function (inner boosting) with high degrees worked best and the chosen representative (*v2 / root / 9*) provided the largest improvements in maximum adjusted Rand index (0.8 to 6.0 %), while also keeping the average clustering quality relatively close to the quality-unweighted mode.

Kim-B cost function. The quality-weighted Kim-B cost function behaved almost identical to the Kim-A version. The single major difference comprises the clustering quality of the boosted variants with inner boosting. While they provided improvements for Kim-A, the maximum adjusted Rand index was, at best, similar to the one of the quality-unweighted alignment-score mode for Kim-B (Figure S9). Similarly, there was no increased average clustering quality on the Franzén data sets.
The unboosted variant with unweighted matches was still the better unboosted choice but did no longer provide the slight improvements on `V3-V4` data seen for Kim-A. Also, there was no boosted variant that worked well on all data sets. Some variants, such as *v4 / root / 7*, provided small improvements (below one per cent) in the maximum adjusted Rand index on Callahan data, but lost up to three quarters of it on Franzén data. Other variants (e.g. *v4 / linear / 20*) could avoid such vast reductions but, in turn, did not improve the clustering quality on the Callahan data.

Malde-A cost function. Similar to Frith, the Malde-A cost function worked with both weighted and unweighted matches (Figure S10). On Callahan data, a few improvements in the maximum clustering quality were observed but these were limited to `balanced_paired`. The unboosted variants, regardless of using weighted or unweighted matches, improved the adjusted Rand index on all Franzén data sets. Boosting could increase the clustering quality for both weighted and unweighted matches, but had a stronger effect on variants with weighted matches. The largest gains in maximum adjusted Rand index were observed for the linear and (shifted) root boosting functions as outer boosting, due to an increased precision and a relatively stable recall. Also, the average clustering quality was not improved in similar ways, especially on `V4` data sets.
In general, the unboosted variant with weighted matches achieved a higher or at least similar maximum and average clustering quality compared to the one with unweighted matches. On Franzén data, the maximum

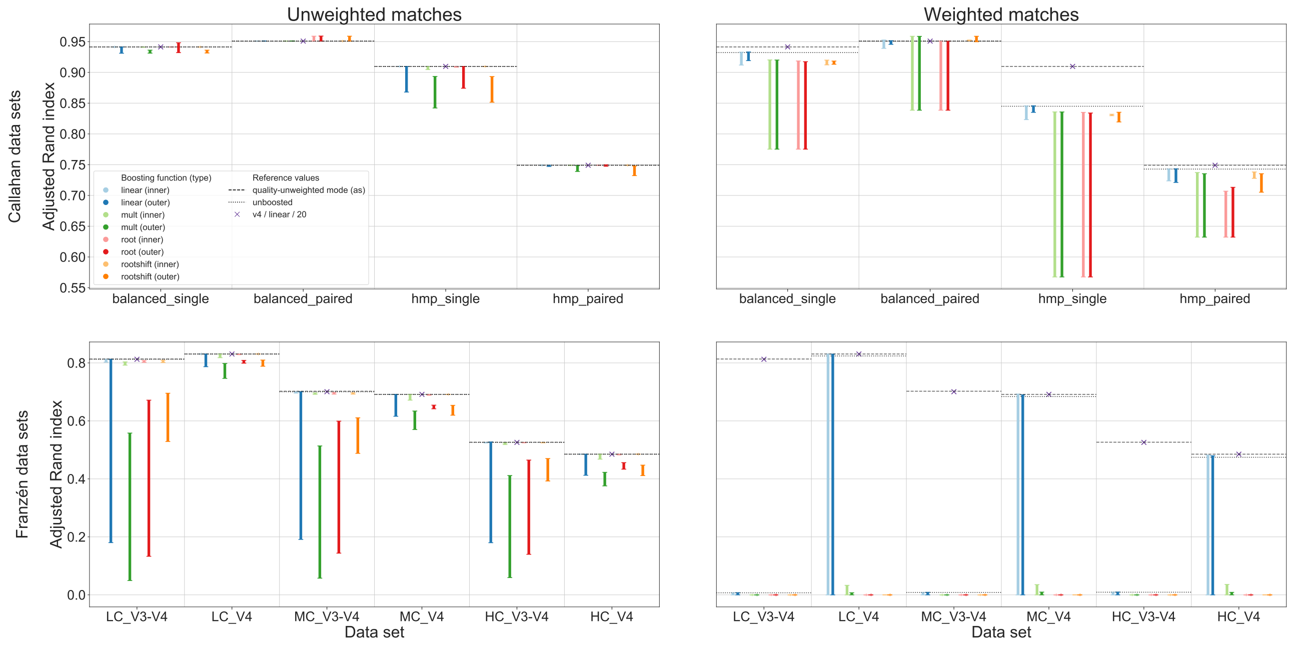

Figure S9: Maximum clustering quality of the examined variants of the quality-weighted Kim-B cost function, with (right column) and without (left) weighted matches, in GeFaST's quality alignment-score mode. Each vertical line corresponds to a group of variants using the same boosting function (e.g. *root*) and boosting type (inner or outer) and depicts the range of adjusted Rand index values covered by varying the boosting parameter (e.g. the degree of the root). The cross marks the clustering quality of the chosen representative of the Kim-B cost function on the different data sets.

adjusted Rand index was improved by up to 5.1 %. An inspection of the different boosted variants showed that *root* with small to medium degrees provided the best results, usually increasing the maximum adjusted Rand index as well as its average over the more relevant portion of the threshold range (N-best average). For example, the maximum adjusted Rand index increased between 3.3 and 23.3 % for the chosen variant (*v3 / root / 3*).

Malde-B cost function. The Malde-B cost function with weighted matches did not improve the clustering quality (except on `balanced_paired`), but rather led to a much lower adjusted Rand index for most variants on almost all data sets (especially on Franzén data). While many variants improved the precision notably, this was usually accompanied by a massive loss in recall. On the contrary, the maximum clustering quality increased when excluding matches from the quality weighting. As shown in Figure S11, the adjusted Rand index could be improved on all Franzén data sets and for all boosting functions. Both inner and outer boosting, especially with the multiplicative boosting function, provided notable gains. On the Callahan data sets, however, the clustering quality essentially correspondeded to the one obtained with the quality-unweighted alignment-score mode. The increased adjusted Rand index was most often accompanied by an improved precision and a relatively stable recall. In addition to the maximum clustering quality, the better variants also increased the average quality.

The unboosted variant with unweighted matches was as good as or slightly better than the quality-unweighted mode (for the most part in both maximum and average adjusted Rand index). On Franzén data, the maximum adjusted Rand index was increased up to 1.3 % (on `HC_V3-V4`). Examining the boosted variants, we found that variants with outer, multiplicative boosting (with a small to medium factor) worked best and usually increased both the maximum and the average adjusted Rand index. These variants differed only very little and the chosen representative (*v4 / mult / 20*) provided a good compromise between maximum and average clustering quality on the different data sets. While the improvements on the Franzén data sets varied between 2.3 and 8.8 %, the differences in maximum quality were negligible on Callahan data.

Malde-C cost function. As for the majority of cost functions, the variants with weighted matches reduced the clustering quality in almost all cases. Underlying increases in precision were usually coupled with larger decreases in recall, allowing small isolated overall improvements at best. Figure S12 depicts that we could, in

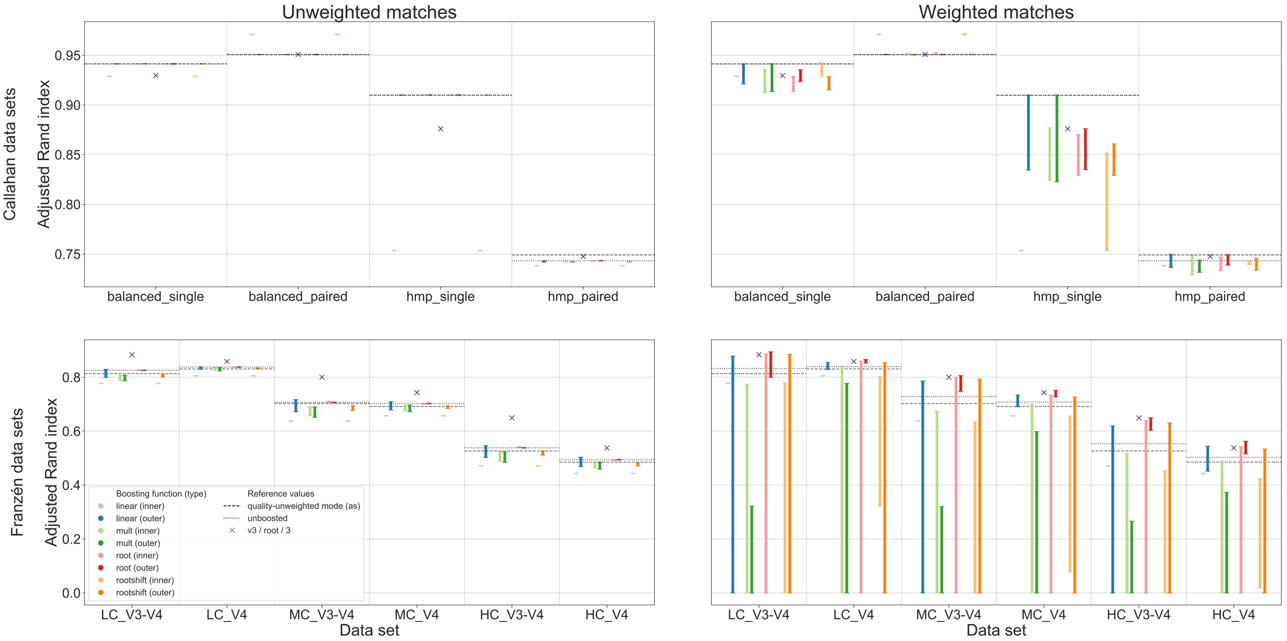

Figure S10: Maximum clustering quality of the examined variants of the quality-weighted Malde-A cost function, with (right column) and without (left) weighted matches, in GeFaST's quality alignment-score mode. Each vertical line corresponds to a group of variants using the same boosting function (e.g. *root*) and boosting type (inner or outer) and depicts the range of adjusted Rand index values covered by varying the boosting parameter (e.g. the degree of the root). The cross marks the clustering quality of the chosen representative of the Malde-A cost function on the different data sets.

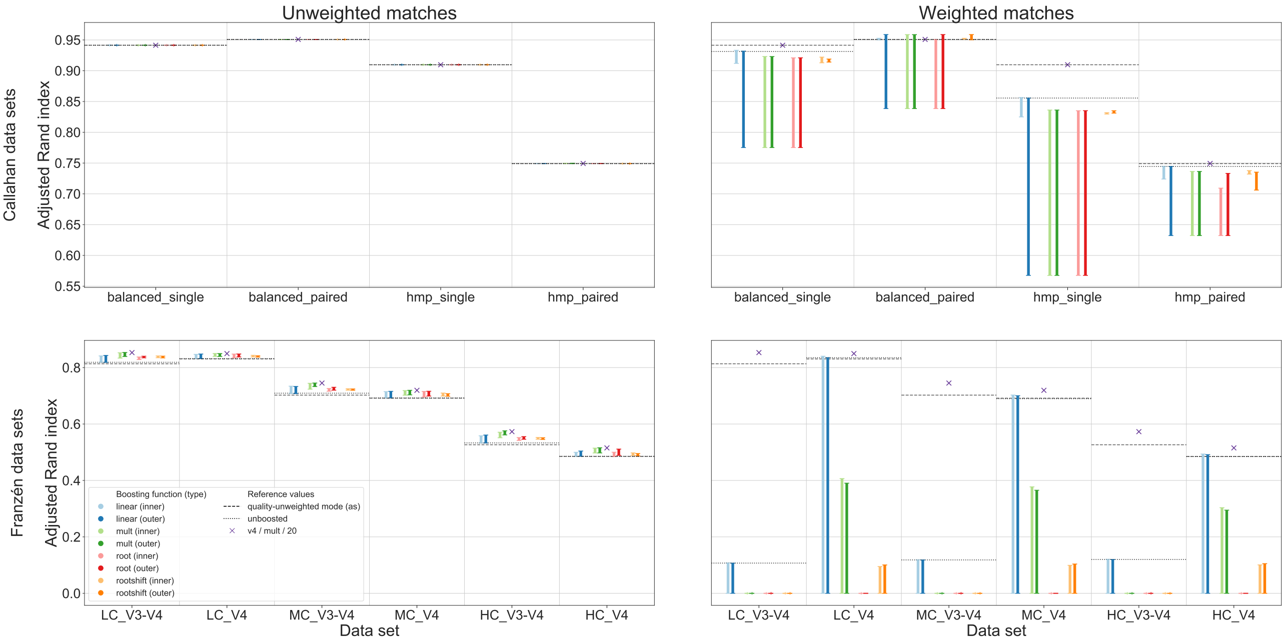

Figure S11: Maximum clustering quality of the examined variants of the quality-weighted Malde-B cost function, with (right column) and without (left) weighted matches, in GeFaST's quality alignment-score mode. Each vertical line corresponds to a group of variants using the same boosting function (e.g. *root*) and boosting type (inner or outer) and depicts the range of adjusted Rand index values covered by varying the boosting parameter (e.g. the degree of the root). The cross marks the clustering quality of the chosen representative of the Malde-B cost function on the different data sets.

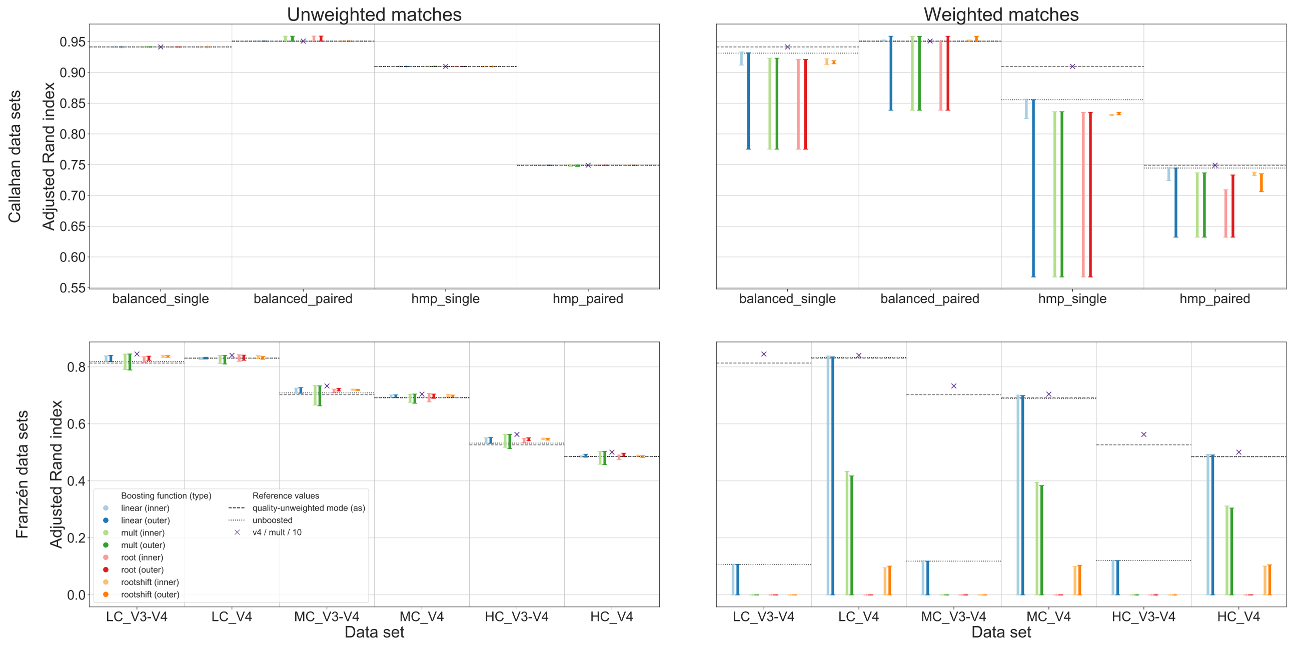

Figure S12: Maximum clustering quality of the examined variants of the quality-weighted Malde-C cost function, with (right column) and without (left) weighted matches, in GeFaST's quality alignment-score mode. Each vertical line corresponds to a group of variants using the same boosting function (e.g. *root*) and boosting type (inner or outer) and depicts the range of adjusted Rand index values covered by varying the boosting parameter (e.g. the degree of the root). The cross marks the clustering quality of the chosen representative of the Malde-C cost function on the different data sets.

contrast, obtain a higher maximum clustering quality when using unweighted matches. The adjusted Rand index could be improved on all Franzén data sets and – to differing extents – for all boosting functions. Similar to Malde-B, the differences between outer and inner boosting were small in most cases and the multiplicative boosting function led to the largest gains. On the Callahan data sets, the clustering quality of the different variants once more corresponded in general to the quality-unweighted alignment score mode. The increased adjusted Rand index was again most often accompanied by an improved precision and a relatively stable recall, and the better variants also improved the average clustering quality.

Once more the unboosted variant with unweighted matches was as good as or slightly better than the quality-unweighted mode – usually in terms of both maximum and average adjusted Rand index. On Franzén data, the maximum adjusted Rand index was increased up to 1.2 % (on `HC_V3-V4`). For the boosted variants, we found that variants with outer and multiplicative boosting (using small factors) provided the best results and usually increased the maximum as well as the average adjusted Rand index. The chosen representative (*v4 / mult / 10*) represents a good compromise between maximum and average clustering quality on the different data sets. While the improvements on the Franzén data sets varied between 2.3 and 8.8 %, On the Callahan data, the differences in maximum quality were again negligible, but on the improvements on the Franzén data sets were more notable, ranging from 1.2 to 6.9 %.

C.2.2 Quality Levenshtein mode

The maximum, average and N-best average clustering quality of the best variants of GeFaST in quality Levenshtein mode are shown in Figures S13, S14 and S15, respectively. The remarks on each quality-weighted cost function are again accompanied by a plot summarising the maximum adjusted Rand index of all examined variants of the respective cost function and similar plots for precision and recall as well as for the (N-best) average clustering quality are available in the corresponding notebooks.

Clement cost function. The Clement cost function worked with both weighted and unweighted matches at least as good as the quality-unweighted Levenshtein mode. Excluding matches from the quality weighting, however, did not lead to notable improvements in the clustering quality on Callahan or Franzén data. On the contrary, the maximum clustering quality could be improved when weighting matches as well. Figure S16 shows how the adjusted Rand index increased on all Franzén data sets (especially for inner, multiplicative

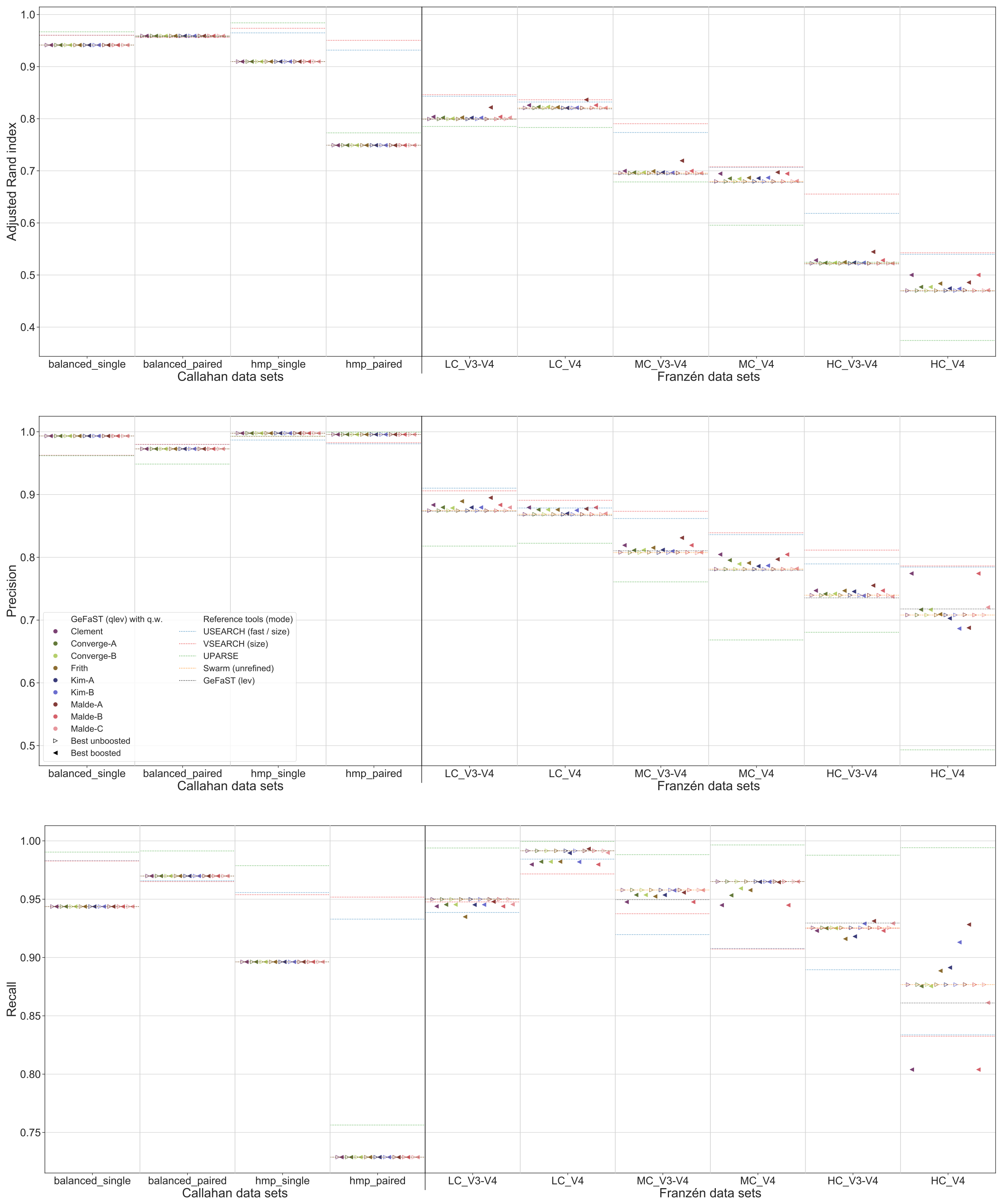

Figure S13: Clustering quality of GeFaST (modes: `lev`, `qlev`), USEARCH, VSEARCH, UPARSE and Swarm on the Callahan and Franzén data sets. Shows the maximum adjusted Rand index (and the corresponding precision and recall) of each tool or variant (see Table 4) per data set. For Franzén data, the maxima of the 10 actual data sets per combination of complexity and read type have been averaged. The plot of the maximum adjusted Rand index corresponds to Figure 5.

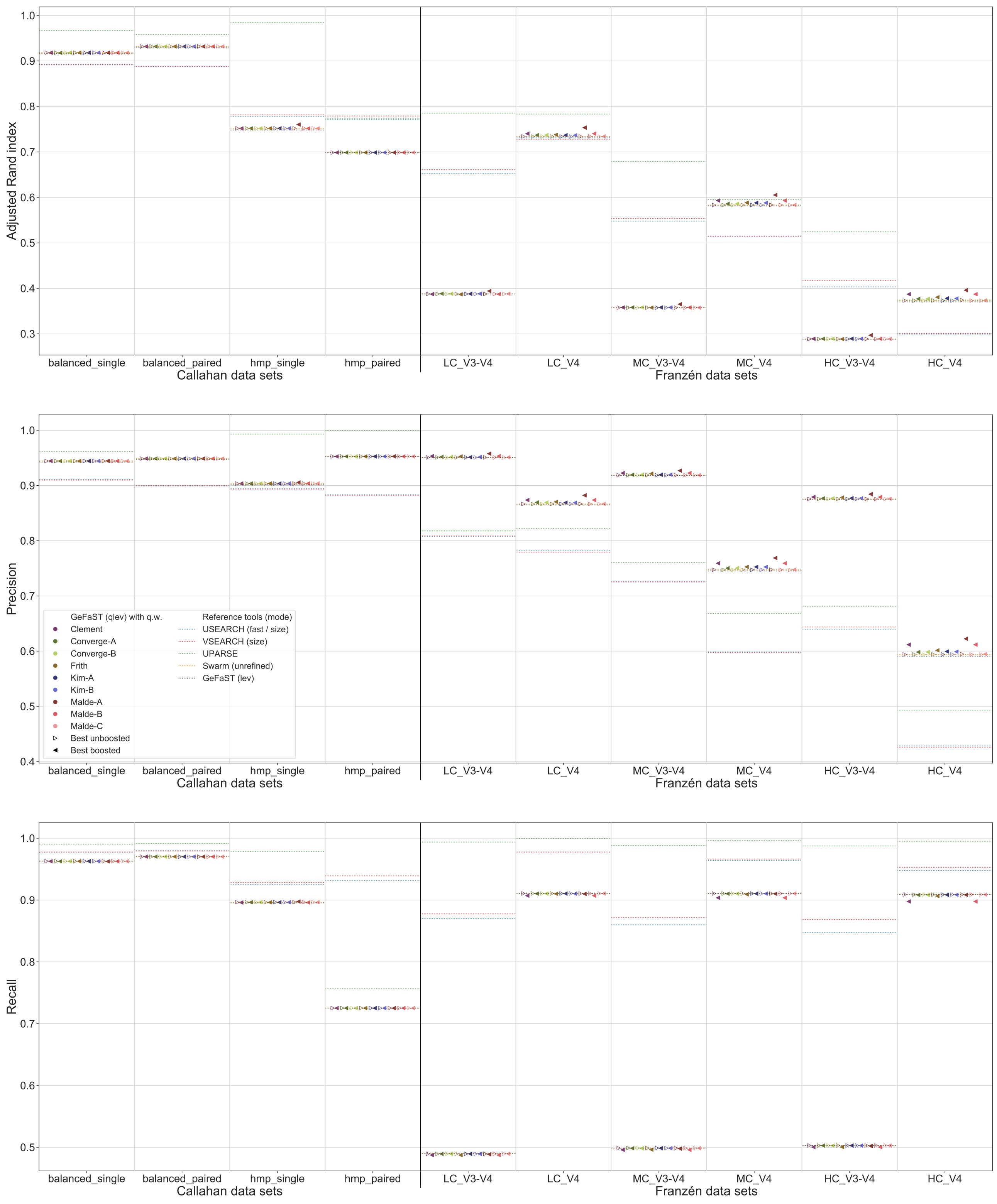

Figure S14: Clustering quality of GeFaST (modes: `lev`, `qlev`), USEARCH, VSEARCH, UPARSE and Swarm on the Callahan and Franzén data sets. Shows the average adjusted Rand index (and the corresponding precision and recall) of each tool or variant (see Table 4) per data set. For Franzén data, the average values of the 10 actual data sets per combination of complexity and read type have been averaged.

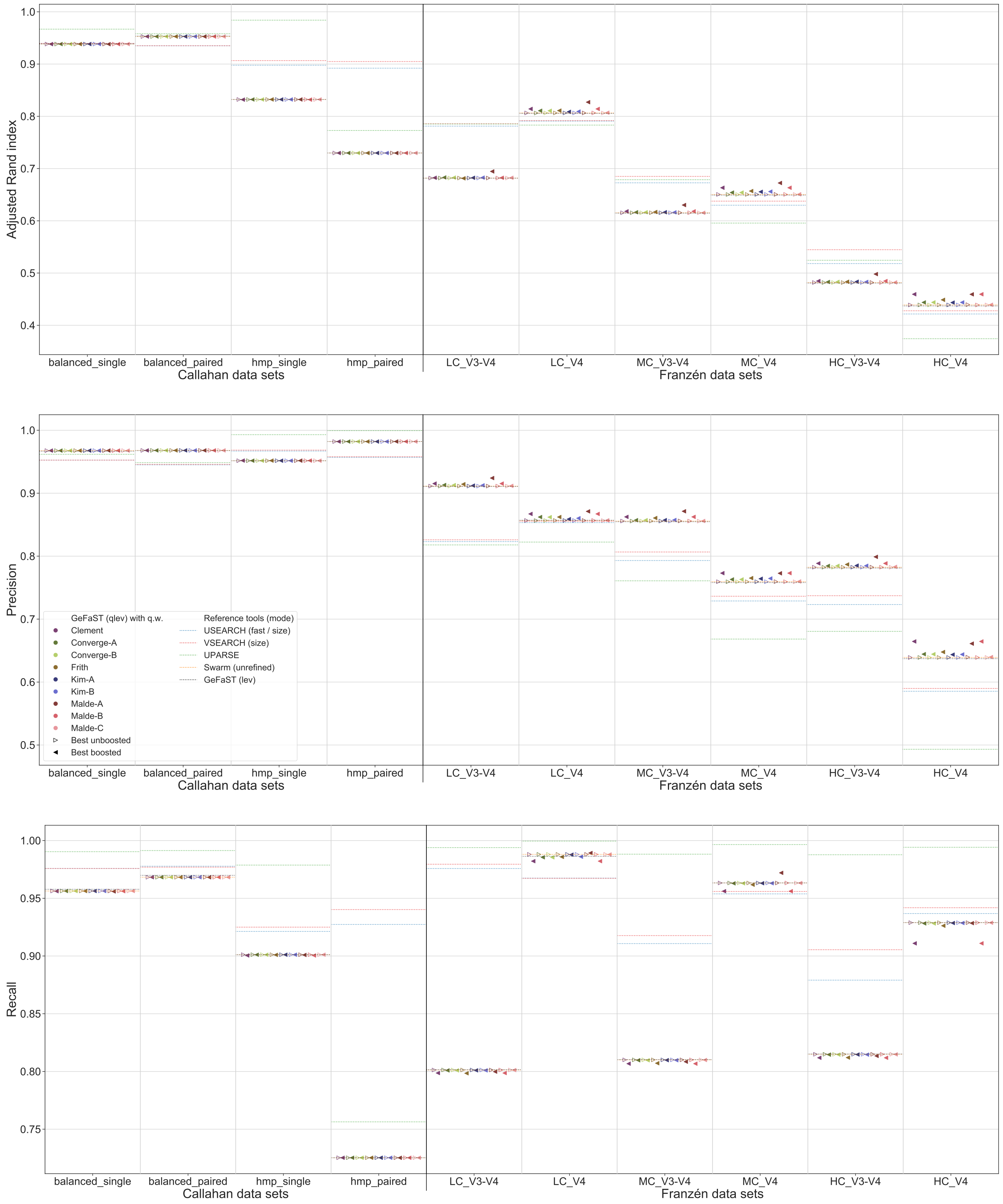

Figure S15: Clustering quality of GeFaST (modes: `lev`, `qlev`), USEARCH, VSEARCH, UPARSE and Swarm on the Callahan and Franzén data sets. Shows the N-best average adjusted Rand index (and the corresponding precision and recall) of each tool or variant (see Table 4) per data set. For Franzén data, the N-best average values of the 10 actual data sets per combination of complexity and read type have been averaged.

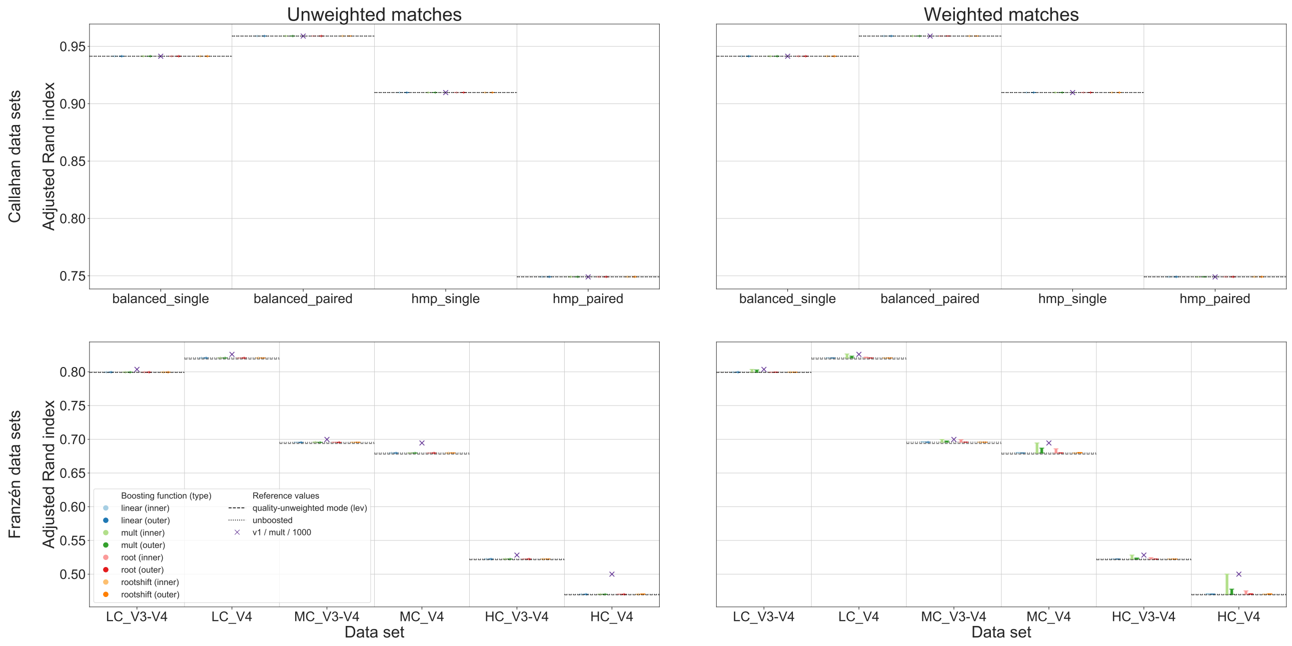

Figure S16: Maximum clustering quality of the examined variants of the quality-weighted Clement cost function, with (right column) and without (left) weighted matches, in GeFaST's quality Levenshtein mode. Each vertical line corresponds to a group of variants using the same boosting function (e.g. *root*) and boosting type (inner or outer) and depicts the range of adjusted Rand index values covered by varying the boosting parameter (e.g. the degree of the root). The cross marks the clustering quality of the chosen representative of the Clement cost function on the different data sets.

boosting) and that no similar improvements were achieved on Callahan data. The higher adjusted Rand index usually involved an improved precision and a reduced recall. In addition to the maximum clustering quality, the better variants also increased the average quality, in particular on the V4 data sets.
Both unboosted variants of the Clement cost function attained essentially the same clustering quality and were very slightly better than the Levenshtein mode. On Franzén data, the unboosted variant with weighted matches increased the maximum adjusted Rand index up to 0.3 %. Of the boosted variants, multiplicative reinforcement with very high factors worked best, usually improving both the maximum and average adjusted Rand index. The best variant, *v1 / mult / 1000*, allowed to increase the maximum adjusted Rand index by 0.6 to 6.6 % on Franzén data, while the differences were negligible on the Callahan data sets.

Converge-A cost function. Similar to Clement, the Converge-A cost function worked with both weighted and unweighted matches and achieved at least the same clustering quality as the Levenshtein mode but did not result in an actually improved clustering quality when using unweighted matches. As can be seen in Figure S17, the maximum clustering quality could be increased at least slightly when weighting matches. While no improvements were obtained on the Callahan data sets, the adjusted Rand index rose on all Franzén data sets, in particular when using multiplicative reinforcement as the outer boosting function. The increased adjusted Rand index again involved an improved precision and an often reduced recall. Similar advancements were observed in terms of the average quality (especially on the V4 data sets).
Both unboosted variants did not differ from each other in the clustering quality and provided a very small improvement to the Levenshtein mode. On Franzén data, the variant with weighted matches improved the maximum adjusted Rand index by up to 0.3 %. A comparison of the boosted variants showed that very high factors were the best choice for the multiplicative boosting function and led to a higher maximum and average adjusted Rand index on the Franzén data sets. The best variant in terms of both maximum and average clustering quality was *v3 / mult / 1000* and attained improvements between 0.4 and 1.6 %. On the Callahan data, the deviations from the quality of the Levenshtein mode were again minute.

Converge-B cost function. The clustering quality of the Converge-B cost function was very similar to the one of the Converge-A version. Using unweighted matches did not change the clustering quality compared to the Levenshtein mode regardless of the data set, while weighting them resulted in small improvements on

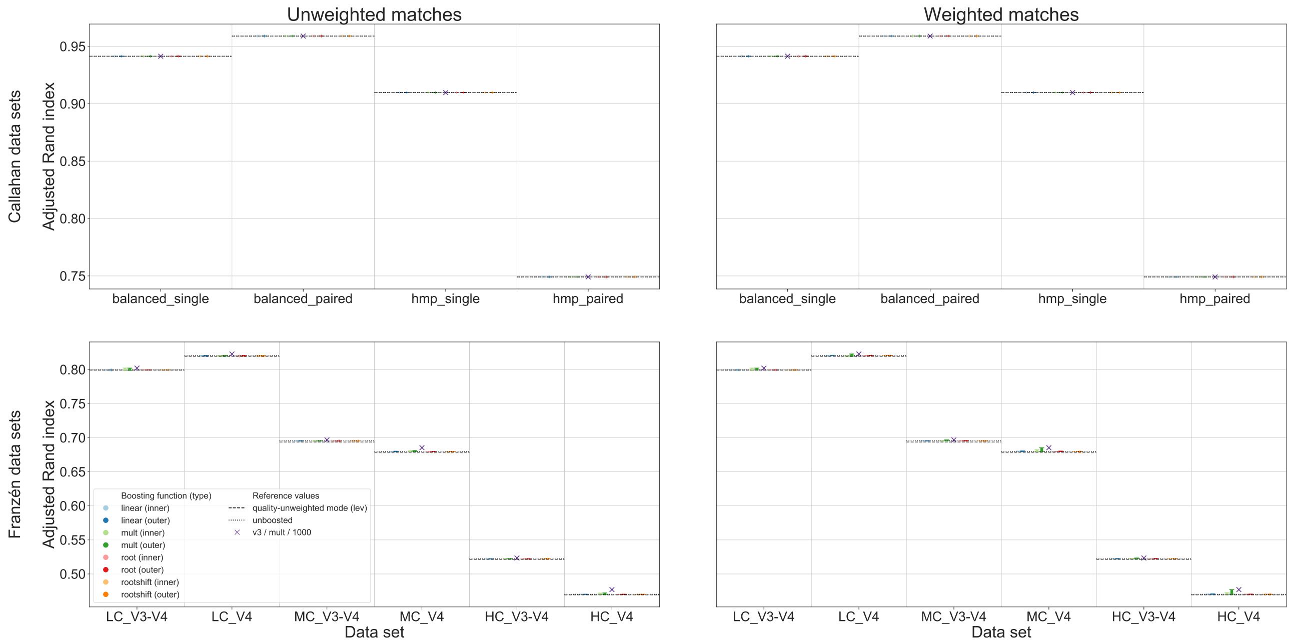

Figure S17: Maximum clustering quality of the examined variants of the quality-weighted Converge-A cost function, with (right column) and without (left) weighted matches, in GeFaST's quality Levenshtein mode. Each vertical line corresponds to a group of variants using the same boosting function (e.g. *root*) and boosting type (inner or outer) and depicts the range of adjusted Rand index values covered by varying the boosting parameter (e.g. the degree of the root). The cross marks the clustering quality of the chosen representative of the Converge-A cost function on the different data sets.

Franzén data (Figure S18). The maximum adjusted Rand index could be increased by outer, multiplicative boosting, which also led to a higher precision and a usually lower recall. The average clustering quality was affected similarly but to a lesser extent.
As before, both unboosted variants resulted in essentially the same clustering quality and provided only very small improvements to the Levenshtein mode. On Franzén data, the variant with weighted matches increased the maximum adjusted Rand index by up to 0.3 %. Very high factors were again the preferred parameter for the multiplicative boosting function. The best boosted variant, leading to an improved maximum and average clustering quality on the Franzén data sets, was *v3 / mult / 1000* and the improvements in the maximum adjusted Rand index varied between 0.2 and 1.6 %.

Frith cost function. The Frith cost function also worked with both weighted and unweighted matches, achieving at least the same clustering quality as the Levenshtein mode, but only improved clustering quality slightly when using unweighted matches. These improvements were again restricted to the Franzén data sets. Figure S19 shows that the largest increases in the maximum adjusted Rand index occurred with inner boosting using the linear or multiplicative boosting function with weighted matches and outer multiplicative boosting without weighted matches. The higher adjusted Rand index was usually accompanied by a slightly improved precision and a stable recall. Very similar changes were observed for the average clustering quality. Once more, both unboosted variants attained essentially the same clustering quality, which was very slightly better than the Levenshtein mode. The unboosted variant with weighted matches increased the maximum adjusted Rand index up to 0.3 %. An inspection of the different boosted variants showed that the linear boosting function worked best with small arguments, multiplicative function preferred different arguments depending on the usage of weighted matches. Both boosting functions resulted in a slightly higher clustering quality on the V4 data sets and among those providing improvements on the V3-V4 data sets, the linear boosting function led to slightly better results. On the Callahan data, the best boosted variants usually achieved the same clustering quality as the Levensthein mode. Overall, variants with with weighted matches led to improvements more often and also produced the largest ones. The chosen representative, *v1 / linear / 0*, increased both the the maximum and the average clustering quality on the Franzén data sets, with the improvements of the maximum adjusted Rand index lying between 0.4 and 3.0 %.

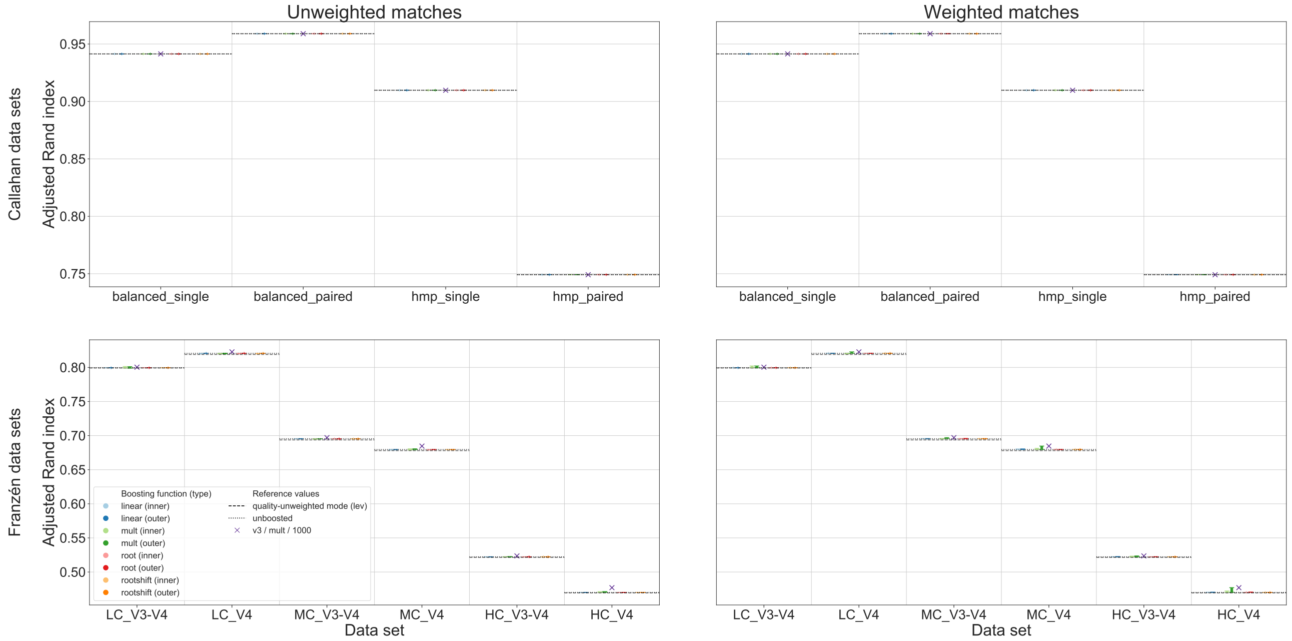

Figure S18: Maximum clustering quality of the examined variants of the quality-weighted Converge-B cost function, with (right column) and without (left) weighted matches, in GeFaST's quality Levenshtein mode. Each vertical line corresponds to a group of variants using the same boosting function (e.g. *root*) and boosting type (inner or outer) and depicts the range of adjusted Rand index values covered by varying the boosting parameter (e.g. the degree of the root). The cross marks the clustering quality of the chosen representative of the Converge-B cost function on the different data sets.

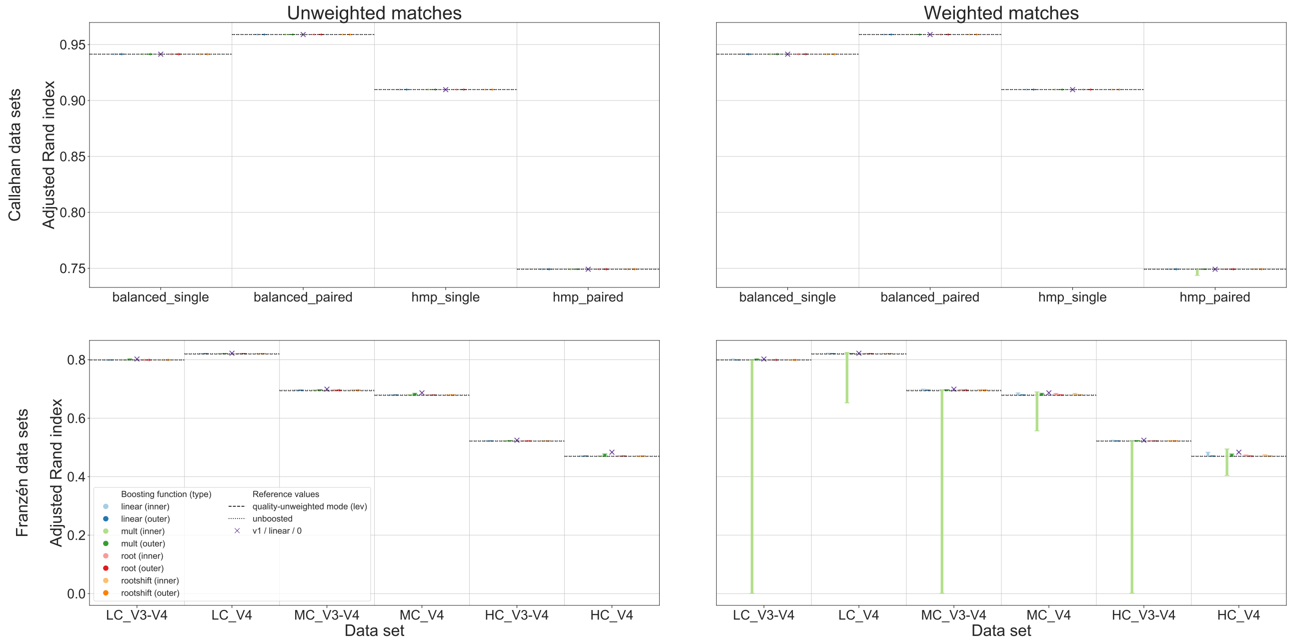

Figure S19: Maximum clustering quality of the examined variants of the quality-weighted Frith cost function, with (right column) and without (left) weighted matches, in GeFaST's quality Levenshtein mode. Each vertical line corresponds to a group of variants using the same boosting function (e.g. *root*) and boosting type (inner or outer) and depicts the range of adjusted Rand index values covered by varying the boosting parameter (e.g. the degree of the root). The cross marks the clustering quality of the chosen representative of the Frith cost function on the different data sets.

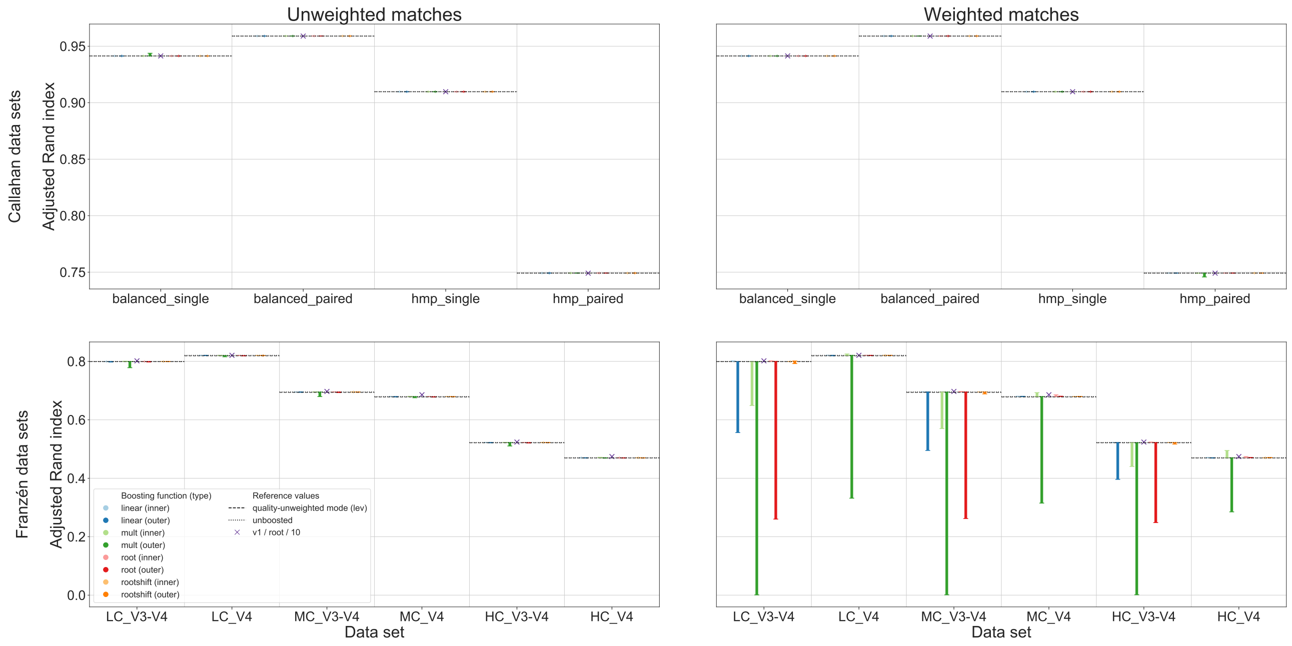

Figure S20: Maximum clustering quality of the examined variants of the quality-weighted Kim-A cost function, with (right column) and without (left) weighted matches, in GeFaST's quality Levenshtein mode. Each vertical line corresponds to a group of variants using the same boosting function (e.g. *root*) and boosting type (inner or outer) and depicts the range of adjusted Rand index values covered by varying the boosting parameter (e.g. the degree of the root). The cross marks the clustering quality of the chosen representative of the Kim-A cost function on the different data sets.

Kim-A cost function. The Kim-A cost function with both weighted and unweighted matches was able to attain results at least as good as the quality-unweighted Levenshtein mode but tended to provide only small improvements in maximum and average clustering quality at best. As can be seen in Figure S20, the boosted cost function was considerably more sensitive to changes of the boosting parameter when weighting matches, with some choices leading to a much lower adjusted Rand index. However, boosted variants with weighted matches also allowed the observed improvements through inner boosting with the multiplicative or the root boosting function. Similar to the other cost functions, an increased adjusted Rand index involved a higher precision and a slightly reduced recall most of the time.

The unboosted variants were as good as or slightly better than the quality-unweighted Levenshtein mode, in terms of both maximum and average adjusted Rand index, and both attained an almost identical clustering quality. On Franzén data, the unboosted variant with weighted matches showed some minute advantages and increased the maximum adjusted Rand index up to 0.3 % compared to the Levenshtein mode. Of the boosted variants, multiplicative reinforcement with high factors (e.g. 250) worked best for the `V4` data sets, increasing the maximum adjusted Rand index up to 5.5 %, but was also notably worse than the Levenshtein mode on `V3-V4` data sets. Root boosting with high degrees could improve the clustering quality on all Franzén data sets but the improvements were smaller on the `V4` data sets (especially on `HC_V4`). For instance, *v1 / root / 10* increased the maximum adjusted Rand index between 0.3 and 0.5 % on the `V3-V4` data sets and between 0.2 and 1.2 % on the `V4` data sets.

Kim-B cost function. The Kim-B cost function behaved almost exactly like the Kim-A version. Improvements in terms of maximum and average clustering quality were similarly possible for both weighted and unweighted matches but were also rather limited in size and scope (Figure S21). Boosted variants with weighted matches and multiplicative reinforcement or root extraction as the inner boosting function were again the best choices to obtain these improvements.

The unboosted variants were once more at least as good as the Levenshtein mode and essentially identical to each other. The unboosted variant with weighted matches increased the maximum adjusted Rand index up to 0.3 % on the Franzén data sets. Multiplicative reinforcement with high factors (e.g. 500) was the best boosting approach for the `V4` data sets, increasing the maximum adjusted Rand index up to 6.6 % but again had problems on `V3-V4` data sets (even though they tended to be smaller than with the Kim-A cost function). As before, root boosting with high degrees was the allrounder on the Franzén data sets, with *v1 / root / 10*

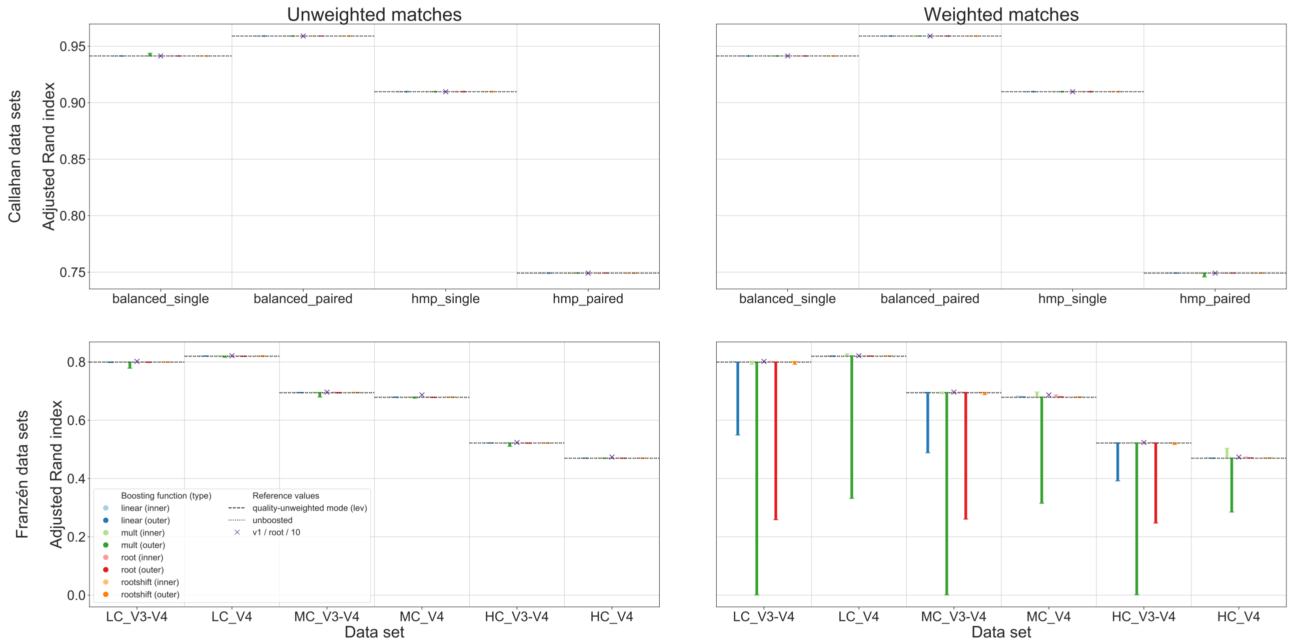

Figure S21: Maximum clustering quality of the examined variants of the quality-weighted Kim-B cost function, with (right column) and without (left) weighted matches, in GeFaST's quality Levenshtein mode. Each vertical line corresponds to a group of variants using the same boosting function (e.g. *root*) and boosting type (inner or outer) and depicts the range of adjusted Rand index values covered by varying the boosting parameter (e.g. the degree of the root). The cross marks the clustering quality of the chosen representative of the Kim-B cost function on the different data sets.

improving the maximum adjusted Rand index by 0.3 to 0.5 % on the `V3-V4` data sets and by 0.2 to 1.2 % on the `V4` data sets.

Malde-A cost function. The Malde-A cost function led to improvements in maximum and average clustering quality on Franzén data both with and without weighted matches. Figure S22 shows how the adjusted Rand index increased, especially when using linear or shifted root boosting as the inner boosting function or when using multiplicative boosting as the outer boosting function, and that no similar improvements were achieved on Callahan data. On the contrary, the better boosted variants of linear and shifted root boosting actually attained a lower clustering quality on `balanced_paired`. On the Franzén data sets, the increased adjusted Rand index was usually accompanied by an improved precision and an at least stable recall.
The maximum and average clustering quality of both unboosted variants of the Malde-A cost function was essentially identical. They increased the maximum adjusted Rand index up to 0.3 %. When weighting matches, linear and shifted root boosting preferred small parameters, while multiplicative boosting worked best with small to medium factors. Without weighted matches, the parameter had no effect on linear and shifted root boosting. The chosen representative, *v3 / mult / 100*, allowed to increase the maximum adjusted Rand index by 2.1 to 4.4 % on Franzén data.

Malde-B cost function. The clustering quality of the Malde-B cost function differed, for the most part, only marginally from the one of the quality-unweighted Levenshtein mode. Still, we observed some, usually small improvements in the maximum and average clustering quality on Franzén data sets (especially the `V4` ones) when weighting matches and applying multiplicative reinforcement as the outer boosting function. Figure S23 also indicates, however, that the boosting parameter has to be chosen carefully because some choices led to a considerably reduced clustering quality. The higher adjusted Rand index usually involved an improved precision and a reduced recall.
Both unboosted variants of the Malde-B cost function attained essentially the same clustering quality and improved only very slightly on the Levenshtein mode. On the Franzén data sets, the unboosted variant with weighted matches increased the maximum adjusted Rand index up to 0.3 %. The best boosted variants used multiplicative reinforcements with very high factors and improved both the maximum and average adjusted Rand index. The best variant, *v1 / mult / 1000*, raised the maximum adjusted Rand index by 0.6 to 6.5 % on

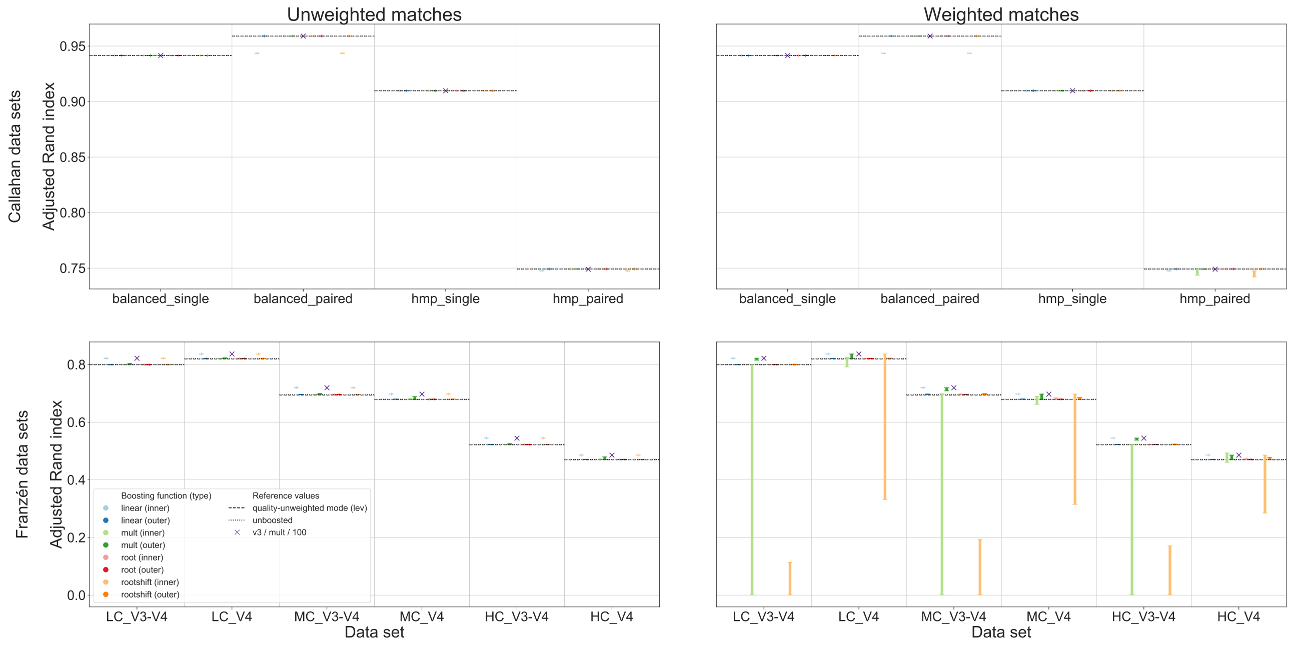

Figure S22: Maximum clustering quality of the examined variants of the quality-weighted Malde-A cost function, with (right column) and without (left) weighted matches, in GeFaST's quality Levenshtein mode. Each vertical line corresponds to a group of variants using the same boosting function (e.g. *root*) and boosting type (inner or outer) and depicts the range of adjusted Rand index values covered by varying the boosting parameter (e.g. the degree of the root). The cross marks the clustering quality of the chosen representative of the Malde-A cost function on the different data sets.

Franzén data.

Malde-C cost function. The Malde-C cost function led to hardly any improvements in the average or maximum clustering quality compared to the quality-unweighted Levenshtein mode. Instead, many variants lowered the clustering quality considerably on Franzén data, especially when using weighted matches (Figure S24). Shifted root boosting and, to some extent, linear and root boosting allowed small improvements in the adjusted Rand index, usually accompanied by a slightly higher precision and a slightly lower recall.
The clustering quality of the two unboosted variants was almost identical and only very slightly better than the one of the Levenshtein mode. On Franzén data, the unboosted variant with weighted matches increased the maximum adjusted Rand index by less than 0.3 %. Of the boosted variants, those with weighted matches and inner, shifted root boosting using a medium to high degree worked best over all data sets, but the effect of the boosting parameter and the difference to outer boosting were very small. Other boosted variants tended to lower the clustering quality on at least one kind of data (often the V3-V4 data sets). The best variant, *v1 / rootshift / 8*, allowed to increase the maximum adjusted Rand index by 0.2 to 0.4 % on the Franzén data sets, while the differences were negligible on the Callahan data sets.

C.2.3 Performance

Runtime. Figure S25 shows that GeFaST, when run in the quality-unaware Levenshtein mode, was as fast as or faster than Swarm and outperformed UPARSE notably (even for high thresholds). Compared to USEARCH and VSEARCH, GeFaST attained a better or similar runtime for all but the few highest thresholds. As expected, the use of quality-weighted cost functions increased the runtime. For large parts of the considered threshold range, the increase observed for the unboosted variants was, however, moderate and the different cost functions behaved quite similarly. Averaged over the whole threshold range, the unboosted variants were between 10 % (Frith) and 27 % (Converge-B, Kim-B, Malde-C) slower than the quality-unaware variant. The unboosted variants were also still faster than the other tools on more than half of the considered threshold range. In contrast, some of the boosted variants increased the runtime more notably, even though most of them were comparable to the unboosted variants. The Clement and Malde-B cost functions were by far the slowest ones. Malde-A also showed a larger increase compared to the remaining cost functions but clearly remained in the lower half of the depicted range for the boosted quality Levenshtein variants. Except for Clement and Malde-

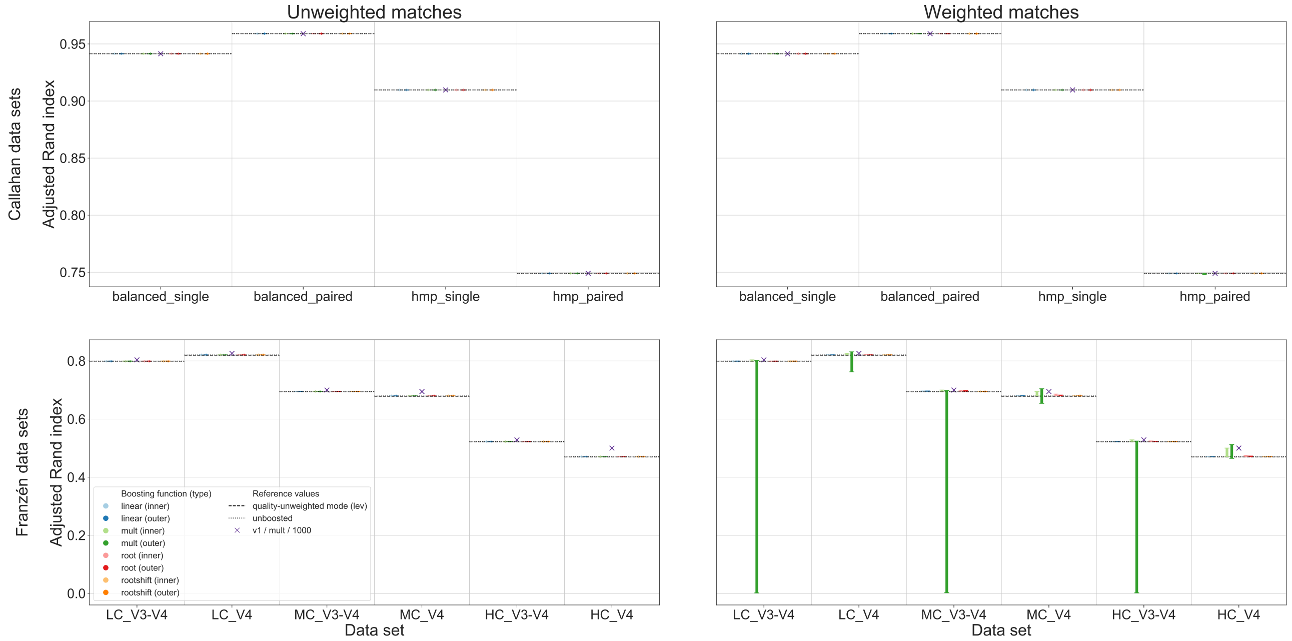

Figure S23: Maximum clustering quality of the examined variants of the quality-weighted Malde-B cost function, with (right column) and without (left) weighted matches, in GeFaST's quality Levenshtein mode. Each vertical line corresponds to a group of variants using the same boosting function (e.g. *root*) and boosting type (inner or outer) and depicts the range of adjusted Rand index values covered by varying the boosting parameter (e.g. the degree of the root). The cross marks the clustering quality of the chosen representative of the Malde-B cost function on the different data sets.

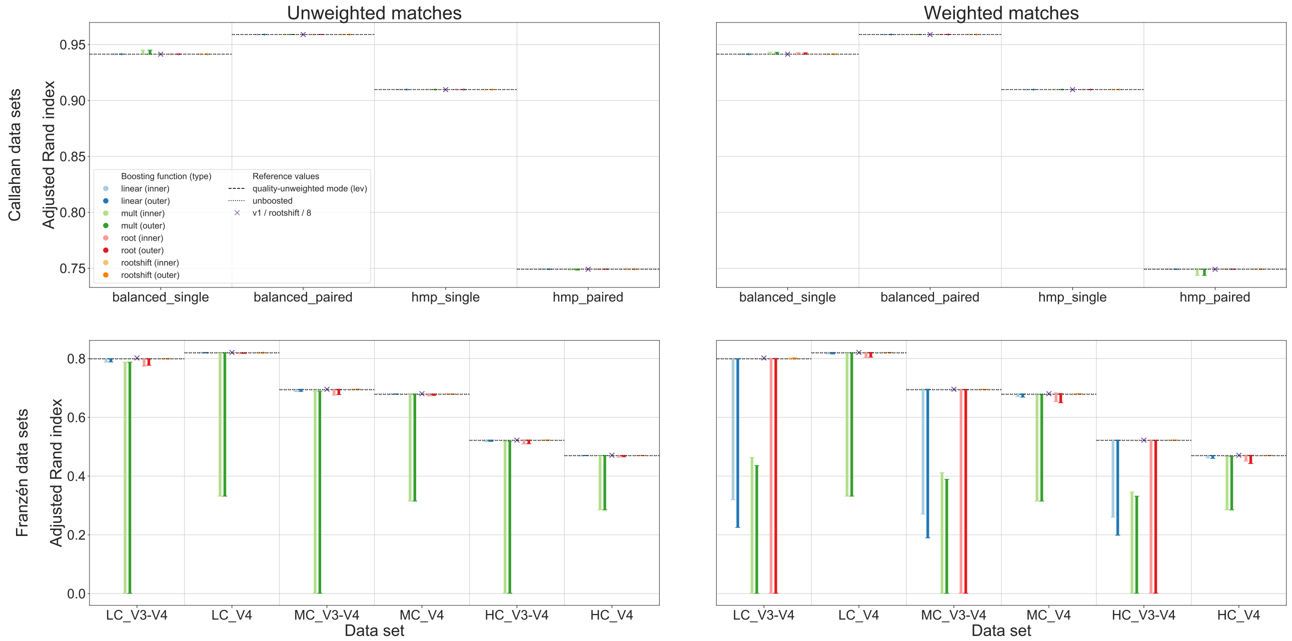

Figure S24: Maximum clustering quality of the examined variants of the quality-weighted Malde-C cost function, with (right column) and without (left) weighted matches, in GeFaST's quality Levenshtein mode. Each vertical line corresponds to a group of variants using the same boosting function (e.g. *root*) and boosting type (inner or outer) and depicts the range of adjusted Rand index values covered by varying the boosting parameter (e.g. the degree of the root). The cross marks the clustering quality of the chosen representative of the Malde-C cost function on the different data sets.

B, boosted variants were between 14 and 70 % slower than the quality-unware variant. As a consequence, the runtime of the boosted variants tended to approach and surpass the runtime of the other tools one or two threshold steps earlier.
The quality-unaware alignment-score mode of GeFaST was slower than its Levenshtein counterpart and comparable to the other tools only for the first three to four threshold steps (except for UPARSE, which was surpassed a few steps later). Except for the Malde-A cost function, all unboosted variants of the quality alignment-score mode were slower than the quality-unaware variant. On average, Malde-A was almost 36 % faster, while the other variants were between 14 % (Frith) and 45 % (Kim-A, Kim-B) slower and the increases became especially notable in the second half of the threshold range. Malde-A was also the fastest boosted variant but its advantage over the other cost functions was much smaller compared to the unboosted case. Most boosted variants were actually faster than the quality-unware one for large parts of the threshold range. On average, the boosted variants of Converge-A, Converge-B, Kim-B, Malde-A and Malde-C were between 1 and 40 % slower, while the others were between 8 and 22 % faster. Therefore, the runtime of the boosted variants was relatively comparable to the one of the other tools in the first half of the threshold range.

Memory consumption. Compared to the other tools, the memory consumption of GeFaST was considerably higher (Figure S26) but this difference was largely caused by the preprocessing step. Here, GeFaST applied the FASTQ preprocessor averaging the error probabilities during the dereplication of the reads, which is more memory-intensive than other preprocessing options. Moreover, the other tools processed a FASTA version of the `hmp_single` data set. In order to obtain a comparable value for GeFaST, we also ran it in a quality-unaware mode using the FASTA file and obtained a memory consumption of approximately 50 MiB, which was very close to VSEARCH. The other tools, especially Swarm and UPARSE, still required notably less memory (down to 30 MiB). The remaining difference mainly results from additional data structures supporting the clustering step and a currently less optimised internal representation of the reads.
Returning to the actual runs of our evaluation, we observed essentially the same memory consumption for both quality-unaware modes. Similarly, there was no difference between the quality Levenshtein and quality alignment-score mode or between the different cost functions. These showed a higher memory consumption than the quality-unaware modes due to the storage of the quality scores. However, there was no increase for larger thresholds, because the memory consumption of the clustering step did not exceed the one of the preprocessing step.

C.3 Model-supported methods

In the following, we provide additional plots and details on the main results described in Section "Clustering with model-supported methods". The full analyses are available as Jupyter notebooks in the evaluation repository. Complementing the visualisation of the maximum clustering quality in Figure S27 in the main text, Figures S28 and S29 show the average and N-best average clustering quality, respectively, of GeFaST in the examined modes, using the different consistency-based methods. Figure S30 depicts the number of clusters constructed by the examined variants, while Figure S31 relates these numbers to the maximum clustering quality.

The descriptions are split based on the used mode of GeFaST. In the alignment-score and Levenshtein mode, the clustering phase was performed by the `ClassicSwarmer` or the `ConsistentClassicSwarmer` and we refer to the corresponding executions as *unchecked* and *checked*, respectively. The cluster refiners `LightSwarmAppender` and `LightSwarmResolver` offer four different options for handling ungrafted light clusters (see Algorithm S3) and we refer to the four variants of the respective refiner by appending the option number to its abbreviation. For example, the `LightSwarmAppender` (LSA) discarding the remaining light swarms (option 2) is referred to as LSA-2.

C.3.1 Alignment-score mode

Unchecked clustering. We first considered the clustering quality of unchecked clustering followed by either fastidious or consistency-based refinement. Both fastidious variants increased the maximum adjusted Rand index compared to the unrefined variant on all data sets (except `paired`, for which it remained largely unchanged). Using a doubled instead of an incremented threshold led to larger improvements on Franzén data. For example, doubling and incrementing increased the adjusted Rand index by 12.0 % and 5.1 % on the `V3-V4` data sets and by 8.7 % and 5.6 % on the `V4` data sets, respectively. On Callahan data, however, the gains were smaller and very similar for both variants (e.g. 1.2 % on the `single` data sets).

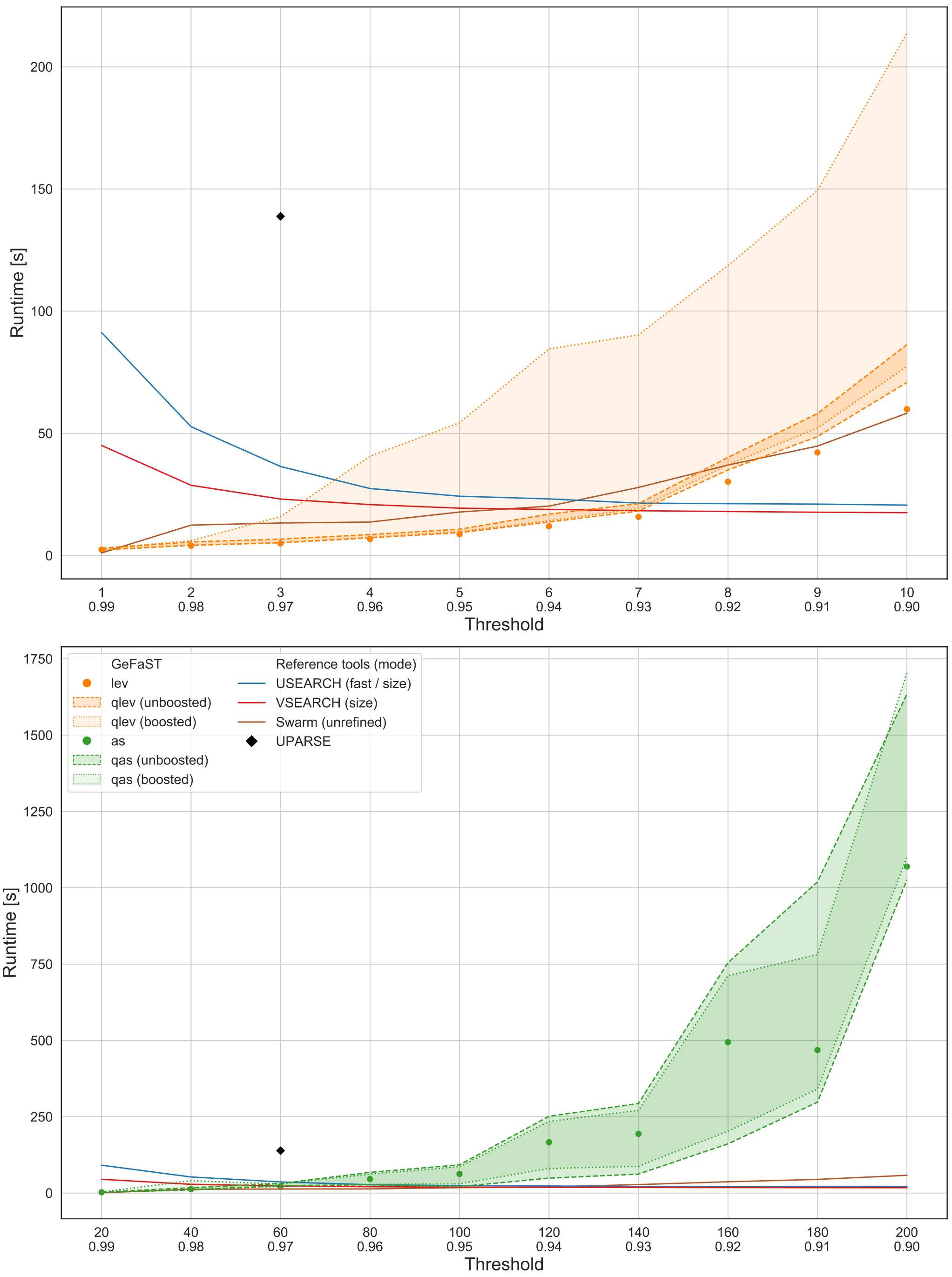

Figure S25: Runtime of GeFaST in quality Levenshtein *(upper)* and quality alignment-score *(lower)* mode on the `hmp_single` data set. Includes the best boosted and unboosted variant of each quality-weighted cost function listed in Table 4. The runtime of the different boosted and unboosted variants is not shown individually but as two ranges. The quality-weighted variants are compared to the respective quality-unaware mode as well as to USEARCH, VSEARCH, UPARSE and Swarm.

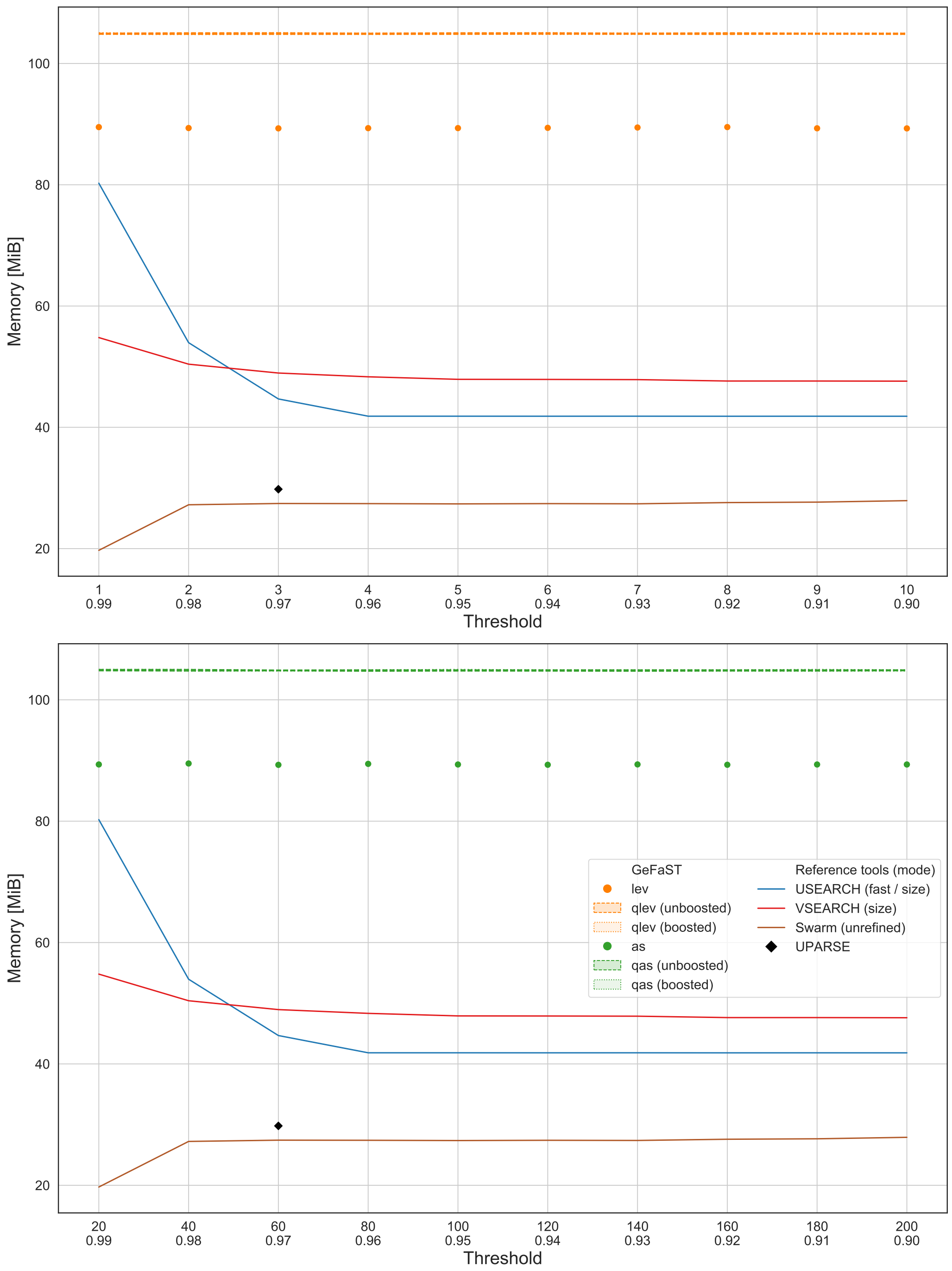

Figure S26: Memory consumption of GeFaST in quality Levenshtein *(upper)* and quality alignment-score *(lower)* mode on the hmp_single data set. Includes the best boosted and unboosted variant of each quality-weighted cost function listed in Table 4. The memory consumption of the different boosted and unboosted variants is not shown individually but as two ranges. The quality-weighted variants are compared to the respective quality-unaware mode as well as to USEARCH, VSEARCH, UPARSE and Swarm.

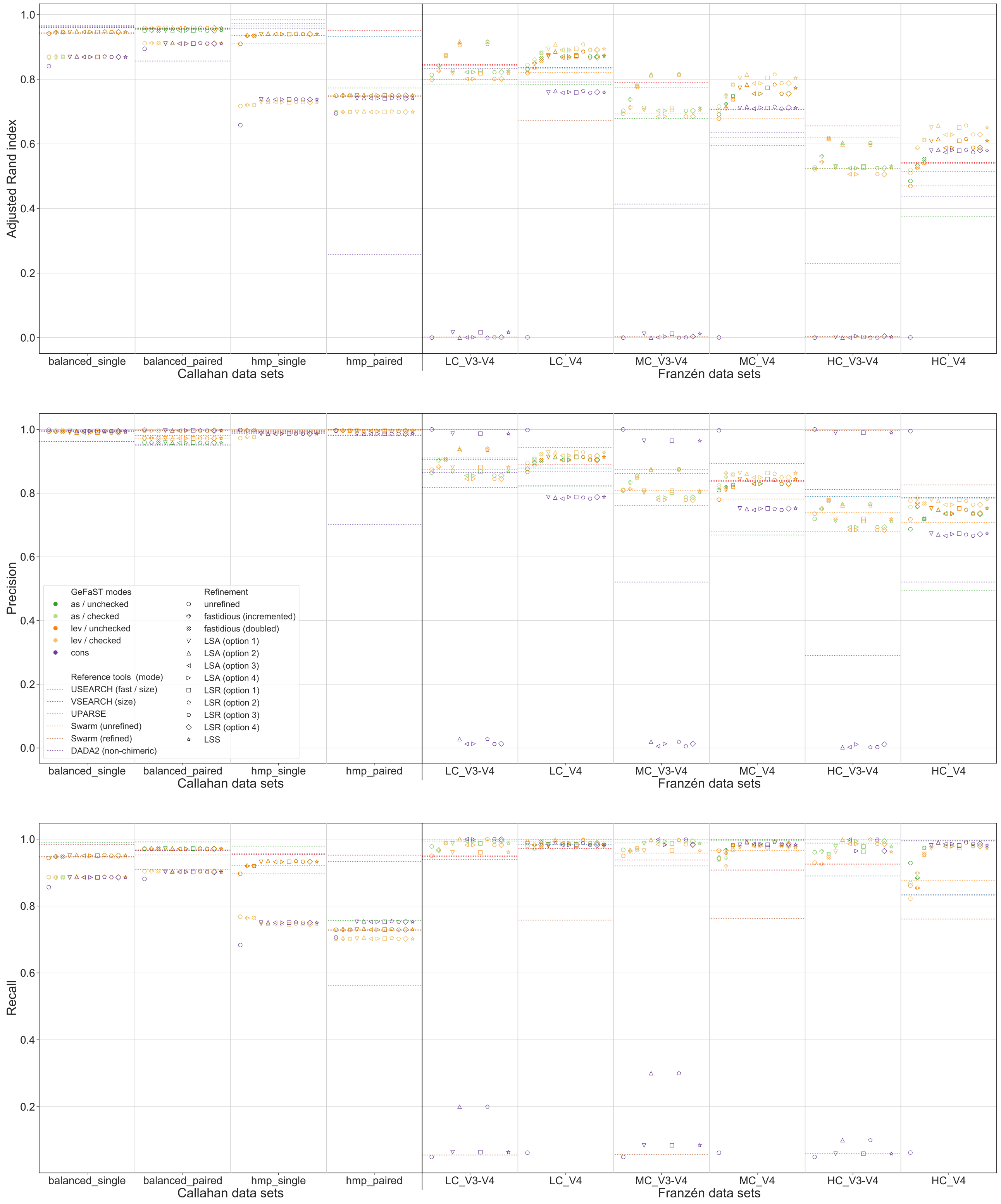

Figure S27: Clustering quality of GeFaST (modes: `as`, `lev`, `cons`), DADA2, USEARCH, VSEARCH, UPARSE and Swarm on the Callahan and Franzén data sets. Alignment-score and Levenshtein mode were evaluated with and without the consistency check in the clustering phase. Shows the maximum adjusted Rand index (and the corresponding precision and recall) of each tool or variant per data set. For Franzén data, the average values of the 10 actual data sets per combination of complexity and read type have been averaged. The plot of the maximum adjusted Rand index corresponds to Figure 6.

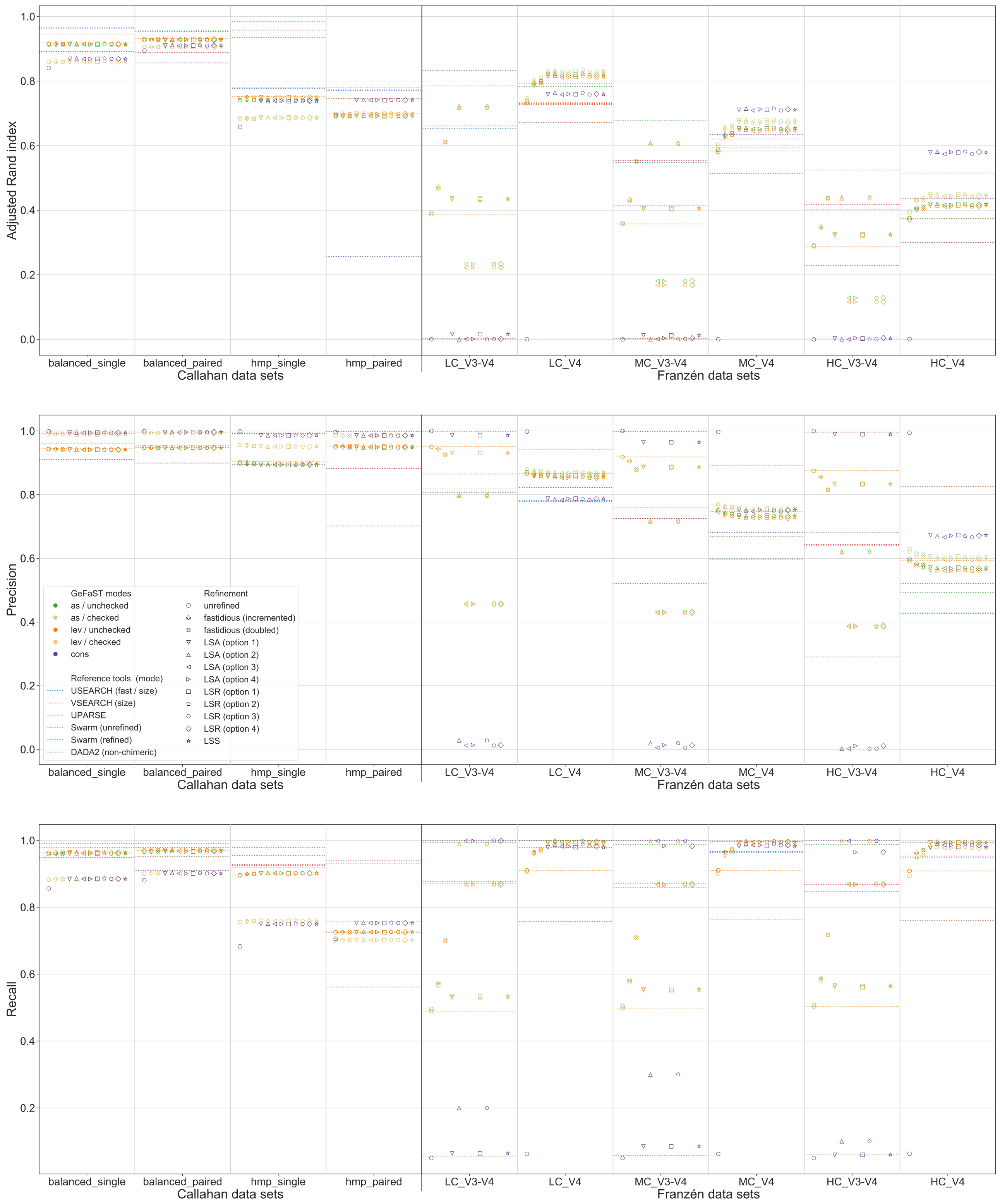

Figure S28: Clustering quality of GeFaST (modes: `as`, `lev`, `cons`), DADA2, USEARCH, VSEARCH, UPARSE and Swarm on the Callahan and Franzén data sets. Alignment-score and Levenshtein mode were evaluated with and without the consistency check in the clustering phase. Shows the average adjusted Rand index (and the corresponding precision and recall) of each tool or variant per data set. For Franzén data, the average values of the 10 actual data sets per combination of complexity and read type have been averaged.

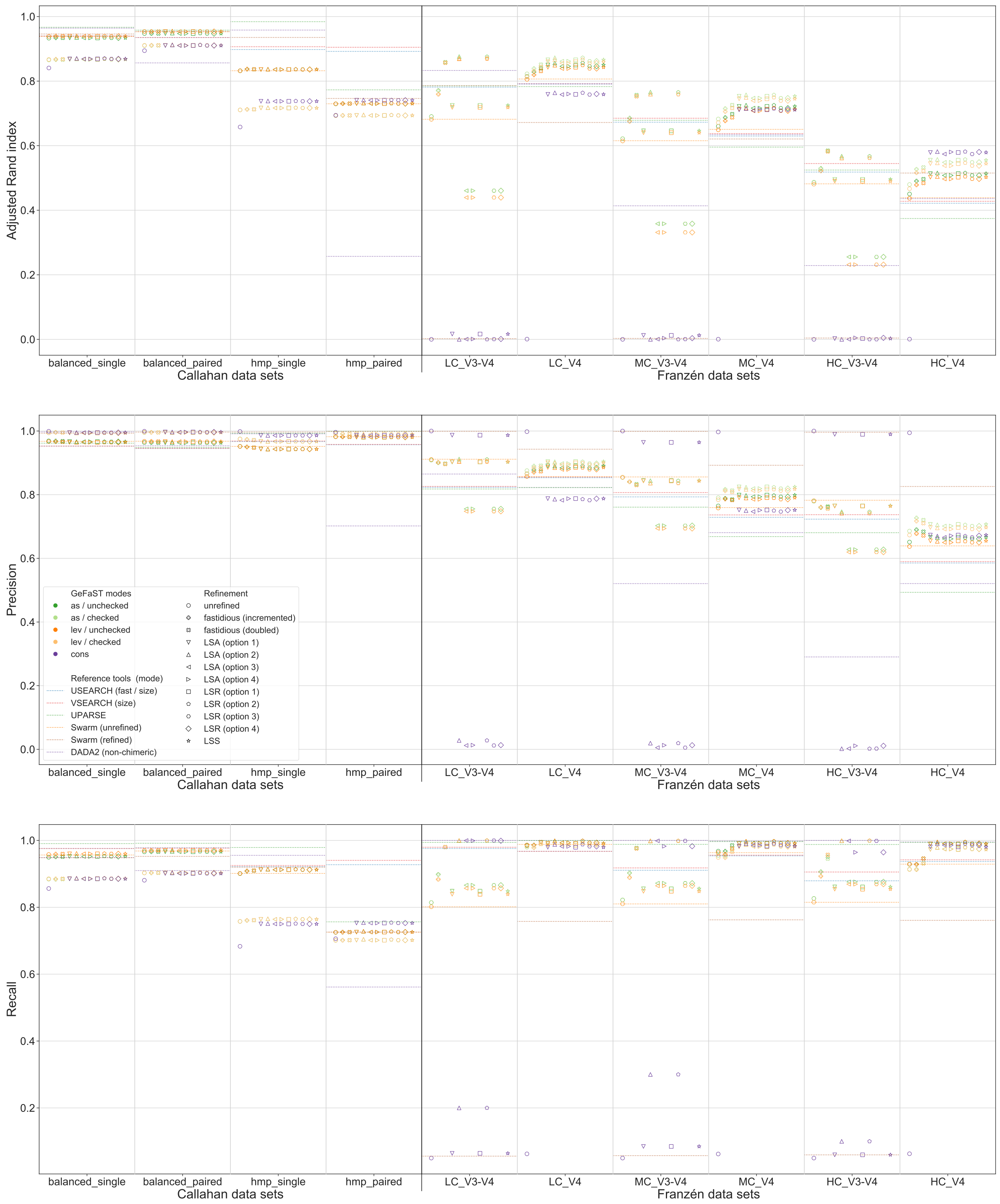

Figure S29: Clustering quality of GeFaST (modes: `as`, `lev`, `cons`), DADA2, USEARCH, VSEARCH, UPARSE and Swarm on the Callahan and Franzén data sets. Alignment-score and Levenshtein mode were evaluated with and without the consistency check in the clustering phase. Shows the N-best average adjusted Rand index (and the corresponding precision and recall) of each tool or variant per data set. For Franzén data, the average values of the 10 actual data sets per combination of complexity and read type have been averaged.

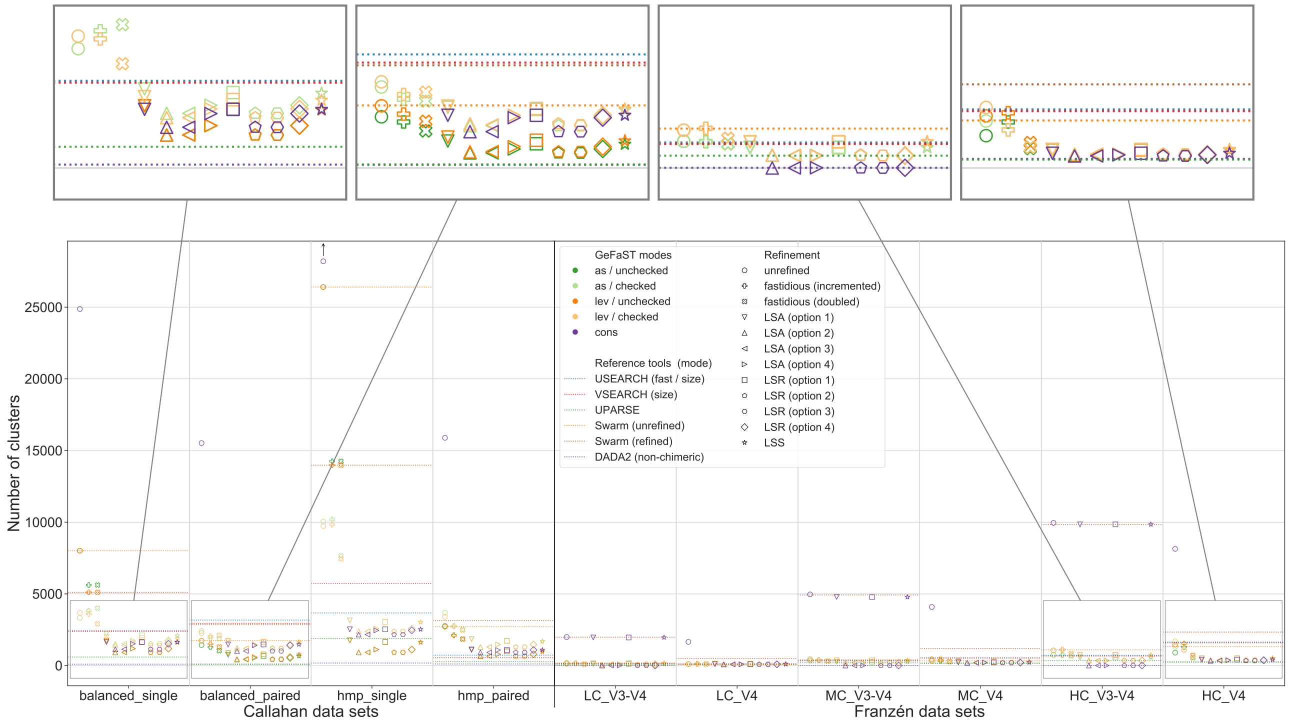

Figure S30: Number of clusters produced by GeFaST (modes: `as`, `lev`, `cons`), DADA2, USEARCH, VSEARCH, UPARSE and Swarm on the Callahan and Franzén data sets. Alignment-score and Levenshtein mode were evaluated with and without the consistency check in the clustering phase. Shows the number of clusters corresponding to the maximum adjusted Rand index attained by each tool or variant per data set. For Franzén data, the values of the 10 actual data sets per combination of complexity and read type have been averaged.

The consistency-based refinement methods also improved the maximum adjusted Rand index on all data sets. Based on their clustering quality, the different variants can be divided into three groups. The first one, comprising LSA-2 and LSR-2, increased the adjusted Rand index the most (approximately 14.4 % on `V3-V4` data and 15.6 % on `V4` data) but also discarded 40 to 70 % of the amplicons on the `V3-V4` data sets, up to 2 % on the `V4` data sets and between 1 and 4 % on the Callahan data sets. Therefore, their clustering quality is not directly comparable to the one of the other, non-discarding variants. The second group contains the variants LSA-1, LSR-1 and LSS. They attained a slightly lower maximum adjusted Rand index on `V4` data (14.2 %) but led to an already much smaller improvement on `V3-V4` data (1.2 %). The remaining variants, forming the third group, provided even smaller gains on `V3-V4` data (0.2 %) and `V4` data (11.8 %). All consistency-based refinement methods improved the adjusted Rand index on the `paired` and `single` data sets by at least 0.04 % and 1.9 %, respectively. Compared to the fastidious variants, the consistency-based refinement methods thus attained a similar or higher maximum clustering quality on all data sets except for the `V3-V4` data.
The increased adjusted Rand index was usually associated with an improved precision on Franzén data (except for the second and third group on `V3-V4` data). With a few exceptions on `LC_V4`, the recall was also higher on all Franzén data sets. Precision and recall did not change for the most part on Callahan data. The largest changes were observed on `hmp_single`, on which the precision decreased slightly while the recall went up notably.
The consistency-based methods can be grouped in the same way when considering the average clustering quality but the differences between the groups were notably less distinct on `V4` data. Except for the third group, all refinement methods also improved the average adjusted Rand index on the Franzén data, while the differences were minute on the Callahan data.

Checked clustering. The repetition of the analysis with checked clustering led to very similar results. Both the fastidious and the consistency-based refinement methods again increased the maximum clustering quality compared to the unrefined variant on all data sets. On `V3-V4` data, the clustering quality and the improvements were essentially identical to the ones observed for unchecked clustering. Refinement after checked clustering, however, consistently increased both on `V4` data. The doubling fastidious variant was still the better fastidious one and improved the maximum adjusted Rand index by 10.6 % compared to the unrefined variant. The consistency-based variants formed the same three groups and led to improvements of 15.9 %, 14.5 % and

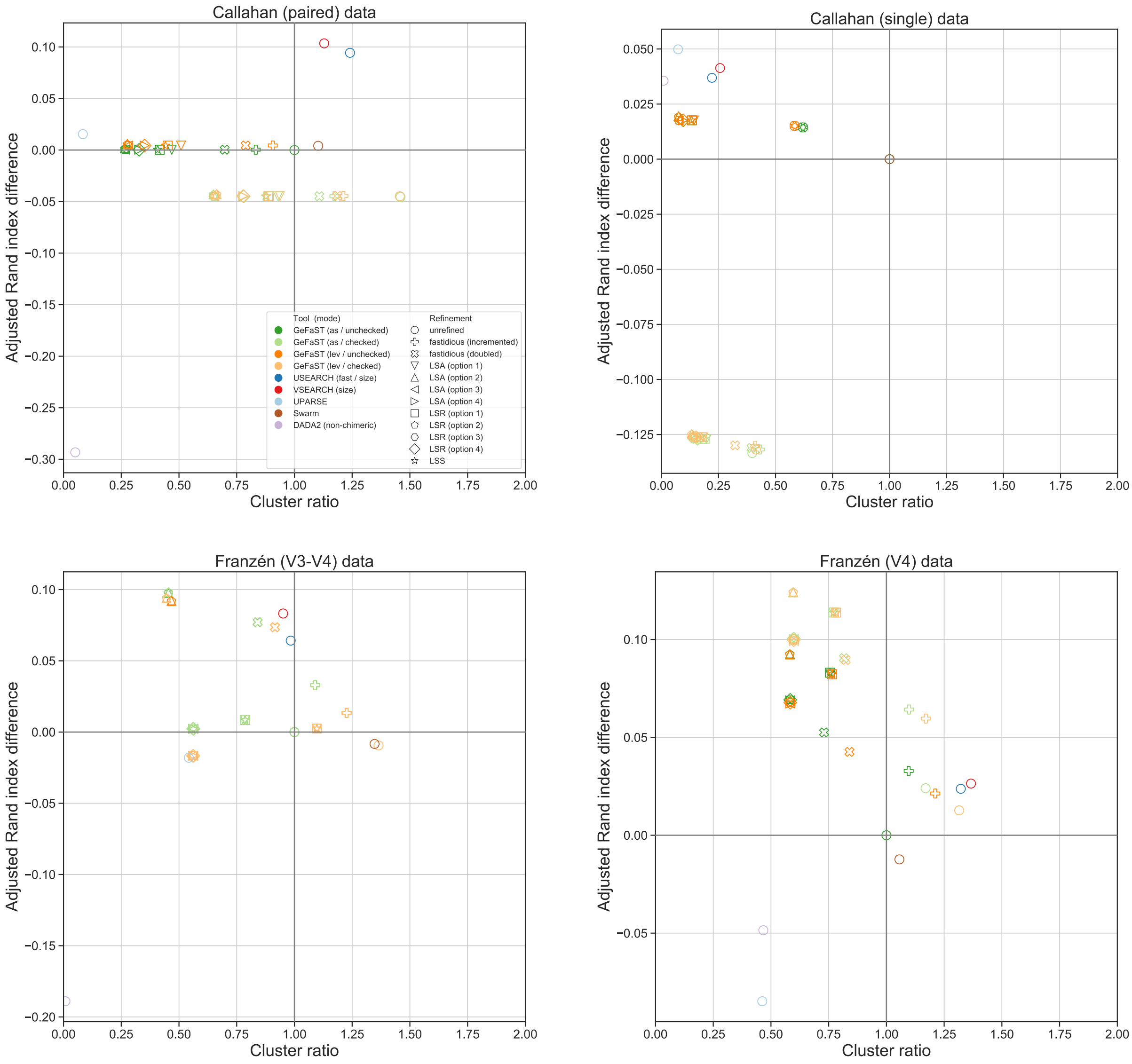

Figure S31: Relation between the number of clusters and the clustering quality of GeFaST (modes: `as`, `lev`), DADA2, USEARCH, VSEARCH, UPARSE and Swarm on Callahan and Franzén data. Alignment-score and Levenshtein mode were evaluated with and without the consistency check in the clustering phase. Uses the unrefined results of the alignment-score mode (unchecked) as the reference point $(1.0, 0.0)$ and shows the ratio of the number of clusters and the difference in the maximum adjusted Rand index of each tool or variant (averaged over the read type). The consistency mode has been omitted to increase the readability, but similar plots including that mode are available in the evaluation notebooks.

12.2 %, respectively. On paired data, the improvements increased very slightly but rarely exceeded 0.1 %, while they even decreased to approximately 1.0 % for the consistency-based variants and to at most 0.3 % for the fastidious ones on single data. In addition, checked clustering (with and without refinement) attained a consistently lower absolute clustering quality on Callahan data compared to unchecked clustering.
As before, the gains in adjusted Rand index on Franzén data were usually accompanied by an increased precision (with some exceptions on V3-V4 data) and recall, while both remained largely unchanged on Callahan data. The most notable effects were again observed on hmp_single, but with a reverse trade-off between precision and recall compared to unchecked clustering.
The different refinement methods had comparable effects on the average clustering quality for unchecked and checked clustering.

Comparison with other tools. Both unchecked and checked clustering followed by any consistency-based refinement variants attained a higher maximum clustering quality than the other tools on V4 data (at least +6.4 %). The fastidious refinement variants also tended to provide better or similar results, while the unrefined variants were at best as good as USEARCH and VSEARCH but still better than Swarm and UPARSE. On V3-V4 data, however, only LSA-2, LSR-2 and the doubling fastidious variant reached an at least similar adjusted Rand index compared to USEARCH and VSEARCH. The other variants showed a maximum adjusted Rand index between 8 and 12 % lower than the one of USEARCH and VSEARCH on V3-V4 data. In contrast, all examined variants of GeFaST again attained a similar or higher clustering quality than Swarm and UPARSE (between 1.2 and 17.4 % higher). USEARCH, VSEARCH and UPARSE, in turn, performed better on the Callahan data, even though the advantage over the variants with unchecked clustering was relatively small (except for hmp_paired). The clustering quality of Swarm was very similar to the one of GeFaST with unchecked clustering.
In comparison to DADA2 even the unrefined variants of GeFaST reached a higher maximum clustering quality on almost all Franzén data sets, with the exception of LC_V3-V4 on which DADA2 was similar to or even better than some variants. Moreover, the clustering quality of variants with unchecked and checked clustering was higher on paired Callahan data, while it was lower on single data (only slightly in the case of unchecked clustering).

Number of clusters at maximum clustering quality. Both consistency-based and fastidious refinement reduced the number of clusters substantially on Franzén and Callahan data, with the larger (relative) reductions usually being observed on Callahan data. The consistency-based methods tended to decrease their number much stronger, regardless of using unchecked or checked clustering. With the exception of option 1, the differences between LSA and LSR were minute in both cases. LSA-2 and LSR-2 led to the largest reductions (from 41.8 % on V4 data to 92.5 % on single data), closely followed by the variants with option 3 and 4 (41.7 % to 92.5 %). LSA-1, LSR-1 and LSS decreased the number of clusters less than the other consistency-based variants, but they still attained reductions between 21.4 and 86.8 %.
On V3-V4 data, unchecked and checked clustering followed by the same refinement method did not differ in the number of clusters. While the consistency-based refiners resulted in fewer clusters, the fastidious methods attained the higher clustering quality (not considering the cluster discarding variants LSA-2 and LSR-2). On all other data categories, unchecked clustering led to an (at least) slightly lower number of clusters and, except for V4 data, an increased or similar clustering quality when compared to checked clustering.
Compared to USEARCH and VSEARCH, GeFaST with refinement (especially the consistency-based methods) resulted in a lower number of clusters on both Callahan and Franzén data. GeFaST without refinement and, to some degree, with fastidious refinement showed no clear tendency, producing more clusters on some data categories (e.g. single) and a similar number or fewer on others (e.g. V4). The number of clusters obtained from the unrefined variants of GeFaST (with unchecked clustering) and Swarm was very much alike due to the methodological similarities of the methods. Swarm with refinement, however, differed from GeFaST with doubling fastidious refinement because the former is limited to a clustering threshold of one. Therefore, it usually produced more clusters than the other methods, which considered multiple thresholds. DADA2, sometimes matched by UPARSE, typically resulted in the lowest number of clusters on the different data sets.

C.3.2 Levenshtein mode

Overall, the general observations and tendencies of the Levenshtein mode were very similar to the ones of the alignment-score mode. Consequently, we mainly address the aspects in which the results of the two modes differed. Apart from that, the preceding descriptions are also valid for the Levenshtein mode.

Unchecked clustering. The improvements in maximum adjusted Rand index of the different variants deviated marginally from the corresponding ones in alignment-score mode. On Franzén data, the relative improvements increased slightly for almost all variants. The consistency-based refinement methods formed the same three groups and ranked in the same order. While all groups provided an at least slightly increased clustering quality on all data categories in alignment-score mode, the variants of the third group decreased the maximum adjusted Rand index by 1.3 % on `V3-V4` data in Levenshtein mode.

Checked clustering. Similar to unchecked clustering, the improvements in the clustering quality for checked clustering were very similar to the ones in the alignment-score mode. Their relative size again increased slightly without changing the grouping and ranking of the variants.

Comparison with other tools. Except for some numerical details, the general observations made for the alignment-score mode remain valid for the Levenshtein mode. The different variants of `GeFaST` in Levenshtein mode attained a slightly lower clustering quality on Franzén data (especially the `V3-V4` data sets), reducing the minimum advantage of consistency-based refinement over the other tools on `V4` data to 6.2 %. At the same time, these refinement methods showed a maximum adjusted Rand index that was up to 14 % lower on `V3-V4` data compared to `USEARCH` and `VSEARCH`. However, the clustering quality of almost all variants of `GeFaST` exceeded the one of the remaining tools.

Number of clusters at maximum clustering quality. The general observations for the alignment-score mode again remain valid for the Levenshtein mode. The relative reductions of the number of clusters due to refinement were often quite similar to the corresponding value for the alignment-score but could also differ more notably (up to double-digit percentages). However, this did not affect the ranking of the different variants and had only minor impacts on the range of reductions observed for the groups of variants.

C.3.3 Consistency mode

Clustering by consistency. In consistency mode, `GeFaST` failed to produce meaningful clusters on `V3-V4` data, leading to a maximum adjusted Rand index close to 0. While the clustering quality was that low for all variants, they differed in the underlying cause. The unrefined variant, LSA-1, LSR-1 and LSS grouped amplicons insufficiently. Consequently, the precision was unusally high and accompanied by an extremely low recall. The remaining variants went to the other extreme and clustered the amplicons too aggressively. This, in turn, led to a precision close to 0 and, in case of option 3 and 4, a very high recall. On `V4` data, the unrefined variant showed a similarly low clustering quality, while the refined variants produced reasonable results. The clustering quality of all refined variants was almost identical, without showing the differences in precision and recall observed on `V3-V4` data. The adjusted Rand index of refined clustering in consistency mode was, however, lower than the one of the (consistency-based) refinement methods in the other examined modes. Nevertheless, the gap to the other ones shrank with increasing data complexity as the consistency mode was more stable. On Callahan data, even the unrefined variant led to meaningful clusters and attained a clustering quality slightly lower than the one of checked clustering in alignment-score and Levenshtein mode. The refined variants again improved the adjusted Rand index notably, partly surpassing the clustering quality of checked clustering but falling short of the one reached by unchecked clustering.
Aside from `V3-V4` data, the higher adjusted Rand index of the refined variants was accompanied by a lower precision and higher recall compared to the unrefined variant. In comparison to the other modes, the precision was lower on `V4` data and similar to the one of checked clustering on Callahan data, while the recall was usually also similar to checked clustering.

Comparison with other tools. The other clustering tools attained a considerably higher maximum clustering quality on `V3-V4` data due to the problems of the consistency mode (regardless of the variant) on these data sets. `DADA2`, the other consistency-based method, also reached a much higher clustering quality but the gap shrank notably due to large drops in clustering quality on data sets of higher complexity. On `V4` data, only the unrefined variant of the consistency mode had similar problems, while the other variants started to surpass the other tools with increasing data complexity. The maximum adjusted Rand index of the refined variants was roughly 9 % lower than the one of `USEARCH` and `VSEARCH` on `LC_V4`, about the same on `MC_V4` and approximately 7 % higher on `HC_V4`. The development compared to `Swarm`, `UPARSE` and `DADA2` was similar, with a smaller gap on `LC_V4` and larger improvements on `MC_V4` and `HC_V4`.
On the Callahan data sets, the maximum adjusted Rand index of the consistency-mode variants was between 1 and 33 % lower than the one of the other clustering tools. All variants of the consistency mode attained an

at least 4 % higher clustering quality than DADA2 on the paired data sets, while but were also at least 10 % worse than DADA2 on the single ones.

Number of clusters at maximum clustering quality. On V3-V4 data, the variants of the consistency mode produced either only a few clusters (unrefined, LSA-1, LSR-1, LSS) or almost as many clusters as there were amplicons (all other variants). Both cases were very different from the other results obtained with GeFaST. On the other data categories, the unrefined variant also led to a multiple of the number of clusters of, e.g., the unrefined variant in alignment-score mode, while the refined variants showed much lower numbers comparable to the other modes of GeFaST. The number of clusters often lay between the corresponding numbers of the consistency-based refinement methods of unchecked and checked clustering. LSA and LSR produced the same number of clusters when used with the same option. Among the refined variants of the consistency mode, option 2 and 3 led to the smallest number of clusters, while option 1 and LSS produced the most clusters. LSA and LSR with option 4 lay between these two groups but tended to be closer to option 1.
Compared to USEARCH and VSEARCH, the consistency mode often created notably fewer clusters (except for some variants on V3-V4 data), while Swarm typically resulted in more clusters. DADA2 and UPARSE, in turn, usually still led to even fewer clusters than GeFaST in consistency mode with refinement but some variants attained quite similar numbers on V4 (and V3-V4) data.

C.3.4 Performance

Consistency-checked clustering increased the runtime of GeFaST in both Levenshtein and alignment-score mode, but checked clustering remained relatively comparable to unchecked clustering and the other tools in the lower half of the threshold range (Figure S32). Averaged over the threshold range, however, the runtime of the Levenshtein mode was more than doubled, while it increased by 60 % for the alignment-score mode. Clustering by consistency alone was even slower and, of all the unrefined variants, only the alignment-score mode for the highest thresholds surpassed it. Checked clustering also affected the runtime of the subsequent refinement step due to the much higher number of clusters. Fastidious refinement was between 20 and 98 % slower when following on checked clustering, while the runtime of consistency-based refinement increased by up to 180 % (alignment-score mode) resp. 380 % (Levenshtein mode). While the runtime penalty of the consistency-based refinement methods was extremely large for small thresholds, they became comparable to and even quicker than the fastidious methods for the larger thresholds. The runtime of the different consistency-based refiners was very comparable to each other when following on the same clustering step.
As described for the quality-weighted methods, the memory consumption of GeFaST was notably higher than the one of the other tools. Levenshtein and alignment-score mode were again largely similar and the refinement step had essentially no impact on the memory consumption (Figure S33). In contrast to the quality-weighted methods, however, the threshold partially affected it. For larger thresholds, the memory occupied by the variants using checked clustering started to increase as they exceeded the memory consumption of the preprocessing step. In general, the data structures supporting the clustering step are more memory-intensive for larger thresholds and together with an increased memory consumption due to the higher number of clusters (compared to unchecked clustering), the effect became visible for Levenshtein and alignment-score mode with checked clustering. Again similar to the quality-weighted methods, the variants involving quality-aware clustering or refinement occupied more memory due to the storage of the quality scores. For the same reason, the consistency mode required more memory than the completely quality-unaware variants of the Levenshtein and alignment-score mode. However, its memory consumption was below the one of the other quality-aware variants because it can omit some of the data structures used to speed up the distance-based modes.

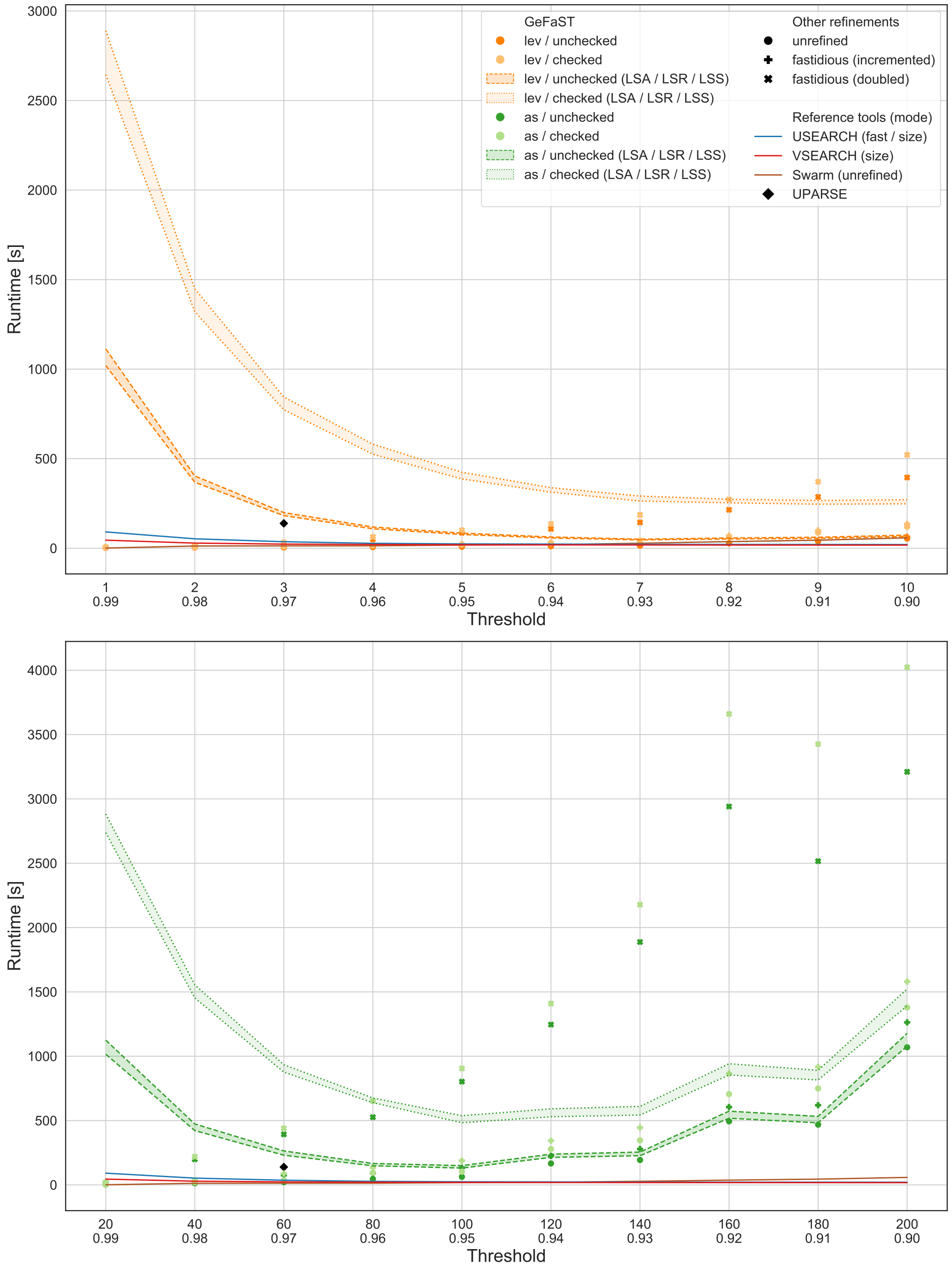

Figure S32: Runtime of GeFaST in Levenshtein *(upper)* and alignment-score *(lower)* mode on the `hmp_single` data set when using model-supported methods in the clustering or refinement phase or both. The runtime of the different variants involving consistency-based refinement methods following on unchecked resp. checked clustering is not shown individually but as two ranges. Also includes unchecked and checked clustering without refinement and followed by fastidious refinement. The variants of GeFaST are compared to USEARCH, VSEARCH, UPARSE and Swarm. GeFaST in consistency mode is not shown here. The unrefined variant clustered the data in 455 seconds, while the refinement variants required between 6349 and 6644 seconds.

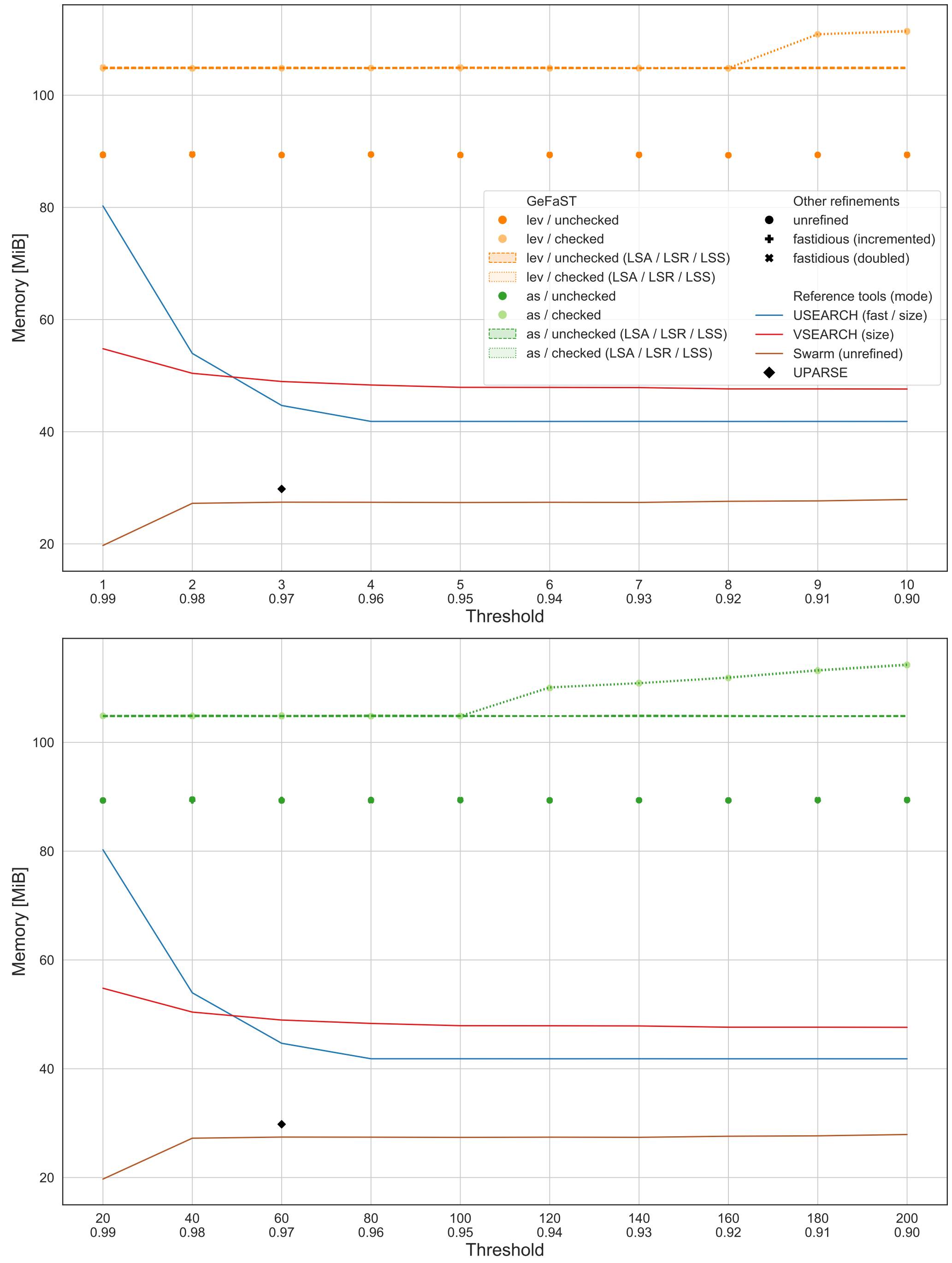

Figure S33: Memory consumption of GeFaST in Levenshtein *(upper)* and alignment-score *(lower)* mode on the `hmp_single` data set when using model-supported methods in the clustering or refinement phase or both. The memory consumption of the different variants involving consistency-based refinement methods following on unchecked resp. checked clustering is not shown individually but as two ranges. Also includes unchecked and checked clustering without refinement and followed by fastidious refinement. The variants of GeFaST are compared to USEARCH, VSEARCH, UPARSE and Swarm. GeFaST in consistency mode is not shown here. All variants occupied approximately 96 MiB.

References

[1] Nathan L. Clement, Quinn Snell, Mark J. Clement, Peter C. Hollenhorst, Jahnvi Purwar, Barbara J. Graves, Bradley R. Cairns, and W. Evan Johnson. The GNUMAP algorithm: unbiased probabilistic mapping of oligonucleotides from next-generation sequencing. *Bioinformatics*, 26(1):38–45, 2009.

[2] Martin C. Frith, Raymond Wan, and Paul Horton. Incorporating sequence quality data into alignment improves DNA read mapping. *Nucleic Acids Research*, 38(7):e100, 2010.

[3] Martin C. Frith, Ryota Mori, and Kiyoshi Asai. A mostly traditional approach improves alignment of bisulfite-converted DNA. *Nucleic Acids Research*, 40(13):e100, 2012.

[4] Temple F. Smith, Michael S. Waterman, and Walter M. Fitch. Comparative Biosequence Metrics. *Journal of Molecular Evolution*, 18(1):38–46, 1981.

[5] Yi-Kuo Yu, John C. Wootton, and Stephen F. Altschul. The compositional adjustment of amino acid substitution matrices. *Proceedings of the National Academy of Sciences*, 100(26):15688–15693, 2003.

[6] Ketil Malde. The effect of sequence quality on sequence alignment. *Bioinformatics*, 24(7):897–900, 2008.

[7] Kwangbaek Kim, Minhwan Kim, and Youngwoon Woo. A DNA sequence alignment algorithm using quality information and a fuzzy inference method. *Progress in Natural Science*, 18(5):595–602, 2008.

[8] Robert Müller and Markus E. Nebel. GeFaST: An improved method for OTU assignment by generalising Swarm's fastidious clustering approach. *BMC Bioinformatics*, 19(1):321, 2018.

[9] Oscar Franzén, Jianzhong Hu, Xiuliang Bao, Steven H. Itzkowitz, Inga Peter, and Ali Bashir. Improved OTU-picking using long-read 16S rRNA gene amplicon sequencing and generic hierarchical clustering. *Microbiome*, 3:43, 2015.

[10] Todd Z. DeSantis, Philip Hugenholtz, Neils Larsen, Mark Rojas, Eoin L. Brodie, Keith Keller, Thomas Huber, Daniel Dalevi, Ping Hu, and Gary L. Andersen. Greengenes, a Chimera-Checked 16S rRNA Gene Database and Workbench Compatible with ARB. *Applied and Environmental Microbiology*, 72(7):5069–5072, 2006.

[11] Weichun Huang, Leping Li, Jason R. Myers, and Gabor T. Marth. ART: a next-generation sequencing read simulator. *Bioinformatics*, 28(4):593–594, 2011.

[12] Eric P. Nawrocki, Diana L. Kolbe, and Sean R. Eddy. Infernal 1.0: inference of RNA alignments. *Bioinformatics*, 25(10):1335–1337, 2009.

[13] Benjamin J. Callahan, Paul J. McMurdie, Michael J. Rosen, Andrew W. Han, Amy Jo A. Johnson, and Susan P. Holmes. DADA2: High-resolution sample inference from Illumina amplicon data. *Nature Methods*, 13(7):581–583, 2016.

[14] Melanie Schirmer, Umer Z. Ijaz, Rosalinda D'Amore, Neil Hall, William T. Sloan, and Christopher Quince. Insight into biases and sequencing errors for amplicon sequencing with the Illumina MiSeq platform. *Nucleic Acids Research*, 43(6):e37, 2015.

[15] James J. Kozich, Sarah L. Westcott, Nielson T. Baxter, Sarah K. Highlander, and Patrick D. Schloss. Development of a dual-index sequencing strategy and curation pipeline for analyzing amplicon sequence data on the MiSeq Illumina sequencing platform. *Applied and environmental microbiology*, 79(17):5112–5120, 2013.
